# Regiodivergent synthesis of functionalized pyrimidines and imidazoles through phenacyl azides in deep eutectic solvents

**DOI:** 10.3762/bjoc.16.158

**Published:** 2020-08-05

**Authors:** Paola Vitale, Luciana Cicco, Ilaria Cellamare, Filippo M Perna, Antonio Salomone, Vito Capriati

**Affiliations:** 1 Dipartimento di Farmacia-Scienze del Farmaco, Università di Bari “Aldo Moro”, Consorzio C.I.N.M.P.I.S., Via E. Orabona 4, I-70125 Bari, Italy; 2 Dipartimento di Scienze e Tecnologie Biologiche ed Ambientali, Università del Salento, Prov.le Lecce-Monteroni, 73100 Lecce, Italy; 3 Dipartimento di Chimica, Università di Bari “Aldo Moro”, Via E. Orabona 4, I-70125 Bari, Italy

**Keywords:** deep eutectic solvents, imidazoles, phenacyl azides, phenacyl halides, pyrimidines

## Abstract

We report that phenacyl azides are key compounds for a regiodivergent synthesis of valuable, functionalized imidazole (32–98% yield) and pyrimidine derivatives (45–88% yield), with a broad substrate scope, when using deep eutectic solvents [choline chloride (ChCl)/glycerol (1:2 mol/mol) and ChCl/urea (1:2 mol/mol)] as environmentally benign and non-innocent reaction media, by modulating the temperature (25 or 80 °C) in the presence or absence of bases (Et_3_N).

## Introduction

In a world with dwindling petroleum resources, the setting up of more and more sustainable routes for the preparation of heterocyclic compounds is an ongoing synthetic endeavor as these scaffolds are ubiquitous motifs in many biologically active compounds and pharmaceuticals. In this context, in the last decades, the so-called deep eutectic solvents (DESs) have received an increasing attention due to their biodegradability, high thermal stability, non-flammability, and low volatility. These are mixtures usually obtained from the combination of 2 or 3 safe, inexpensive and nature-inspired components able to engage in reciprocal hydrogen-bond interactions, and that form fluids at a specified mixing ratio at the desired temperature. Owing to the broad tunability of their physicochemical properties and the ability to act not only as solvents but also as catalysts and reagents, DESs have progressively replaced toxic and volatile organic solvents (VOCs) in countless heterocyclodehydration processes and multicomponent reactions (MCRs) [1–3].

**Scheme 1:**
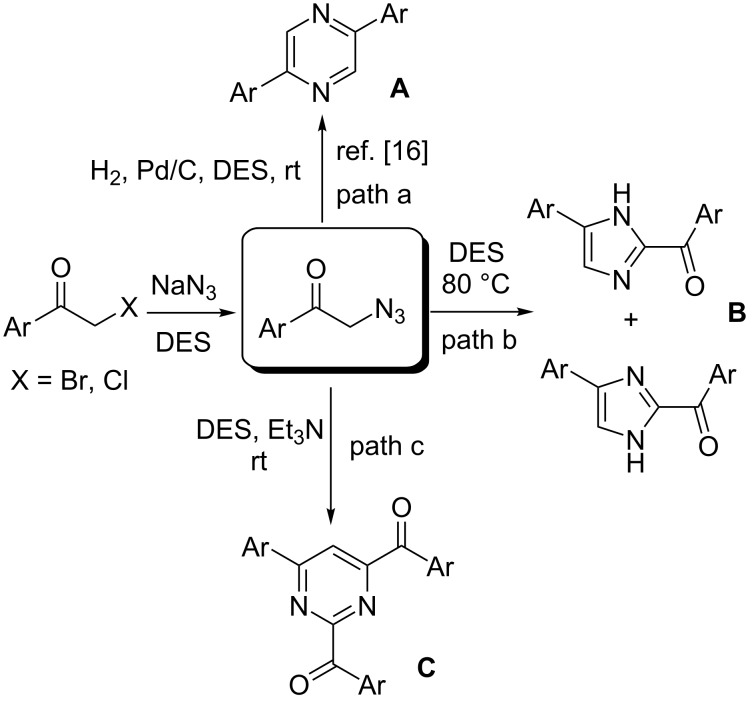
One-pot synthesis of 2,5-diarylpyrazines (**A**) (path a) or 2-aroyl-(4 or 5)-aryl-(1*H*)-imidazoles (**B**) (path b), or 2,4-diaroyl-6-arylpyrimidines (**C**) (path c) in DES from phenacyl azides (rt = room temperature).

As part of our ongoing research in DES chemistry, we reported recently on the preparation of valuable heterocycles by (a) nucleophilic substitution (tetrahydrofuran derivatives) [4], (b) heterocyclodehydration reactions (2-aminoimidazoles, 2-pyrazinones, benzoxazines, thiophenes) [5–8], (c) carbon–sulfur bond-forming reactions [9], (d) directed *ortho*-metalation and nucleophilic acyl substitution strategies [10], (e) Pd-catalyzed aminocarbonylation of aryl iodides, Suzuki–Miyaura and Sonogashira cross-coupling reactions [11–13], (f) Cu-catalyzed C–N coupling reactions [14], and (g) heterogeneous “click” cycloaddition reactions [15] using DESs as environmentally responsible and non-innocent reaction media. Telescoped, one-pot transformations of phenacyl halides to symmetrical 2,5-disubstituted pyrazines (**A**), through phenacyl azides as intermediates, were also found to take place smoothly using such neoteric solvents (Scheme 1, path a) [16].

Among nitrogen-containing heterocyles, imidazoles and pyrimidines are important structural scaffolds commonly found in natural products [17–18], light-emitting devices [19–20], and pharmacologically active compounds as anticancer, anti-inflammatory, antitubercular, antihypertensive, antihistaminic, anti-obesity, antiviral, and other medicinal agents [21–27]. Herein, we wish to report that either 2-aroylimidazoles (**B**) (Scheme 1, path b) or 2,4-diaroylpyrimidines (**C**) (Scheme 1, path c) can regioselectively be prepared from the same phenacyl azide as starting material by properly selecting the nature of the eutectic mixture and the temperature, in the presence or absence of bases.

To the best of our knowledge, while there are a few reports for the synthesis of 2-aroylimidazoles (a) through the condensation of α-azido ketones in iPrOH in the presence of potassium ethylxanthate as a catalyst [28], (b) by exploiting the reaction of arylglyoxals with an excess amount of ammonium acetate in water [29], (c) by the cathodic reduction of 2-azido-1-phenylethanone in a DMF/LiClO_4_ medium [30], (d) by radical chain reactions of α-azido ketones with tributyltin hydride [31], or (e) by a modified Radziszewski’s synthesis when using phenylglyoxals, benzaldeydes, and ammonium acetate as ammonia source in acetic acid or methylene chloride or *N*,*N*-dimethylformamide as the solvent [32], there are no adequate studies covering the preparation of aroylpyrimidines. In 1995, Yamamoto et al. reported the direct introduction of acyl groups into pyrimidine rings by reacting trimethylstannyl derivatives with acylformyl chlorides in dry benzene under a N_2_ stream [33].

## Results and Discussion

**Scheme 2:**
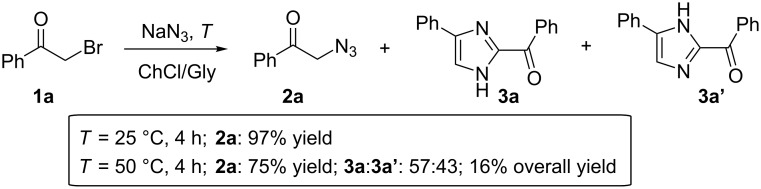
Transformation of phenacyl bromide (**1a**) in ChCl/Gly into phenacyl azide (**2a**) and 2-benzoyl-(4 or 5)-phenyl-(1*H*)-imidazole (**3a** and **3a'**) depending on the temperature.

During our studies on the synthesis of symmetrical pyrazines, we observed that phenacyl bromide (**1a**, 1.5 mmol) could be almost quantitatively converted into phenacyl azide (**2a**, 97% yield), within 4 h, when treated with NaN_3_ (1.65 mmol) as the azide source in a choline chloride (ChCl)/glycerol (Gly) (1:2 mol/mol) eutectic mixture at room temperature (rt, 25 °C) (Scheme 2) [16]. By warming the mixture up to 50 °C, the yield of **2a** dropped to 89% after 1 h, and we noticed in the crude the appearance of an additional product, which was detected as a fluorescent spot on TLC, whose amount increased by increasing the temperature. After 4 h warming at 50 °C, we were able to isolate by column chromatography on silica gel a product which was characterized as 2-benzoyl-(4 or 5)-phenyl-(1*H*)-imidazole (**3a**/**3a'**, Scheme 2). This adduct was formed as a mixture of two tautomers (**3a** and **3a'**; **3a**/**3a'** ratio: 57:43, Supporting Information File 1) [28–29] in an overall 16% yield, the remaining being mainly **2a**, which was isolated in 75% yield (Scheme 2). We thus became interested in improving the yield of **3a**/**3a'**.

After careful evaluation of the reaction parameters (temperature and time) and the amount of NaN_3_ used, we found that the treatment of phenacyl chloride (**1b**) with NaN_3_ (1.5 equiv) in ChCl/Gly at 80 °C gave the best results, as it provided the adducts **3a**/**3a'** in an overall 88% yield after 12 h reaction time (Table 1, entries 1–4). Of note, imidazoles **3a**/**3a'** were found to precipitate directly from the aforementioned eutectic mixture during the reaction, and thus they could be isolated by simple decantation or centrifugation and washing with a few drops of EtOAc or Et_2_O. This procedure left azide **2a** in solution. The latter could be quantified (10% yield) by simple dilution with an equal volume mixture of water and EtOAc, followed by the separation of the organic layer from water, and removal of the volatiles under reduced pressure.

**Table 1:** Optimization of the reaction conditions for the synthesis of **3a**/**3a'**.^a^

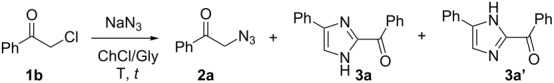					

entry	*T* (°C)	*t* (h)	NaN_3_ (equiv)	**2a** yield (%)^b^	**3a**/**3a'** yield (%)^b^

1	80	4	1.2	37	55
2	100	16	1.2	7	60^c^
3	80	16	0.9	68	32
4	80	12	1.5	10	88

^a^Reaction conditions: **1b** (0.5 mmol), ChCl/Gly (1.0 g); ^b^yield refers to products isolated after column chromatography on silica gel; ^c^15% of 2,4-dibenzoyl-6-phenylpyrimidine was also isolated (see main text and Table 2).

**Scheme 3:**
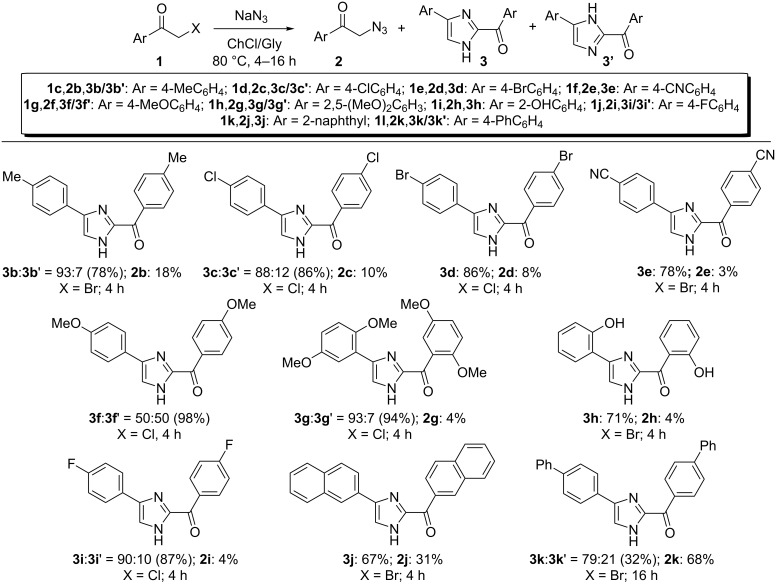
Synthesis of 2-aroyl-(4 or 5)-aryl-(1*H*)-imidazoles **3**. Scope of the reaction. Typical conditions: **1** (0.5 mmol), NaN_3_ (0.75 mmol), ChCl/Gly (1.0 g), 80 °C, 4–16 h; yield refers to products isolated after column chromatography on silica gel; only one tautomer has been depicted for simplicity; imidazoles **3c**/**3c'**, **3i**/**3i'**, **3j**, **3k**/**3k**' were found to precipitate as they formed, and were isolated by filtration/centrifugation and washing with a few drops of AcOEt or Et_2_O; synthesis of imidazoles **3k**/**3k'**: 10% (w/w) EtOH was added to the eutectic mixture to improve the solubility of **2k**.

To examine the scope and limitation of this transformation, various functionalized phenacyl halides (**1c**–**i**) were tested as substrates. As can be seen from the results compiled in Scheme 3, the reaction is amenable to “neutral” (Me), electron-withdrawing (Cl, Br, CN) and electron-donating (MeO, OH) substituents as the desired imidazoles **3b**/**3b'–3h** were isolated in 78–98% yield. The presence of additional halogen groups such as chlorine and bromine in **3c**/**3c'** and **3d** allows further downstream diversification by cross-coupling reactions. A 4-fluoro-substituted phenacyl chloride as well as a bromomethyl 2-naphthyl ketone proved to be competent substrates as well, thus furnishing the corresponding imidazoles **3i**/**3i'** and **3j** in 67–87% yield. Conversely, a phenacyl halide decorated with an additional phenyl group at the *para*-position delivered the expected adduct **3k**/**3k**' in 32% yield even by prolonging the reaction time to 16 h, most probably because of the poor solubility in the eutectic mixture and/or the higher thermal stability towards the loss of N_2_ (vide infra) of the corresponding phenacyl azide **2k** as it is formed. The latter was indeed isolated as the main product (68% yield, Scheme 3).

**Scheme 4:**
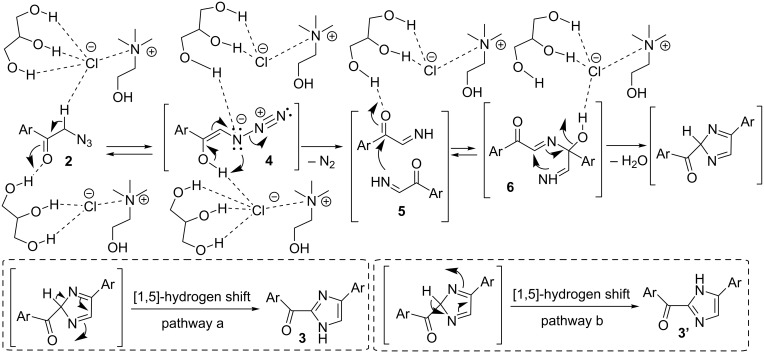
Proposed mechanism for the formation of 2-aroyl-(4 or 5)-aryl-(1*H*)-imidazoles **3**/**3'** from α-phenacyl azides **2** in ChCl/Gly.

A plausible mechanism for the formation of the 2-aroylimidazoles **3**/**3'** is depicted in Scheme 4. A key intermediate may be the in situ generated α-imino ketone **5**. The latter is known to undergo dimerization to give **6** followed by dehydration. The two tautomers **3**/**3'** would most probably originate by two competitive pathways (a and b; Scheme 4) via a [1,5]-hydrogen shift [28–29]. Hydrogen-bond catalysis promoted by DES components may be playing an important role in favoring the loss of nitrogen under mild conditions from a putative azide tautomer **4**, the latter deriving from the corresponding α-phenacyl azide precursor **2** via an acid-catalyzed enolization process [34]. The pyrolysis of α-azido ketones in conventional VOCs (trichlorobenzene) is known to take place under harsh conditions, which are based on heating the mixture between 180 °C and 240 °C [35].

The investigation of the thermal stability of phenacyl azides **2** led to the fortuitous discovery of the competitive formation of a different heterocycle. Indeed, while phenacyl azide (**2a**) was stable per se in a ChCl/Gly mixture after stirring at rt for 12 h, a new fluorescent spot was detected on TLC plate in the presence of an excess of Et_3_N (3 equiv). After column chromatography on silica gel, we were able to isolate a new product that was characterized as 2,4-dibenzoyl-6-phenylpyrimidine (**7a**, 55% yield) in addition to **3a**/**3a'** (26% yield), the remaining being starting material only (conversion: 81%, Table 2, entry 1). The employment of ChCl/urea (1:2 mol/mol) as the eutectic mixture provided **7a** in 57% yield and **3a**/**3a'** in 43% yield, but with full conversion (Table 2, entry 2). By lowering the equivalents of Et_3_N up to 1, or alternatively using K_2_CO_3_ (3 equiv) as a base, in ChCl/urea, the yield of **7a** dropped down up to 33% and that of **3a**/**3a'** up to 25% (Table 2, entries 3–5). By changing the solvent to pure Gly, **7a** and **3a**/**3a'** now formed in 52 and 37% yield, respectively (Table 2, entry 6). When Et_3_N (3 equiv) was alternatively used as the sole solvent, the formation of **7a** was suppressed dramatically (27% yield, Table 2, entry 7) [36]. This last result is consistent with a synergistic cooperation of the basic ChCl/urea eutectic mixture [37] with Et_3_N in promoting the transformation at rt. α-Azido ketones are, indeed, known to be highly base sensitive and to undergo a base-promoted loss of nitrogen to form α-imino ketones upon protonation [38].

**Table 2:** Investigation of the reaction conditions in the synthesis of **7a**.^a^

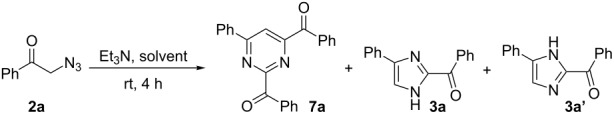				

entry	solvent	**7a** yield (%)^b^	**3a**/**3a'** yield (%)^b^	conversion yield (%)

1	ChCl/Gly^c^	55	26	81
2	ChCl/urea^c^	57	43	full
3	ChCl/urea^d^	41	33	74
4	ChCl/urea^e^	37	25	62
5	ChCl/urea^f^	33	31	66
6	Gly^c^	52	37	89
7	Et_3_N^c^	27	–	27

^a^Reaction conditions: **2a** (0.3 mmol), solvent (0.5 g); ^b^yield refers to products isolated after column chromatography on silica gel; ^c^Et_3_N: 3 equiv; ^d^Et_3_N: 2 equiv; ^e^Et_3_N: 1 equiv; ^f^K_2_CO_3_: 3 equiv.

**Scheme 5:**
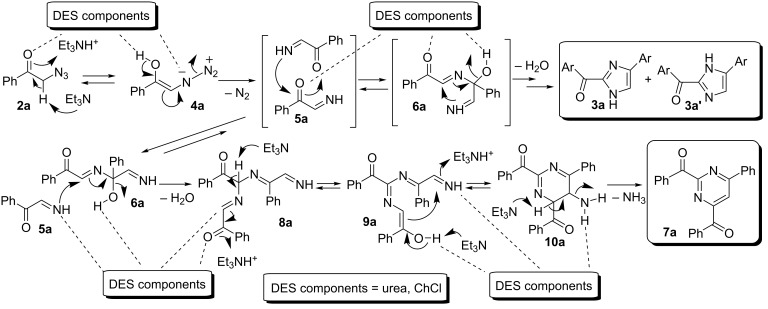
Proposed mechanism for the formation of 2-benzoyl-(4 or 5)-phenyl-(1*H*)-imidazoles **3a**/**3a'** and 2,4-dibenzoyl-6-phenylpyrimidine (**7a**) from α-phenacyl azide (**2a**) in ChCl/urea in the presence of Et_3_N.

A plausible mechanism for the formation of pyrimidine derivative **7a** from **2a**, in competition with imidazoles **3a**/**3a'**, is depicted in Scheme 5.

**Scheme 6:**
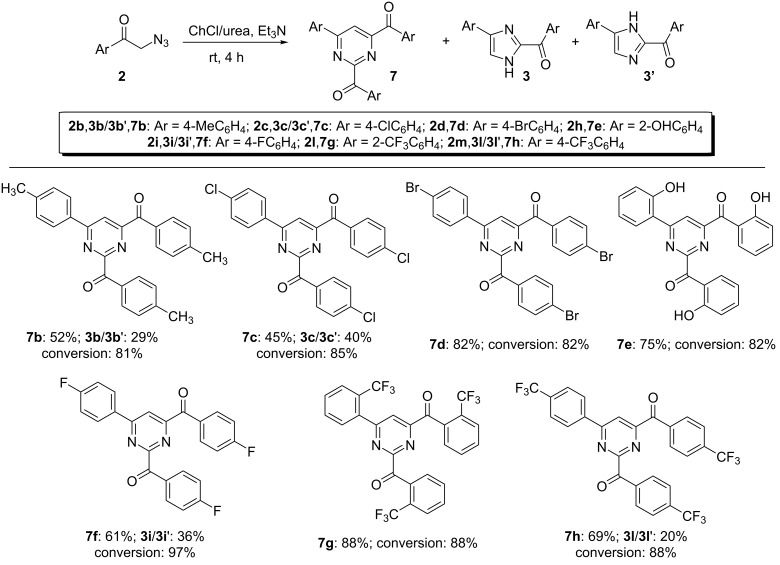
Scope of the synthesis of 2,4-diaroyl-6-arylpyrimidines **7**. Typical conditions: **2** (0.3 mmol), Et_3_N (0.9 mmol), ChCl/urea (0.5 g), rt, 4 h; yield refers to products isolated after column chromatography on silica gel; synthesis of pyrimidine derivatives **7b** and **7d**, reaction time: 12 h.

The key intermediate **5a**, formed by elimination of nitrogen from the enol-azide **4a**, would undergo either dimerization to give **6a**, and then imidazoles **3a**/**3a'** after cyclization and dehydration (see Scheme 5), or trimerization to afford adduct **8a** further to an additional attack of **5a** to the internal imino group of **6a** and dehydration. Consecutive tautomerization, followed by an intramolecular nucleophilic attack to the terminal imino group of **9a**, provides cyclized adduct **10a**, and finally pyrimidine derivative **7a** by aromatization/elimination of NH_3_. To the best of our knowledge, this is the first one-pot synthesis of functionalized pyrimidines using phenacyl azides as the sole starting material [39]. Variation of the phenacyl azides was next investigated in the preparation of various pyrimidines **7** (Scheme 6).

The results shown in Scheme 6 demonstrate that this protocol allows the use of phenacyl azides as starting material also for the preparation of a variety of 2,4,6-trisubstituted pyrimidines. The cyclotrimerization of phenacyl azides containing an alkyl group (4-Me, **2b**), halides (4-Cl, 4-Br and 4-F, **2c**, **d**, and **2i**) or strong electron-withdrawing groups (2-CF_3_, 4-CF_3_, **2l**, **m**) occurs smoothly at rt generally within 4 h in a ChCl/urea eutectic mixture, thereby providing the desired pyrimidines **7b**–**h** in 45–88% yield. Variable amounts of difunctionalized imidazoles **3**/**3'** (20–40%), deriving from a dimerization process, also formed competitively in some cases under the aforementioned conditions. The latter, however, could be easily isolated by column chromatography. The presence of strong electron-donating groups like MeO was not tolerated. Indeed, phenacyl azides **2f**, and **2g** remained unreacted when stirred in ChCl/urea at rt even for 12 h.

## Conclusion

In summary, we have shown that phenacyl halides can be straightforwardly converted, via phenacyl azides, into valuable 2-aroyl-(4 or 5)-aryl-(1*H*)-imidazoles when a solution in ChCl/Gly (1:2) is heated to 80 °C for 4–16 h in the presence of NaN_3_ (1.5 equiv). In most cases, their isolation can be performed by a very gentle procedure: decantation/centrifugation as soon as they form from the above eutectic mixture. The reaction proceeds in very good yields (67–98%) when phenacyl azides are soluble in the eutectic mixture and is applicable to a range of substrates. Phenacyl azides, in turn, can also be competitively converted into 2,4-diaroyl-6-arylpyrimidines (45–88% yield) via an unprecedented cyclotrimerization reaction the key intermediate α-imino ketone undergoes when a solution in ChCl/urea is stirred at rt for 4 h in the presence of Et_3_N (3 equiv) as a base. These cyclizations proved to be relatively sensitive to the electronic properties of the starting phenacyl azides as they did not take place in the presence of strong electron-donating groups like MeO. Studies to expand even more the scope and the selectivity of such DES-promoted heterocyclization reactions and to elucidate their mechanism are in progress.

## Experimental

### General methods

Deep eutectic solvents [choline chloride (ChCl)/glycerol (Gly) (1:2 mol/mol); choline chloride (ChCl)/urea (1:2 mol/mol)] were prepared by heating under stirring up to 80 °C for 10–15 min the corresponding individual components until a clear solution was obtained. ^1^H NMR and ^13^C NMR spectra were recorded on a Varian Mercury 300 or on a Bruker 400 or 600 MHz spectrometer and chemical shifts are reported in parts per million (δ); CDCl_3_, (CD_3_)_2_CO and (CD_3_)_2_SO were used as solvents. GC–MS analyses were performed on a HP 6890 gas chromatograph, Series II by using a HP1 column (methyl siloxane; 30 m × 0.32 mm × 0.25 μm film thickness) equipped with a mass-selective detector operating at 70 eV. Analytical thin-layer chromatography (TLC) was carried out on pre-coated 0.25 mm thick plates of Kieselgel 60 F_254_; visualization was accomplished by UV light (254 nm) or by spraying with a solution of 5% (w/v) ammonium molybdate and 0.2% (w/v) cerium(III) sulfate in 100 mL 17.6% (w/v) aq. sulfuric acid and heating to 473 K until blue spots appeared. Column chromatography was conducted by using silica gel 60 with a particle size distribution of 40–63 μm and 230–400 ASTM, using hexane/EtOAc mixtures as the eluent. High-resolution mass spectrometry (HRMS) analyses were performed using a Bruker microTOF QII mass spectrometer equipped with an electrospray ion source (ESI). NaN_3_, 2-haloketones, and all other reagents, unless otherwise specified, were purchased from Sigma-Aldrich (Sigma-Aldrich, St. Louis, MO, USA), and used without any further purification.

### Experimental procedures

#### General procedure for the synthesis of 2-azido ketones **2d**, **2e**, **2f**, **2k**, **2l** and **2m**

α-Haloketone **1** (1.5 mmol) and NaN_3_ (107 mg, 1.65 mmol) were sequentially added to the ChCl/Gly (1:2 mol/mol, 2.0 g) eutectic mixture. The reaction mixture was stirred at 25 °C under air for 3–12 h until complete consumption of the starting material (monitored by thin-layer chromatography). Then, water was added, and the mixture was extracted with EtOAc (3 × 10 mL). The collected organic layers were dried using anhydrous Na_2_SO_4_, filtered, and the volatile evaporated under reduced pressure to afford the crude product, which was purified by column chromatography on silica gel (hexane/EtOAc 5:1–4:1) to afford the desired α-azido ketone **2**. Characterization data of the isolated 2-azido ketones are provided in Supporting Information File 1.

#### Synthesis of 2-benzoyl-4-phenyl-1*H*-imidazole (**3a**) and 2-benzoyl-5-phenyl-1*H*-imidazole (**3a'**). Typical procedure


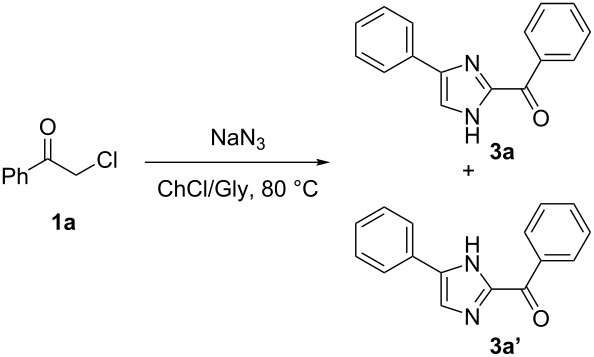


Sodium azide (0.75 mmol) was added to a solution of 2-chloroacetophenone (**1a**, 0.5 mmol) in ChCl/Gly (1:2 mol/mol, 1.0 g) under air and with vigorous stirring. The mixture was then warmed to 80 °C. After 12 h, 10 mL of water were added and the solid **3a** was recovered by decantation (or filtration) from the reaction mixture and washed with a few drops of EtOAc or Et_2_O. The solution was extracted with EtOAc (3 × 10 mL). The combined organic phases were dried over anhydrous Na_2_SO_4_ and the solvent was concentrated in vacuo. The addition of Et_2_O to the crude mixture allowed the further precipitation of **3a**, which was finally recovered in 88% yield. Characterization data of the isolated imidazoles are provided in Supporting Information File 1.

#### Synthesis of 2,4-dibenzoyl-6-phenylpyrimidine (**7a**). Typical procedure


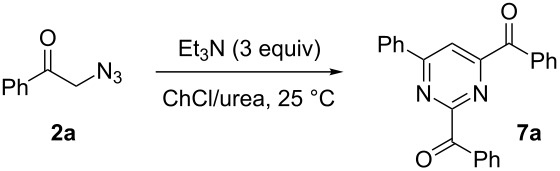


Et_3_N (0.930 mmol, 0.130 mL) was added to a solution of 2-azidoacetophenone **(2a**, 0.31 mmol, 0.05 g) in ChCl/ urea (1:2 mol/mol, 0.5 g) eutectic mixture at 25 °C, under air and with vigorous stirring. The reaction was monitored by TLC (hexane/EtOAc 7:3). After 4 h, 5 mL of water were added and the reaction mixture was extracted with EtOAc (3 × 5 mL). The combined organic phases were dried over anhydrous Na_2_SO_4_ and the solvent was concentrated under reduced pressure. Purification by column chromatography on silica gel (hexane/EtOAc 9:1) provided pyrimidine **7a** in 57% yield. A mixture of imidazoles **3a**/**3a'** (43% yield) was also isolated. Characterization data of the isolated pyrimidines are provided in Supporting Information File 1.

## Supporting Information

File 1 Compound characterization data and NMR spectra.
