# Supplementary material for: Regiodivergent synthesis of functionalized pyrimidines and imidazoles through phenacyl azides in deep eutectic solvents
Source: Beilstein J Org Chem. 2020 Aug 5;16:1915–23. doi: 10.3762/bjoc.16.158 (PMC7418094; doi:10.3762/bjoc.16.158)

BEILSTEIN JOURNAL OF ORGANIC CHEMISTRY

Supporting Information

for

Regiodivergent synthesis of functionalized pyrimidines and imidazoles through phenacyl azides in deep eutectic solvents

Paola Vitale, Luciana Cicco, Ilaria Cellamare, Filippo M. Perna, Antonio Salomone and Vito Capriati

Beilstein J. Org. Chem. **2020,** *16,* 1915–1923. doi:10.3762/bjoc.16.158

Compound characterization data and NMR spectra

License and Terms: This is a supporting information file under the terms of the Creative Commons Attribution License (http://creativecommons.org/licenses/by/4.0). Please note that the reuse, redistribution and reproduction in particular requires that the authors and source are credited.
The license is subject to the *Beilstein Journal of Organic Chemistry* terms and conditions: (https://www.beilstein-journals.org/bjoc)

Table of contents

1. **Compound characterization data** S3

    1.1 Spectroscopic data of 2-azido ketones **2d**, **2e**, **2f**, **2k, 2l,** and **2m** S3

    1.2 Spectroscopic data of 2-aroyl-4-aryl-1*H*-imidazoles **3a**–**3k** and 2-aroyl-5-aryl-1*H*-imidazoles **3a'**–**3c'**, **3f'**, **3g'**, **3i'**, and **3k'** S5

    1.3 Spectroscopic data of 2,4-diaroyl-6-arylpyrimidines **7a**–**h** S9

    1.4 References S11

2. **NMR spectra** S12

    2.1 NMR spectra of 2-azido ketones **2d**, **2e**, **2f**, **2k, 2l**, **2m** S12

    2.2 NMR spectra of 2-aroyl-4-aryl-1*H*-imidazoles **3a**–**3k** and 2-aroyl-5-aryl-1*H*-imidazoles **3a'**–**3c'**, **3f'**, **3g'**, **3i'**, and **3k'** S18

    2.3 NMR spectra of 2,4-diaroyl-6-arylpyrimidines **7a**–**h** S29

1. Compound characterization data

2-Azido ketones **2a**, **2b**, **2c**, **2g**, **2h, 2i** and **2j** were prepared as reported [1]. Spectroscopic data of α-azido ketones **2d**, **2e**, **2f**, **2k, 2l** and **2m** are in agreement with those reported in the literature [2-5].

1.1 Spectroscopic data of α-azido ketones 2d, 2e, 2f, 2k, 2l and 2m

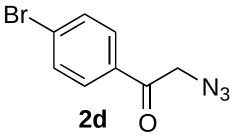

2d

2-Azido-1-(4-bromophenyl)ethanone (**2d**) [2]. Orange solid (yield: 86%). ^{1}H NMR (600 MHz, $CDCl_3$) δ 7.77 (d, *J* = 8.5 Hz, 2 H), 7.64 (d, *J* = 8.5 Hz, 2 H), 4.53 (s, 2H). ^{13}C NMR (150 MHz, $CDCl_3$) δ 192.3, 133.0, 132.3, 129.4, 129.3, 54.7. FT IR (KBr): 2905, 2851, 2104, 1694, 1584, 1568, 1485, 1401, 1342, 1283, 1219, 1178, 1069, 996, 911, 806, 721 cm^{-1}. HRMS calcd for $C_8H_6BrN_3ONa$ [M +Na]$^+$: 261.9586. Found: 261.9579.

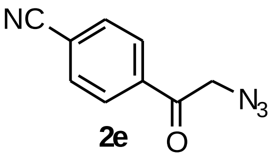

2e

4-(2-Azidoacetyl)benzonitrile (**2e**) [3]. Brown solid (yield: 83%). ^{1}H NMR (600 MHz, $CDCl_3$) δ 8.02 (d, *J* = 7.3 Hz, 2 H), 7.82 (d, *J* = 7.3 Hz, 2 H), 4.58 (s, 2 H). ^{13}C NMR (150 MHz, $CDCl_3$) δ 192.1, 137.2, 132.8, 132.7, 129.4, 128.4, 117.5, 117.4, 55.1. FT-IR (KBr): 3097, 2954, 2914, 2231, 2107, 1694, 1606, 1402, 1342, 1293, 1273, 1217, 1004, 914, 832, 765 cm^{-1}. GC-MS (70 eV) *m/z* (%): 158 [(M-28)$^+$, 1], 131 (15), 130 (100), 102 (43), 75 (11), 51 (6). HRMS calcd for $C_9H_5N_4O$ [M-H]$^-$: 185.0469. Found: 185.0365.

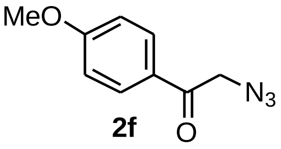

2f

2-Azido-1-(4-methoxyphenyl)ethanone (**2f**) [3]. Yellow solid (yield: 79%). ^{1}H NMR (600 MHz, $CDCl_3$) δ 7.90 (d, *J* = 8.9 Hz, 2 H), 6.97 (d, *J* = 8.9 Hz, 2 H), 4.51 (s, 2 H), 3.89 (s, 3 H). ^{13}C NMR (150 MHz, $CDCl_3$) δ 191.6, 164.2, 130.3, 127.4, 114.2, 55.6, 54.6. FT-IR (KBr): 3031, 2922, 2851, 2124, 1683, 1600, 1517, 1454, 1421, 1361, 1302, 1272, 1240, 1178, 1025, 945, 825, 770 cm^{-1}. GC MS (70 eV) *m/z* (%) 163 [(M-28)$^+$, 1], 135 (100), 92 (17), 77 (21), 64 (10), 63 (9). HRMS calcd for $C_9H_9N_3O_2$ [M+Na]$^+$: 214.0587. Found: 214.0582.

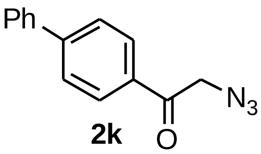

2k

2-Azido-1-([1,1'-biphenyl]-4-yl)ethanone (**2k**) [4,5]. Dark yellow solid (yield: 67%). ^{1}H NMR (600 MHz, $CDCl_3$) δ 8.08 (d, *J* = 8.4 Hz, 2 H), 7.73 (d, *J* = 8.4 Hz, 2 H), 7.84 (m, 2 H), 7.49 (m, 2 H), 7.43 (t, *J* = 7.3 Hz, 1 H), 4.5 (s, 2 H). ^{13}C NMR (150 MHz, $CDCl_3$) δ 192.8, 146.7, 139.4, 133.0, 129.1, 128.9, 128.5, 127.5, 127.2, 54.9 FT-IR (KBr): 3080, 2960, 2100, 1684 1604, 1581, 1460, 1447, 1420, 1276, 1226, 1191, 1116, 1075, 911, 847, 832, 809 cm^{-1}. HRMS calcd for $C_{14}H_{11}N_3O$ [M+Na]$^+$: 260.0794. Found: 260.0800.

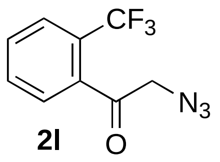

2l

2-Azido-1-[2-(trifluoromethyl)phenyl]ethanone (**2l**) [2]. Colourless oil (yield: 71%). ^{1}H NMR (600 MHz, $CDCl_3$) δ 7.77–7.75 (m, 1 H), 7.68–7.61 (m, 2 H), 7.47–7.42 (m, 1 H), 4.33 (s, 2 H). ^{13}C NMR (150 MHz, $CDCl_3$) δ 197.9, 136.9, 132.2, 131.2, 127.3, 127.2 (q, $^{3}J_{C\text{-}F}$= 9 Hz), 127.2 (q, $^{2}J_{C\text{-}F}$ = 19 Hz), 126.5 (q, $^{1}J_{C\text{-}F}$ = 273 Hz), 57.8. FT-IR (film): 3083, 2110, 1702, 1583, 1310, 1271, 1165, 1110, 1033, 765 cm^{-1}. HRMS calcd for $C_9H_6F_3N_3ONa$ $[M+Na]^+$: 252.0355. Found: 252.0354.

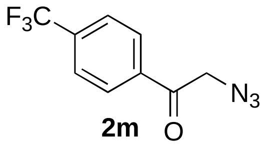

2m

2-Azido-1-[4-(trifluoromethyl)phenyl]ethanone (**2m**) [2]. Orange solid (yield: 93%). ^{1}H NMR (600 MHz, $CDCl_3$) δ 8.02 (d, *J* = 8.1 Hz, 2 H), 7.77 (d, *J* = 8.1 Hz, 2 H), 4.59 (s, 2 H). ^{13}C NMR (150 MHz, $CDCl_3$) δ 192.5, 137.0, 135.4 (q, $^{2}J_{C\text{-}F}$ = 33 Hz), 128.3, 126.0 (q, $^{3}J_{C\text{-}F}$ = 4 Hz), 124.3 (q, $^{1}J_{C\text{-}F}$ = 271 Hz), 55.1. FT-IR (KBr): 2918, 2108, 1704, 1620, 1585, 1514, 1412, 1325, 1218, 1169, 1130, 1068, 1016, 1005, 915, 846, 775, 700 cm^{-1}. GC-MS (70 eV) *m/z* (%): 201 [$(M\text{-}28)^+$, 1], 174 (11), 173 (100), 145 (63), 125 (7), 95 (9), 75 (8), 50 (4). HRMS calcd for $C_9H_6F_3N_3O$ $[M\text{-}H]^-$: 228.0390. Found: 228.0300.

2.4 Spectroscopic data of 2-aroyl-4-aryl-1*H*-imidazoles 3a–3k and 2-aroyl-5-aryl-1*H*-imidazoles 3a'–3c', 3f', 3g', 3i', and 3k'

2-Benzoyl-4-phenyl-1*H*-imidazole (3a) and 2-benzoyl-5-phenyl-1*H*-imidazole (3a') [6]. Light yellow solid (yield: 88%); **3a**:**3a' =** 57:43. ^{1}H NMR (600 MHz, $CDCl_3$) δ 10.98–10.85 (bs, 1 H, minor), 10.78–10.65 (bs, 1 H, major), 8.78 (d, *J* = 7.5 Hz, 2 H, major), 8.60 (d, *J* = 7.6 Hz, 2 H, minor), 8.35–8.25 (m, 1 H, minor), 7.92 (d, *J* = 7.5 Hz, 2 H, major), 7.68–7.63 (m, 2 H, both tautomers), 7.59–7.53 (m, 2 H major + 2 H imidazolic protons of both tautomers), 7.48 (t, *J* = 7.5 Hz, 2 H, major), 7.45 (t, *J* = 7.5 Hz, 2 H, minor), 7.41 (t, *J* = 7.3 Hz, 1 H, minor), 7.34 (t, *J* = 7.3 Hz, 1 H, major). ^{13}C NMR (150 MHz, $CDCl_3$) (both tautomers): δ: 181.4, 145.6, 145.1, 144.6, 135.7, 135.5, 133.5, 133.3, 131.3, 130.9, 129.3, 128.9, 128.7, 128.3, 127.7, 125.3, 115.7. FT-IR (KBr): 3409, 3272, 3060, 2919, 2888 1669, 1620, 1597, 1571, 1454, 1438, 1291, 1273, 1168, 903, 868, 763, 732, 689 cm^{-1}. GC-MS (70 eV) *m/z* (%): 248 (M^+, 93), 220 (100), 193 (6), 116 (10), 105 (53), 89 (12), 77 (59), 51 (11). HRMS calcd for $C_{16}H_{12}N_2O$ $[M-H]^-$: 247.0871. Found: 247.0892.

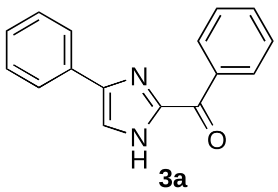

3a

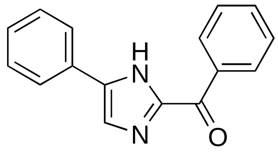

3a'

2-(4-Methylbenzoyl)-4-(*p*-tolyl)-1*H*-imidazole (3b) and 2-(4-methylbenzoyl)-5-(4-tolyl)-1*H*-imidazole (3b') [7].Yellow solid (yield: 78%); **3b**:**3b' =** 93:7. ^{1}H NMR (600 MHz, $CDCl_3$) δ 11.43–11.41 (bs, 1 H, minor), 10.83–10.80 (bs, 1 H, major), 8.65–8.55 (m, 2 H, major), 8.27 (d, *J* = 8.2 Hz, 2 H, minor), 8.01 (bs, 1 H, minor), 7.92 (d, *J* = 8.1 Hz, 2 H, minor), 7.75–7.60 (m, 2 H, major), 7.57 (s, 1 H, major), 7.35 (d, *J* = 8.0 Hz, 2 H, major), 7.33–7.30 (m, 4 H, minor), 7.26 (m, 2 H, major), 6.99 (s, 1 H, minor), 2.47 (s, 3 H, major), 2.45 (s, 3 H, minor), 2.44 (s, 3 H, minor), 2.41 (s, 3 H, major). ^{13}C NMR (150 MHz, $CDCl_3$) (both tautomers) δ 181.1, 145.3, 144.3, 133.1, 131.3, 129.7, 129.6, 129.3, 129.1, 128.1, 125.3, 21.8, 21.3. FT-IR (KBr): 3272, 3096, 3023, 2917, 2850, 1617, 1603, 1566, 1454, 1411, 1372, 1289, 1270, 1168, 905, 821, 762 cm^{-1}. GC-MS (70 eV) *m/z* (%): 276 (M^+, 100), 248 (96), 221 (3), 184 (2), 119 (53), 103 (6), 91 (45), 77 (5), 65 (13). HRMS calcd for $C_{18}H_{16}N_2O$ $[M-H]^-$: 275.1263. Found: 275.1188.

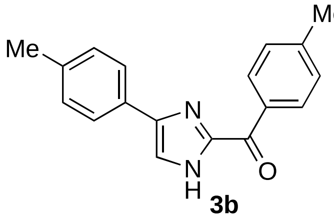

3b

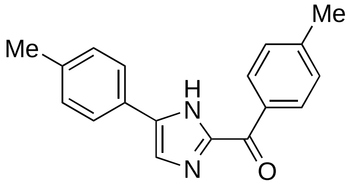

3b'

2-(4-Chlorobenzoyl)-4-(4-chlorophenyl)-1*H*-imidazole (3c) and 2-(4-chlorobenzoyl)-5-(4-chlorophenyl)-1*H*-imidazole (3c') [8]. Yellow solid (yield: 86%); **3c**:**3c' =** 88:12. ^{1}H NMR [600 MHz, $(CD_3)_2CO$] δ 12.80–12.55 (bs, 1 H major), 8.78 (d, *J* = 8.3 Hz, 2 H, major), 8.66 (d, *J* = 7.8 Hz, 2 H, minor), 8.06 (s, 1 H, major), 8.02 (d, *J* = 8.2 Hz, 2 H, major), 7.98 (m, 2 H, minor), 7.78 (s, 1 H, minor), 7.64 (d, *J* = 8.3 Hz, 2 H major), 7.61 (d, *J* = 8.4 Hz, 2 H minor), 7.52 (d, *J* = 7.9 Hz, 2 H minor), 7.46 (d, *J* = 8.3 Hz, 2 H major).^{13}C NMR [150 MHz, $(CD_3)_2CO$] (both tautomers) δ 179.3, 145.0, 142.5, 138.9, 138.8, 134.6, 132.8, 132.7, 132.5, 132.3, 129.1, 129.0, 128.6, 128.4, 128.3, 127.3, 126.6, 117.9. FT-IR (KBr): 3429, 2923, 2851, 1658, 1587, 1488, 1400, 1290, 1251, 1166, 1091, 1013, 956, 929, 832, 760 cm^{-1}. GC-MS (70 eV)

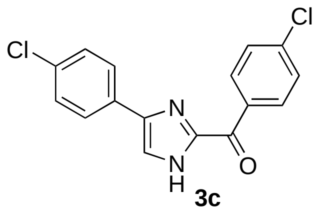

3c

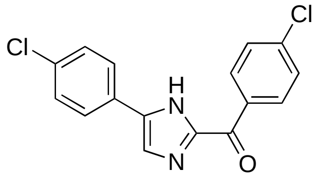

3c'

m/z (%): 316 (M^+, 98), 288 (100), 150 (11), 139 (99), 123 (13), 113 (26), 111 (82), 75 (26). HRMS calcd for $C_{16}H_{10}Cl_2N_2O$ $[M+H]^+$: 317.0170. Found: 317.0238.

2-(4-Bromobenzoyl)-4-(4-bromophenyl)-1*H*-imidazole (3d). Yellow solid (yield: 86%). ^1H NMR [600 MHz, $(CD_3)_2SO$] δ 8.50 (d, *J* = 8.0 Hz, 2 H), 8.15 (s, 1 H), 7.88 (d, 2H, *J* = 7.8 Hz), 7.82 (d, *J* = 8.0 Hz, 2 H), 7.61 (d, *J* = 7.8 Hz, 2 H). ^{13}C NMR [150 MHz,$(CD_3)_2SO$] δ 180.1, 145.0, 142.3, 135.3, 133.3, 133.1, 132.0, 131.9, 128.4, 127.9, 127.3, 125.7, 120.6, 119.9. FT-IR (KBr): 3436, 3250, 2922, 2852, 1617, 1581, 1558, 1462, 1451, 1412, 1389, 1292, 1247, 1172, 1130, 1070, 1010, 947, 905, 831, 792, 765 cm^{-1}. GC-MS (70 eV) *m/z* (%): 408 (41), 406 (M^+, 100), 404 (44), 378 (70), 327 (7), 299 (13), 195 (11), 185 (78), 183 (84), 157 (67), 155 (65), 142 (17), 115 (38), 104 (18), 89 (11), 88 (18), 76 (46), 75 (26), 63 (12), 51 (12), 50 (13). HRMS calcd for $C_{16}H_{10}Br_2N_2O$ $[M-H]^-$: 404.9061. Found: 404.9047.

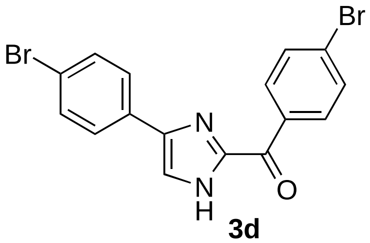

3d

2-(4-Cyanobenzoyl)-4-(4-cyanophenyl)-1*H*-imidazole (3e). Yellow waxy solid (yield: 78%). ^1H NMR (400 MHz, $CDCl_3$) δ 10.89 (bs, 1 H), 8.82 (d, 2H, *J* = 8.3 Hz), 7.98 (d, 2 H, *J* = 8.3 Hz), 7.85 (d, 2 H, *J* = 8.4 Hz), 7.73–7.71 (m, 3 H). ^{13}C NMR [100 MHz, $(CD_3)_2SO$] δ 179.8, 144.7, 141.4, 139.3, 137.9, 132.8, 132.4, 131.2, 125.4, 121.6, 119.1, 118.3, 115.1, 109.4. FT-IR (KBr): 3430, 3275, 2920, 2850, 2228, 2106, 1642, 1609, 1472, 1420, 1385, 1290, 1169, 1085, 1016, 909, 845, 771 cm^{-1}. GC-MS (70 eV) *m/z* (%): 298 (M^+, 67), 270 (100), 243 (6), 207 (3), 168 (2), 130 (69), 102 (89), 75 (23), 51 (12). HRMS calcd for $C_{18}H_9N_4O$ $[M-H]^-$: 297.0782; Found: 297.0776.

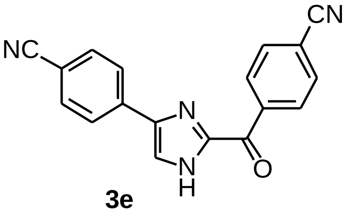

3e

2-(4-Methoxybenzoyl)-4-(4-methoxyphenyl)-1*H*-imidazole (3f) and 2-(4-methoxybenzoyl)-5-(4-methoxyphenyl)-1*H*-imidazole (3f') [9]. Yellow solid (yield: 98%); **3f**:**3f'** = 50:50. ^1H NMR (600 MHz, $CDCl_3$) (both tautomers) δ 10.95 (bs, 1 H), 10.71 (bs, 1 H), 8.92–8.60 (m, 2 H), 7.92–7.55 (m, 2 H), 7.50 (bs, 2 H), 7.04–7.02 (m, 4 H), 7.00–6.96 (m, 4 H), 3.92 (s, 6 H), 3.87 (s, 6 H). ^{13}C NMR (150 MHz, $CDCl_3$) (both tautomers) δ 180.0, 179.7, 164.0, 163.9, 160.0, 159.3, 145.5, 145.2, 144.3, 133.8, 133.4, 129.3, 128.7, 128.5, 127.8, 126.8, 126.6, 126.4, 121.2, 114.7, 114.5, 114.2, 114.1, 113.8, 113.7, 55.5, 55.3. FT-IR (KBr): 3436, 3264, 2923, 1611, 1598, 1452, 1286, 1249, 1162, 1027, 904, 833, 825 cm^{-1}. GC-MS (70 eV) *m/z* (%): 308 (M^+, 78), 280 (15), 265 (28), 200 (11), 135 (100), 92 (16), 77 (26). HRMS calcd for $C_{18}H_{16}N_2O_3$ $[M+Na]^+$: 331.1053. Found: 331.1057.

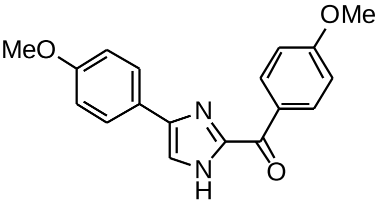

3f

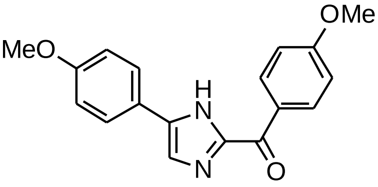

3f'

2-(2,5-Dimethoxybenzoyl)-4-(2,5-dimethoxyphenyl)-1*H*-imidazole (3g) and 2-(2,5-dimethoxybenzoyl)-5-(2,5-dimethoxyphenyl)-1*H*-imidazole (3g'). Yellow solid (yield: 94%); **3g**:**3g'** = 93:7. ^1H NMR (600 MHz, $CDCl_3$) δ 11.43 (bs, 1 H, major), 10.64 (bs, 1 H, minor), 7.70–7.60 (m, 1 H, major), 7.55–7.53 (m, 1 H, minor), 7.44–7.43 (m, 1 H, minor), 7.35–7.28 (m, 1

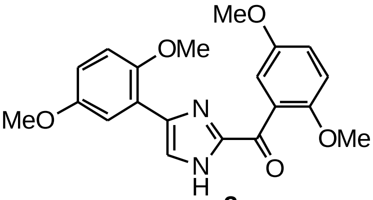

3g

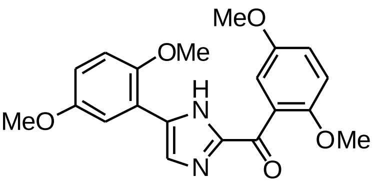

3g'

H, major), 7.27–7.23 (m, 1 H, major), 7.23–7.21 (m, 1 H, minor), 7.10–7.06 (m, 1 H, minor), 7.06–7.02 (m, 1 H, major), 7.00–6.95 (m, 2 H, major), 6.92–6.85 (m, 2 H, minor), 6.90–6.85 (m, 1 H, major), 4.03–3.95 (bs, 3 H, major), 3.87 (s, 3 H, minor), 3.83 (bs, 3 H, major), 3.81 (bs, 6 H, major), 3.77 (s, 3 H, minor), 3.60 (s, 3 H, minor), 3.50 (s, 3 H, minor). ^{13}C NMR (150 MHz, $CDCl_3$) (both tautomers) δ 183.3, 154.0, 153.1, 152.5, 150.0, 144.6, 127.2, 121.8, 120.9, 118.5, 115.8, 114.8, 113.5, 112.7, 56.7, 56.2, 55.9, 55.8. FT-IR (KBr): 3431, 2922, 1627, 1513, 1469, 1378, 1270, 1137, 1022, 853, 768, 608, 452cm^{-1}. GC-MS (70 eV) *m/z* (%): 368 (M^+, 72), 337 (100), 323 (19), 319 (10), 307 (38), 218 (12), 217 (15), 203 (15), 187 (8), 184 (8), 176 (11), 169 (11), 165 (46), 162 (19), 135 (11), 122 (11), 107 (17), 77 (11). HRMS calcd for $C_{20}H_{20}N_2O_5$ $[M+H]^+$: 369.1445. Found: 369.3906.

4-(2-Hydroxyphenyl)-2-(2-hydroxybenzoyl)-1*H*-imidazole (3h). Brown solid (yield: 71%).

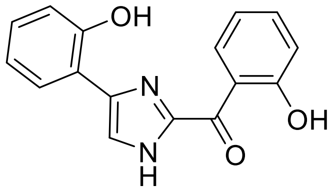

3h

^{1}H NMR (300 MHz, $CDCl_3$) δ 12.24 (bs, 1 H), 9.45 (bs, 1 H), 8.47–8.45 (m, 1 H), 8.11–8.10 (m, 1 H), 7.89–7.86 (m,1 H), 7.68–7.67 (m, 1 H), 7.55–7.51 (m, 2 H), 7.17–7.15 (m, 1 H), 7.10–7.08 (m,1 H). ^{13}C NMR [75 MHz,$(CD_3)_2SO$] δ 175.5, 161.1, 155.6, 154.7, 139.8, 136.0, 134.9, 131.3, 129.3, 127.6, 127.3, 125.5, 123.2, 120.2, 119.4, 118.3. FT-IR (KBr): 3344, 2927, 2873, 1634, 1471, 1110, 1042, 956, 924, 864, 753 cm^{-1}. HRMS calcd for $C_{16}H_{12}N_2O_3$ $[M+H]^+$: 281.0842. Found: 281.0833.

4-(4-Fluorophenyl)-2-(4-fluorobenzoyl)-1*H*-imidazole (3i) and 5-(4-fluorophenyl)-2-(4-fluorobenzoyl)-1*H*-imidazole (3i') [9,10]. Yellow solid (yield: 87%); **3i**:**3i'** = 90:10. ^{1}H NMR

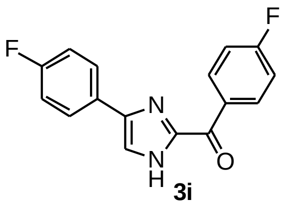

3i

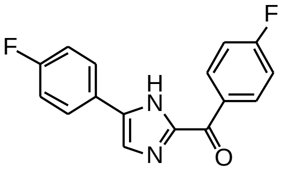

3i'

[600 MHz, $(CD_3)_2CO$] δ 8.90–8.87 (m, 2 H, major), 8.78–8.73 (m, 2 H, minor), 8.04–8.01 (m, 2 H, both tautomers), 7.98 (s, 1 H), 7.72 (s, 1 H, minor), 7.37–7.30 (m, 2 H, both tautomers), 7.29–7.25 (m, 2 H, minor), 7.22–7.19 (m, 2 H, major). ^{13}C NMR [150 MHz,$(CD_3)_2CO$] (both tautomers) δ 180.6, 167.4 (d, $^{1}J_{C\text{-}F}$ = 253 Hz), 163.8 (d, $^{1}J_{C\text{-}F}$ = 244 Hz), 146.7, 144.5, 135.6 (d, $^{3}J_{C\text{-}F}$ = 9 Hz), 134.3, 132.1, 128.6 (d, $^{3}J_{C\text{-}F}$ = 8 Hz), 118.8, 116.9 (d, $^{2}J_{C\text{-}F}$ = 22 Hz), 116.8 (d, $^{2}J_{C\text{-}F}$ = 22 Hz). FT-IR (neat) 3435, 3276, 3128, 3096, 2922, 1618, 1598, 1583, 1565, 1510, 1452, 1291, 1241, 1170, 1160, 1101, 907, 842, 775 cm^{-1}. GC-MS (70 eV) *m/z* (%): 284 (M^+, 100), 256 (87), 229 (5), 201 (2), 189 (1), 160 (3), 142 (2), 134 (10), 123 (79), 107 (13), 95 (53), 81 (2), 75 (12), 57 (3). HRMS calcd for $C_{16}H_{10}N_2OF_2$ $[M+Na]^+$: 307.0653. Found: 307.0653.

4-(2-Naphtyl)-2-(2-naphtyloyl)-1*H*-imidazole (3j) [10]. Light brown solid (yield: 67%).^{1}H NMR [600 MHz, $(CD_3)_2SO$] δ 9.50–9.40 (bs, 1 H), 8.57–8.46 (m, 2 H), 8.30–8.20 (m, 2 H),

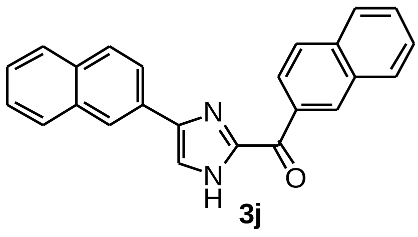

3j

8.17–8.09 (m, 3 H), 8.05 (d, *J* = 8.0 Hz, 1 H), 8.01–7.95 (m, 3 H), 7.92 (d, *J* = 7.8 Hz, 1 H), 7.73–7.70 (m, 1 H), 7.69–7.64 (m, 1 H), 7.56–7.46 (m, 2 H). ^{13}C NMR [150 MHz, $(CD_3)_2SO$] δ 180.7, 145.2, 135.0, 133.3, 133.0, 132.4, 132.0, 130.0, 129.4, 128.8, 128.3, 127.9, 127.7, 127.3, 126.9, 126.5, 125.9, 123.8, 123.0, 119.3. FT-IR (KBr): 3279, 3053, 2921, 2851, 1632, 1611, 1480, 1447, 1360, 1279, 1269, 1230, 1160, 1125, 925, 860, 823, 806, 781, 738 cm^{-1}. HRMS calcd for $C_{24}H_{16}N_2O$ $[M-H]^-$: 347.1184. Found: 347.1177.

4-[1,1'-Biphenyl-4-yl]-2-(4-phenybenzoyl)-1*H*-imidazole (3k) and 5-[1,1'-biphenyl-4-yl]-2-(4-phenybenzoyl)-1*H*-imidazole (3k') [10]. Brown solid (yield: 32%); **3k**:**3k' =** 79:21. ^{1}H NMR [600 MHz, $(CD_3)_2SO$] δ 10.21 (bs, 1 H, minor), 8.71 (d, *J* = 8.2 Hz, 2 H, major), 8.59 (d, *J* = 7.2 Hz, 2 H, minor), 8.15 (s, 1 H), 8.08–8.05 (m, 2 H, minor), 8.03 (d, *J* = 8.1 Hz, 2 H, major), 8.03–7.90 (m, 2 H, minor), 7.93 (d, *J* = 8.2 Hz, 2 H, major), 7.90-7.87 (m, 2 H, minor), 7.82 (d, *J* = 7.6 Hz, 2 H, major), 7.80-7.76 (m, 2 H, minor), 7.74 (d, *J* = 8.2 Hz, 2 H, major), 7.72 (d, *J* = 7.8 Hz, 2 H, major), 7.53 (t, *J* = 7.6 Hz, 2 H, major), 7.50–7.43 (m, 3 H, major + minor), 7.37 (t, *J* = 7.3 Hz, 1 H, major). ^{13}C NMR [150 MHz, $(CD_3)_2SO$] δ 180.6, 145.3, 144.9, 143.0, 140.3, 139.5, 139.3, 135.2, 133.3, 131.9, 129.6, 129.4, 128.9, 127.8, 127.6, 127.5, 127.4, 127.0, 126.9, 125.9, 119.3. FT-IR (KBr): 3421, 3271, 3055, 3031, 2923, 2852, 1670, 1615, 1600, 1485, 1458, 1404, 1294, 1275, 1170, 1115, 1076, 1006, 907, 840, 749, 723, 695 cm^{-1}. HRMS calcd for $C_{28}H_{20}N_2O$ $[M-H]^-$: 399.1503; Found: 399.1489.

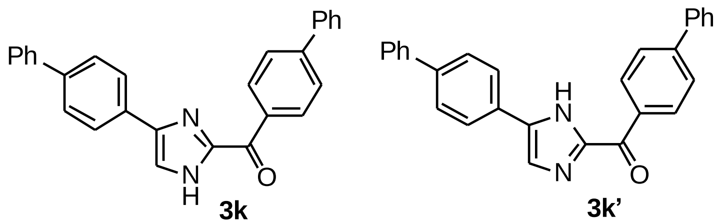

3k **3k'**

Spectroscopic data of 2,4-diaroyl-6-arylpyrimidines 7a–h

2,4-Dibenzoyl-6-phenylpyrimidine (**7a**). Yellow waxy solid (yield: 57%). ^{1}H NMR (600 MHz, $CDCl_3$) δ 9.21 (s, 1 H), 8.14–8.12 (m, 2 H), 8.06–8.04 (m, 3 H), 7.66–7.63 (m, 2 H), 7.56–7.50 (m, 8 H). ^{13}C NMR (150 MHz, $CDCl_3$) δ 192.4, 192.2, 152.9, 151.4, 149.8, 140.0, 133.8, 133.7, 131.1, 130.7, 130.6, 129.3, 128.5, 128.4, 127.5. FT-IR (KBr): 3059, 3033, 2922, 2851, 1674, 1663, 1596, 1581, 1546, 1450, 1319, 1290, 1249, 1168, 1121, 1067, 957, 938, 766, 717, 704, 693 cm^{-1}. GC-MS (70 eV) *m/z* (%): 364 (M^+, 50), 336 (4), 308 (3), 287 (9), 259 (26), 232 (3), 204 (1), 128 (3), 105 (100), 77 (59), 51 (7). HRMS calcd for $C_{24}H_{16}N_2O_2$ $[M+Na]^+$: 387.1109. Found: 387.1117.

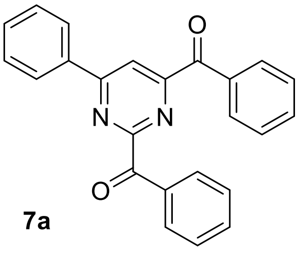

7a

2,4-Bis(4-methylbenzoyl)-6-tolylpyrimidine (**7b**). Light yellow oil (yield: 52%). ^{1}H NMR (600 MHz, $CDCl_3$) δ 9.15 (s, 1 H), 8.03 (d, *J* = 8.1 Hz, 2 H), 7.95–7.92 (m, 4 H), 7.34 (d, *J* = 8.1 Hz, 2 H), 7.31–7.28 (m, 4 H), 2.45–2.44 (m, 9 H). ^{13}C NMR (150 MHz, $CDCl_3$) δ 192.1, 191.9, 153.0, 151.3, 149.5, 144.7, 144.6, 141.5, 139.6, 133.0, 132.9, 132.1, 130.8, 130.7, 130.0, 129.2, 129.1, 127.4, 21.8, 21.5. FT-IR (neat): 3032, 2918, 2850, 1660, 1606, 1548, 1519, 1447, 1410, 1315, 1296, 1256, 1181, 1119, 1068, 957, 931, 825, 773, 751, 737 cm^{-1}. GC-MS (70 eV) *m/z* (%): 406 (M^+, 44), 391 (1), 378 (3), 377 (2), 350 (3), 287 (25), 260 (2), 203 (2), 142 (2), 119 (100), 91 (42), 65 (10), 51 (7). HRMS calcd for $C_{27}H_{22}N_2O_2$ $[M+Na]^+$: 429.1579. Found: 429.1592.

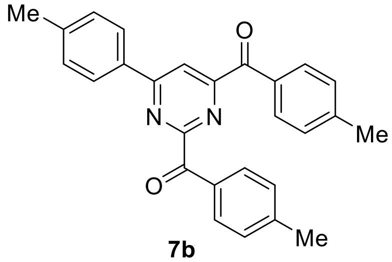

7b

2,4-Bis(4-chlorobenzoyl)-6-(4-chlorophenyl)pyrimidine (**7c**). Light brown solid (yield: 45%). ^{1}H NMR (600 MHz, $CDCl_3$) δ 9.15 (s, 1 H), 8.04 (d, *J* = 8.6 Hz, 2 H), 7.99 (d, *J* = 8.6 Hz, 2 H), 7.94 (d, *J* = 8.6 Hz, 2 H), 7.51 (d, *J* = 8.6 Hz, 2 H), 7.48–7.46 (m, 4 H). ^{13}C NMR (150 MHz, $CDCl_3$) δ 190.9, 190.6, 152.6, 150.5, 149.6, 140.6, 140.5, 139.8, 137.8, 133.5, 133.5, 132.9, 132.1, 131.9, 129.7, 129.0, 128.9, 128.7. FT-IR (KBr): 2918, 2850, 1660, 1588, 1401, 1092, 833 cm^{-1}. GC-MS (70 eV) *m/z* (%): 470 $[M^{37}(Cl)_2{}^{35}(Cl)^+$, 3], 468 $[M^{37}(Cl)^{35}(Cl)_2{}^+$, 10], 466 [M $^{35}(Cl)_3{}^+$, 10], 355 (1), 329 (4), 327 (6), 142 (2), 141 (33), 139 (100), 136 (2), 113 (12), 111 (38), 75 (10). HRMS calcd for $C_{24}H_{12}Cl_3N_2O_2$ [M $^{35}(Cl)_3$-H$]^-$: 464.9970. Found: 464.9964 [M $^{35}(Cl)_3$-H$]^-$, 466.9580 [M $^{35}(Cl)_2{}^{37}(Cl)$-H$]^-$, 468.9550 [M $^{35}(Cl)^{37}(Cl)_2$-H$]^-$.

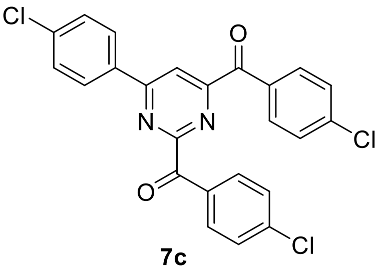

7c

2,4-Bis(4-bromobenzoyl)-6-(4-bromophenyl)pyrimidine (**7d**). Brown solid (yield: 82%). ^{1}H NMR (600 MHz, $CDCl_3$) δ 9.18 (s, 1 H), 7.99 (d, *J* = 8.4 Hz, 2 H), 7.93 (d, *J* = 8.4 Hz, 2 H), 7.88 (d, *J* = 8.4 Hz, 2 H), 7.70 (d, *J* = 8.4 Hz, 2 H), 7.68–7.66 (m, 4 H). ^{13}C NMR (150 MHz, $CDCl_3$) δ 191.1, 190.8, 152.6, 150.6, 149.6, 139.8, 133.9, 133.4, 132.7, 132.2, 132.0, 131.9, 131.8. FT-IR (KBr): 2918, 2849, 1660, 1584, 1483, 1396, 1290, 1167, 1070, 1009, 955, 928, 829, 756 cm^{-1}. HRMS calcd for $C_{24}H_{14}N_2Br_3O_2$ $[M+H]^+$: 598.8605. Found: 598.8460.

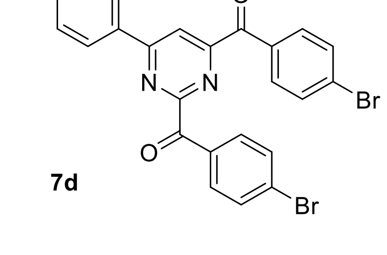

7d

2,4-Bis-(2-hydroxybenzoyl)-6-(2-hydroxyphenyl)pyrimidine (**7e**). Yellow pale solid (yield: 75%). ^{1}H NMR (600 MHz, $CDCl_3$) δ 12.24 (bs, 1 H), 11.37 (bs, 2 H), 9.46 (s, 1 H), 8.47–8.45 (m, 1 H), 8.11–8.10 (m, 1 H), 7.89–7.86 (m, 1 H), 7.68–7.67 (m, 1 H), 7.55–7.51 (m, 2 H), 7.17–7.15 (m, 1 H), 7.10–7.07 (m, 1 H), 6.89–6.88 (m, 2 H), 6.80–6.79 (m, 2 H). ^{13}C NMR (150 MHz, $CDCl_3$) δ 175.6, 161.2, 155.6, 154.8, 154.7, 139.8, 136.1, 135.0, 132.8, 131.3, 130.2, 129.3, 127.7, 127.4, 125.6, 123.2, 122.3, 120.9, 120.2, 119.5, 118.4, 117.2, 115.9, 115.6. FT IR (KBr): 3341, 2918, 2850, 1614, 1469, 1157, 1038, 886, 754 cm^{-1}. HRMS calcd for $C_{24}H_{16}N_2O_5$ $[M+H]^+$: 413.1132. Found: 413.1126.

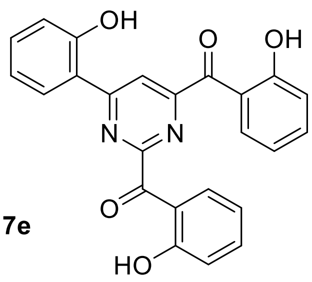

7e

2,4-Bis(4-fluorobenzoyl)-6-(4-fluorophenyl)pyrimidine (**7f**). Orange solid (yield: 61%). ^{1}H NMR (600 MHz, $CDCl_3$) δ 9.15 (s, 1 H), 8.12–8.10 (m, 4 H), 8.07–8.05 (m, 2 H), 7.24–7.20 (m, 3 H), 7.19–7.17 (m, 3 H). ^{13}C NMR (150 MHz, $CDCl_3$) δ 190.7, 190.4, 166.3 (d, $^1J_{C\text{-}F}$ = 257 Hz), 164.8 (d, $^1J_{C\text{-}F}$ = 253 Hz), 152.8, 150.5, 149.5, 139.6, 133.6 (d, $^3J_{C\text{-}F}$= 9 Hz), 133.3 (d, $^3J_{C\text{-}F}$ = 9 Hz), 131.6, 130.8 (m), 130.7 (d, $^3J_{C\text{-}F}$ = 9 Hz), 129.6 (d, $^3J_{C\text{-}F}$ = 9 Hz), 116.6 (d, $^2J_{C\text{-}F}$ = 22 Hz), 116.2 (d, $^2J_{C\text{-}F}$ = 22 Hz), 115.9 (d, $^2J_{C\text{-}F}$ = 17 Hz), 115.7 (d, $^2J_{C\text{-}F}$ = 17 Hz). FT-IR (KBr): 2919, 2805, 1668, 1598, 1506, 1411, 1294, 1236, 1156, 1120, 1067, 958, 932, 849, 785, 761 cm^{-1}. GC-MS (70 eV) *m/z* (%): 418 (M^+, 25), 323 (2), 295 (9), 146 (2), 124 (7), 123 (100), 120 (2), 95 (36), 75 (6), 77 (46). HRMS calcd for $C_{24}H_{12}F_3N_2O_2$ $[M-H]^-$: 417.0856. Found: 417.0849.

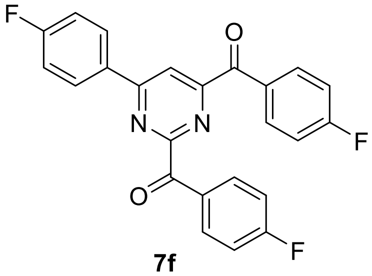

7f

2,4-Bis[2-(trifluoromethyl)benzoyl]-6-[2-(trifluoromethyl)phenyl]pyrimidine (**7g**). Yellow solid (yield: 88%). ^{1}H NMR (600 MHz, $CDCl_3$) δ 8.81 (s, 1 H), 7.90–7.53 (m, 12 H). ^{13}C NMR (150 MHz, $CDCl_3$) δ 204.4, 183.0, 143.9, 135.7, 135.3, 134.5, 132.0, 131.7, 131.6, 131.5, 131.2, 130.6, 130.0, 127.1, 127.0, 122.8, 122.7, 122.2, 122.1, 119.8. FT-IR (KBr): 2919, 2851, 1693, 1583, 1449, 1365, 1320, 1141, 1061, 1036, 961, 934, 872, 768 cm^{-1}. GC-MS(70 eV) *m/z* (%): 568 (M^+, 5), 499 (9), 395 (2), 375 (3), 173 (100), 145 (42), 125 (2), 95 (2). HRMS calcd for $C_{27}H_{13}N_2F_9O_2$ $[M+Na]^+$: 591.0726. Found: 591.0731.

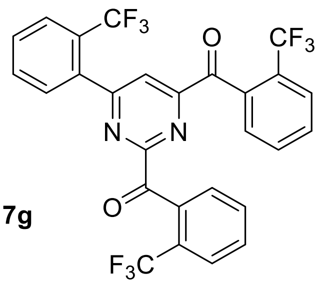

7g

2,4-Bis[4-(trifluoromethyl]benzoyl)-[6-(4-trifluoromethyl)phenyl]pyrimidine (**7h**). Brown solid (yield: 69%). ^{1}H NMR (600 MHz, $CDCl_3$) δ 9.29 (s, 1 H), 8.25 (d, *J* = 8.2 Hz, 2 H), 8.19 (d, *J* = 8.2 Hz, 2 H), 8.16 (d, *J* = 8.1 Hz, 2 H), 7.81–7.80 (m, 6 H). ^{13}C NMR (150 MHz, $CDCl_3$) δ 190.9, 190.8, 152.5, 150.5, 150.0, 140.7, 137.8, 137.7, 137.6, 135.3, 131.0, 130.8, 127.9, 126.4, 125.7, 125.6, 125.4, 121.9, 120.8. FT IR (KBr): 2918, 2850, 1661, 1411, 1325, 1127, 842 cm^{-1}. GC-MS (70 eV) *m/z* (%): 568 (M^+, 20), 549 (4), 423 (2), 395 (7), 196 (1), 173 (100), 145 (44), 95 (3), 75 (1). HRMS calcd for $C_{27}H_{13}N_2O_2F_9$ $[M+Na]^+$: 591.0726. Found: 591.0729.

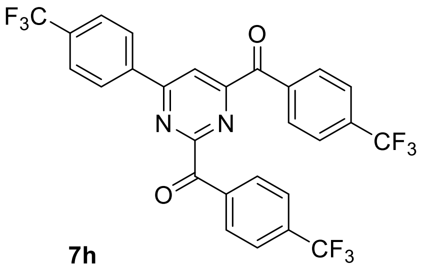

7h

1.2 References

[1] Vitale, P.; Cicco, L.; Messa, F.; Perna, F. M.; Salomone, A.; Capriati, V. *Eur. J. Org. Chem.*, **2019**, 5557–5562.

[2] Wei, W.; Cui, H.; Yue, H.; Yang, D. *Green Chem*., **2018**, *20*, 3197–3202.

[3] Hossain, A.; Vidyasagar, A.; Eichinger, C.; Lankes, C.; Phan, J.; Rehbein, J.; Reiser, O. *Angew. Chem. Int. Ed.* **2018,** *57*, 8288–8292.

[4] Prasad, B.; Phanindrudu, M.; Tiwari, D. K.; Kamal, A. *J. Org. Chem.* **2019**, *84*, 12334–12343.

[5] Moumné, R.; Larue, V.; Lecourt, T.; Micouin, L.; Tisné, C. *Org. Biom. Chem.* **2010**, *8*, 1154–1159.

[6] a) Chen, J.; Chen, W.; Yu ,Y.; Zhang, G. *Tetrahedron Lett.* **2013**, *54*, 1572–1575. b) Liu, C.; Dai, R.J.; Yao, G.W.; Deng, Y.L. *ARKIVOC* **2014** (iv) 146–163.

[7] Xiao, M.; Ahn, S.; Wang, J.; Chen, J.; Miller, D. D.; Dalton, J. T.; Li, W. *J. Med. Chem.* **2013**, *56*, 3318−3329.

[8] Khalili, B.; Tondro, T. ; Hashemi, M.M. *Tetrahedron*, **2009**, *65*, 6882–6887.

[9] Kong, Y. C.; Kim, K. *J. Heterocycl. Chem.***1999**, *36*, 911.

[10] Kuzu, B.; Tan, M.; Taslimi ,P.; Gülçin, İ.; Taşpınar, M.; Menges, N. *Bioorg. Chem.***2019**, *86*, 187–196.

2. NMR spectra

2.1 NMR spectra of 2-azido ketones 2d, 2e, 2f, 2k, 2l, and 2m

^{1}H and ^{13}C NMR spectra of **2-azido-1-(4-bromophenyl)ethanone** (**2d**)

^{1}H NMR (600 MHz, $CDCl_3$)

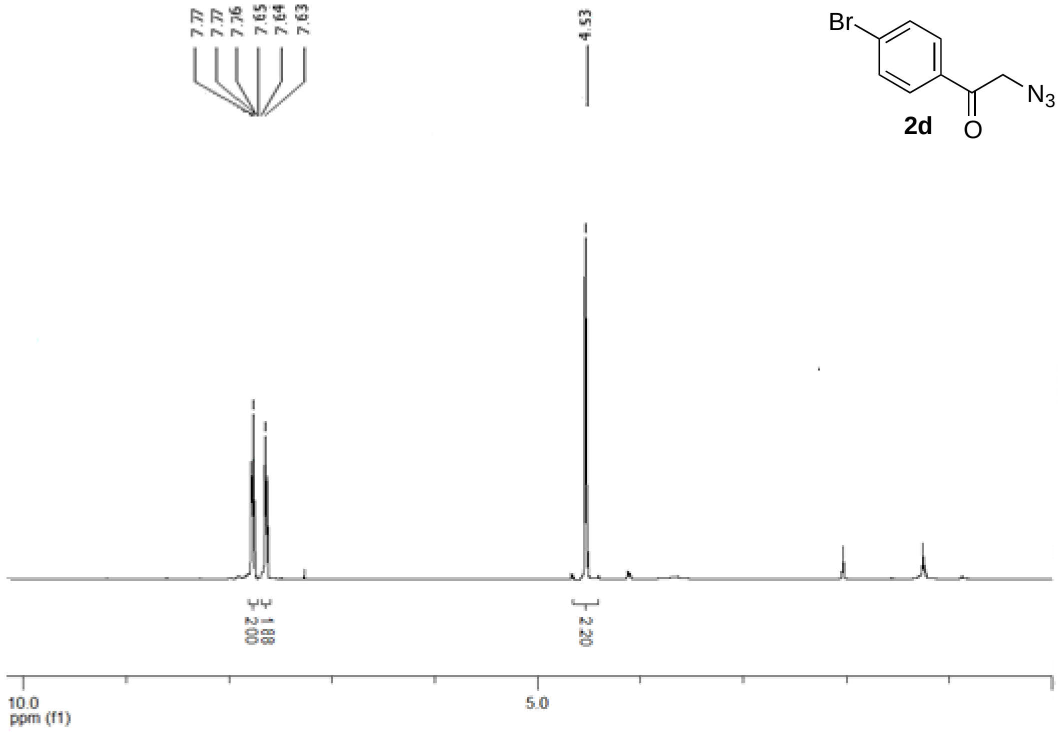

^{13}C NMR (150 MHz, $CDCl_3$)

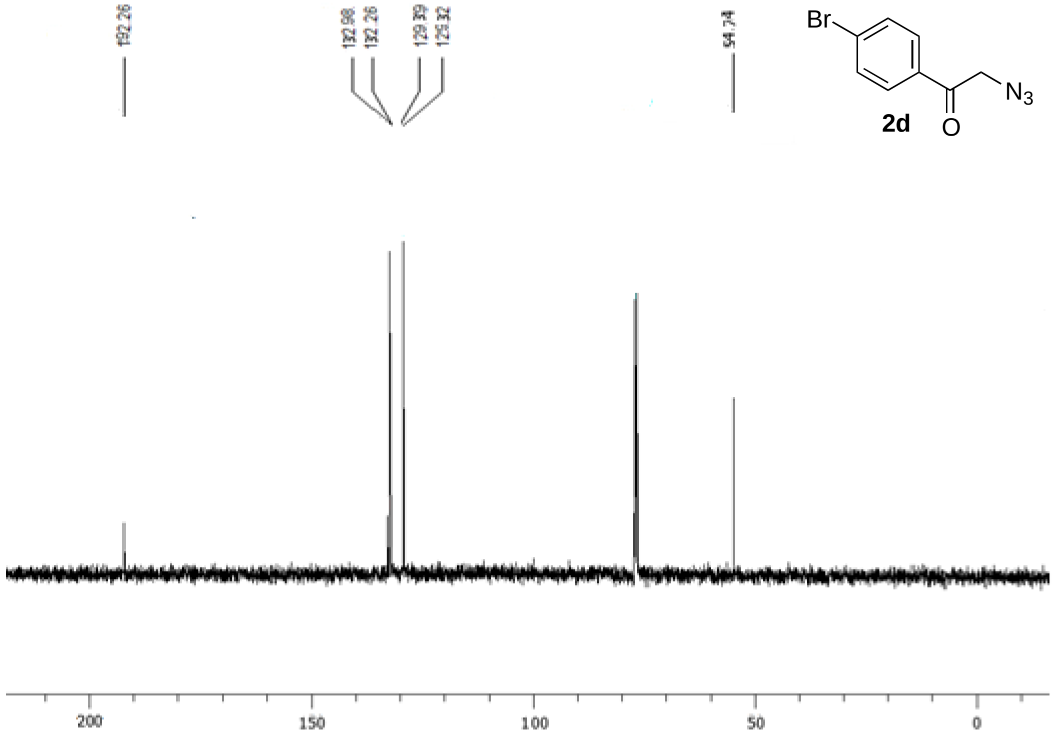

^{1}H and ^{13}C NMR spectra of **4-(2-azidoacetyl)benzonitrile** (**2e**)

^{1}H NMR (600 MHz, $CDCl_3$)

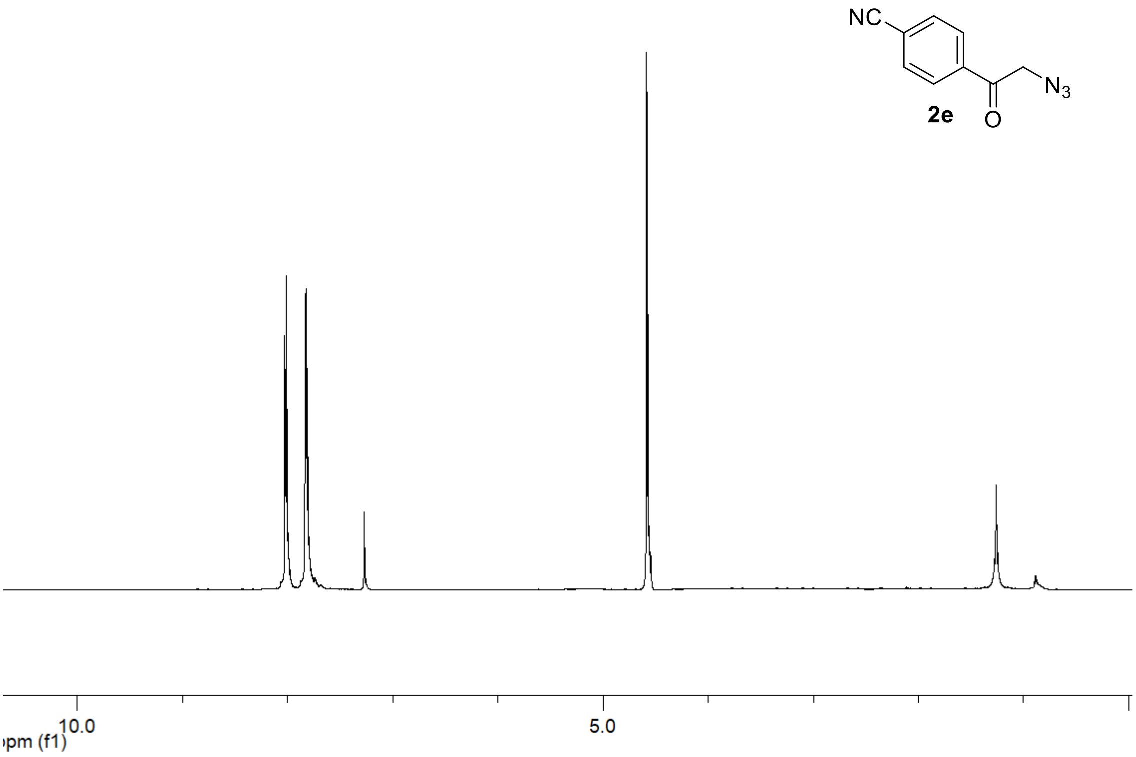

^{13}C NMR (150 MHz, $CDCl_3$)

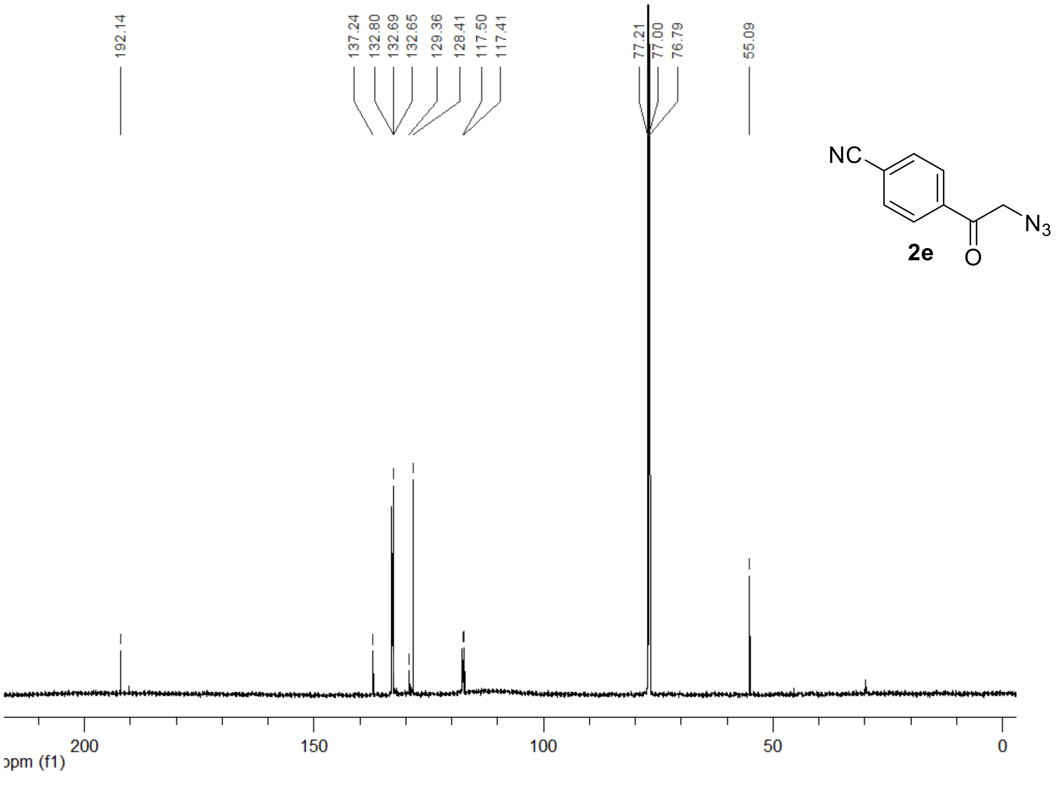

^{1}H and ^{13}C NMR spectra of **2-azido-1-(4-methoxyphenyl)ethanone** (**2f**)

^{1}H NMR (600 MHz, $CDCl_3$)

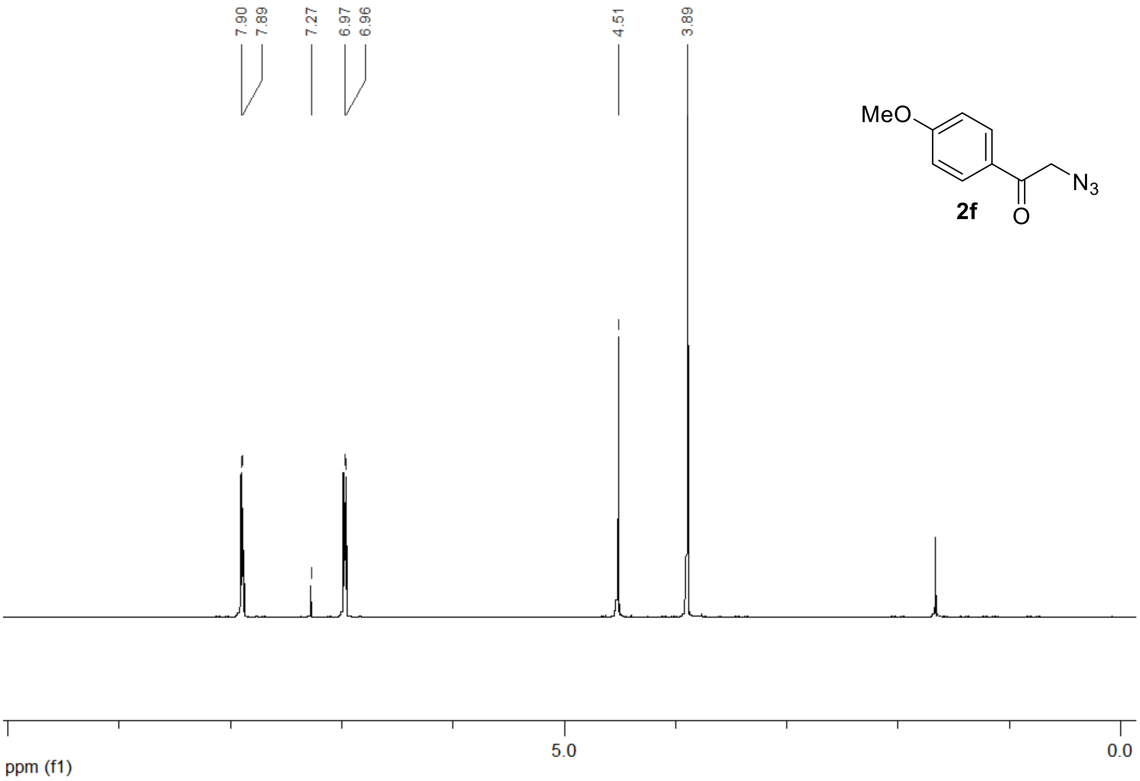

^{13}C NMR (150 MHz, $CDCl_3$)

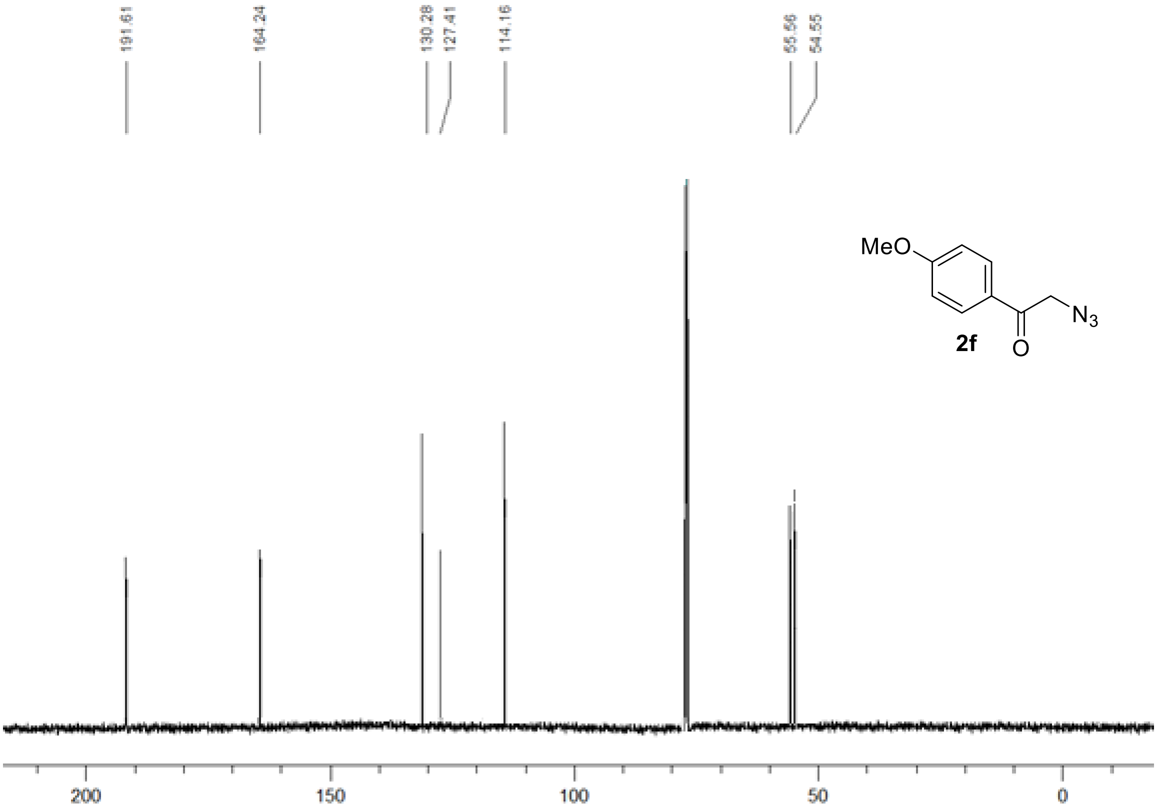

^{1}H and ^{13}C NMR spectra of **2-azido-1-([1,1'-biphenyl]-4-yl)ethanone** (**2k**)

^{1}H NMR (600 MHz, $CDCl_3$)

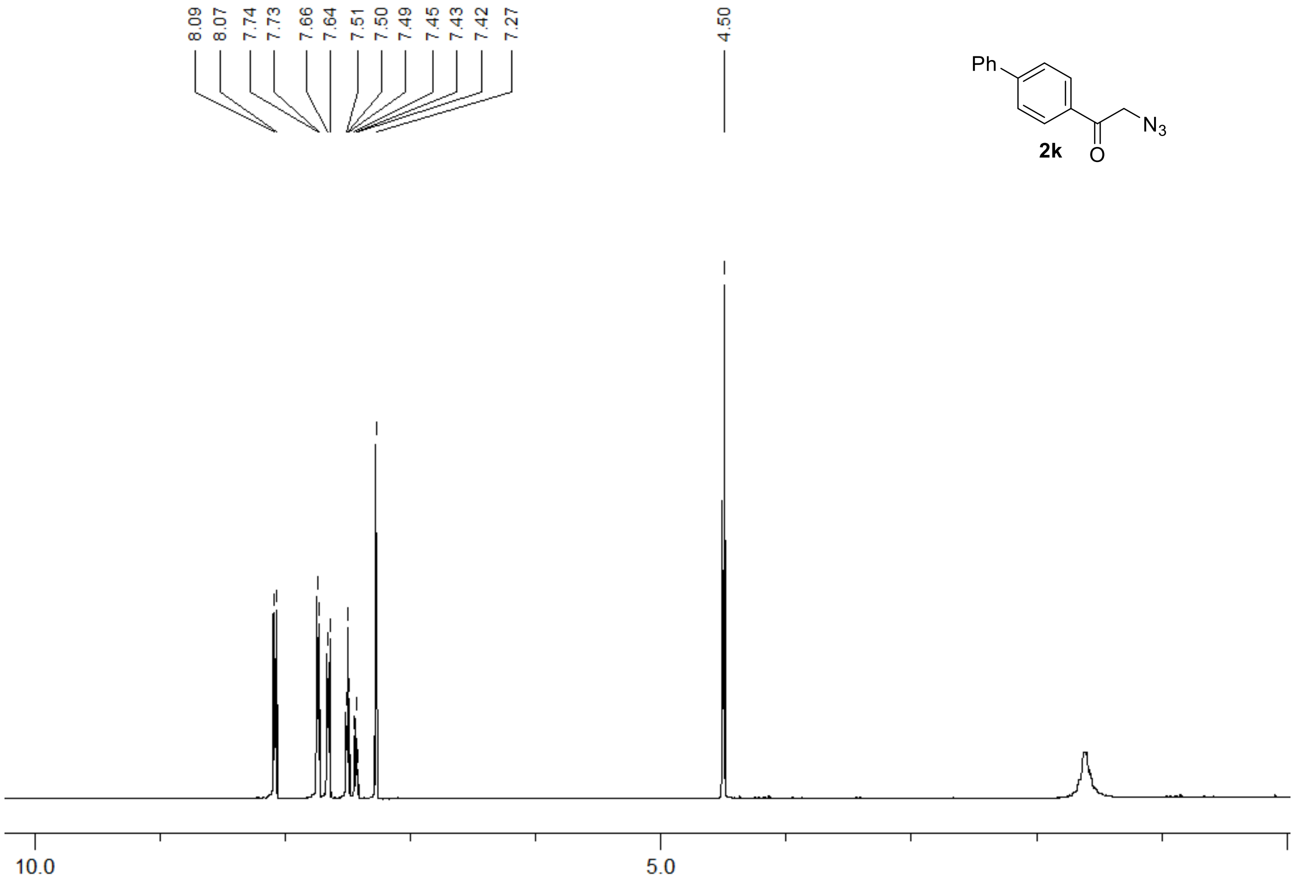

^{13}C NMR (150 MHz, $CDCl_3$)

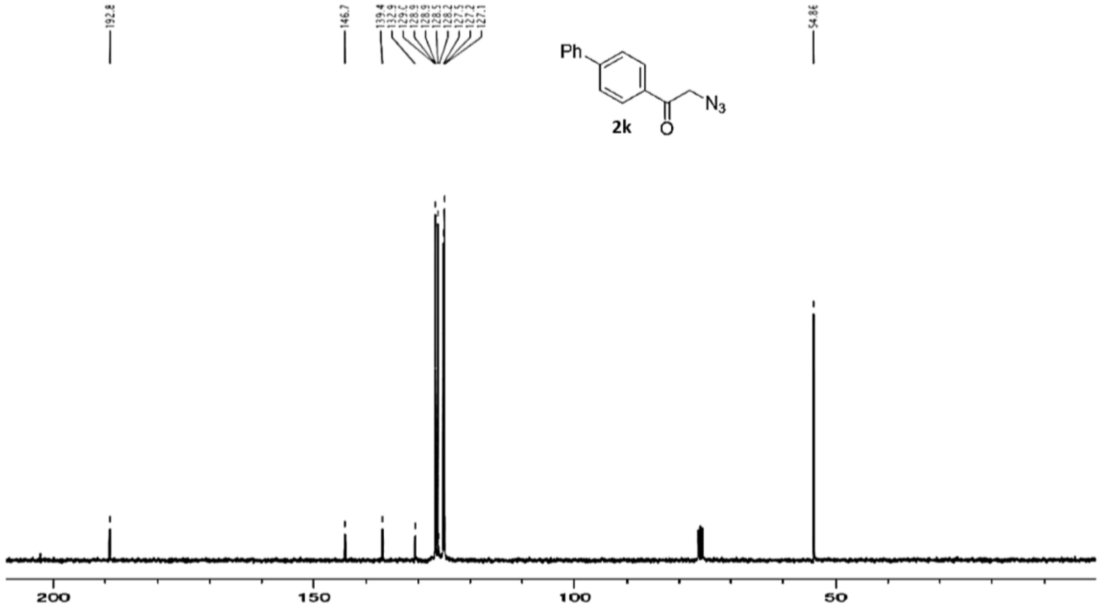

^{1}H and ^{13}C NMR spectra of **2-azido-1-[2-(trifluoromethyl)phenyl]ethanone** (**2l**)

^{1}H NMR (600 MHz, $CDCl_3$)

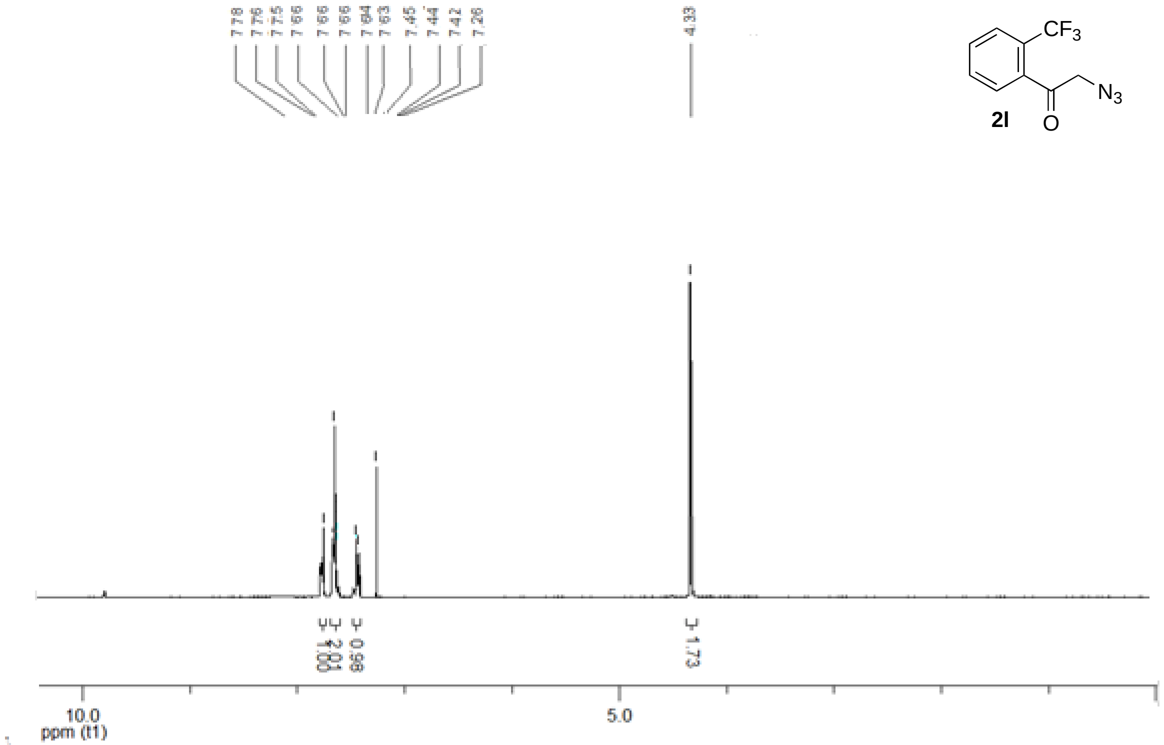

^{13}C NMR (150 MHz, $CDCl_3$)

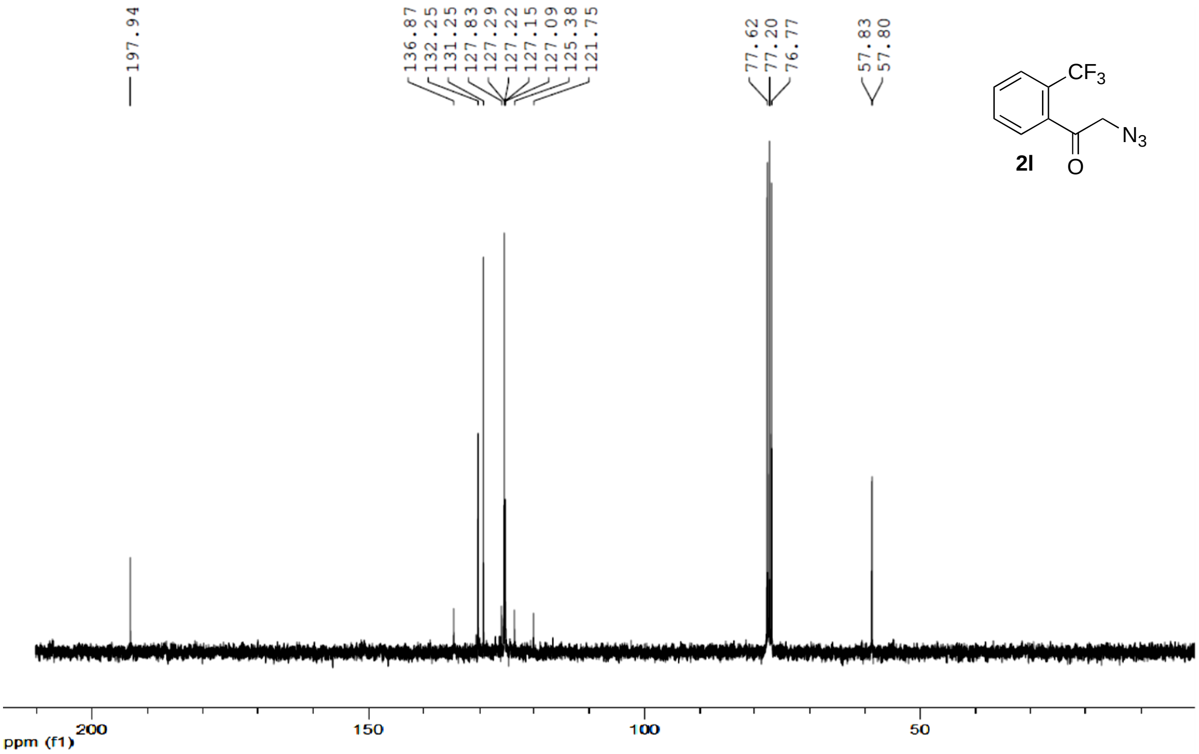

^{1}H and ^{13}C NMR spectra of **2-azido-1-[4-(trifluoromethyl)phenyl]ethanone** (**2m**)

^{1}H NMR (600 MHz, $CDCl_3$)

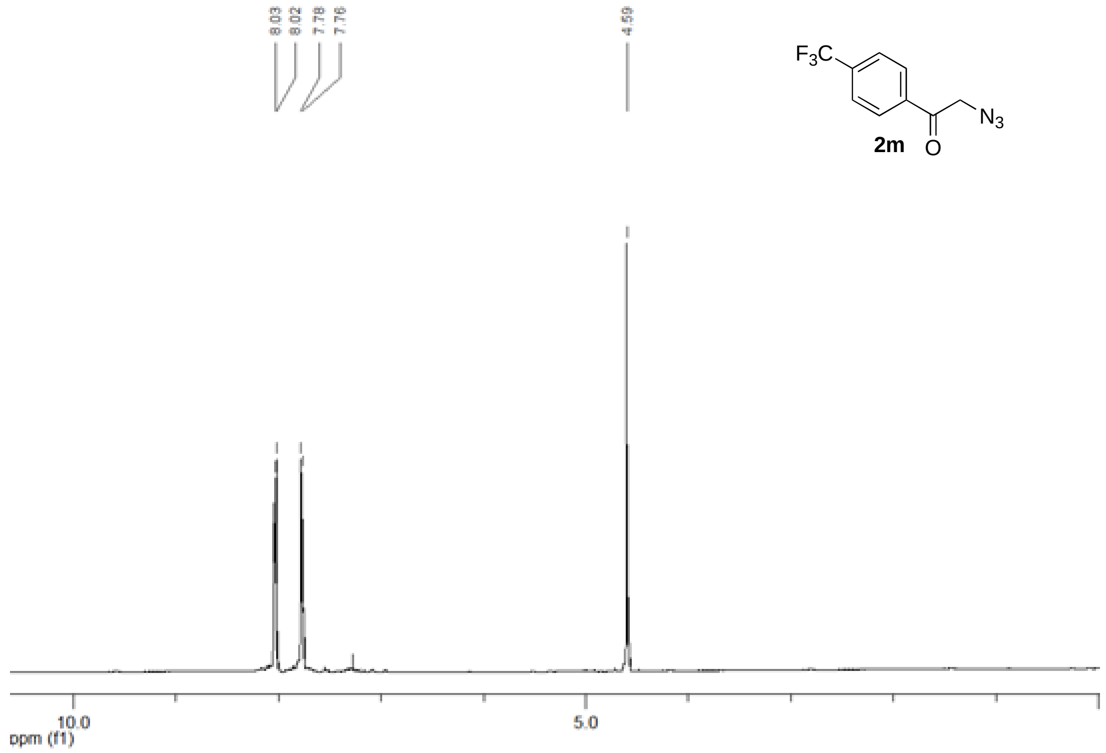

^{13}C NMR (150 MHz, $CDCl_3$)

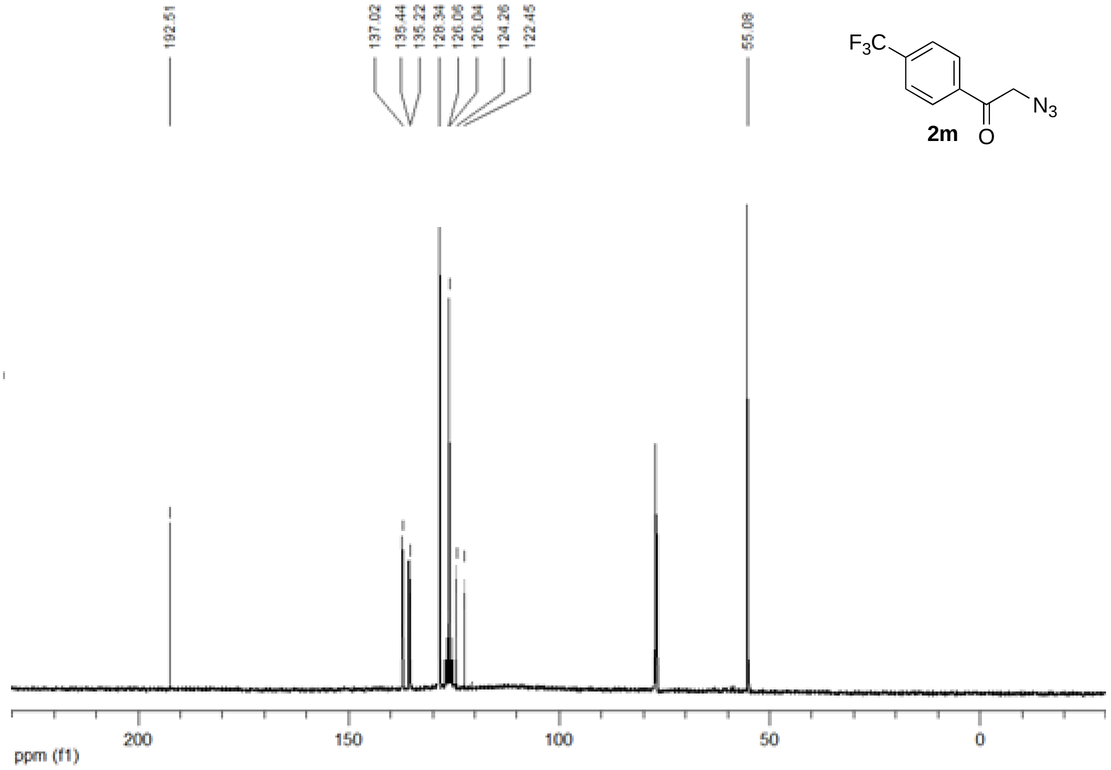

2.2 NMR spectra of 2-aroyl-4-aryl-1*H*-imidazoles 3a–3k and 2-aroyl-5-aryl-1*H*-imidazoles 3a'–3c', 3f', 3g', 3i', and 3k'

^{1}H and ^{13}C NMR spectra of **2-benzoyl-4-phenyl-1*H*-imidazole** (**3a**) and **2-benzoyl-5-phenyl-1*H*-imidazole** (**3a'**)

^{1}H NMR (600 MHz, $CDCl_3$)

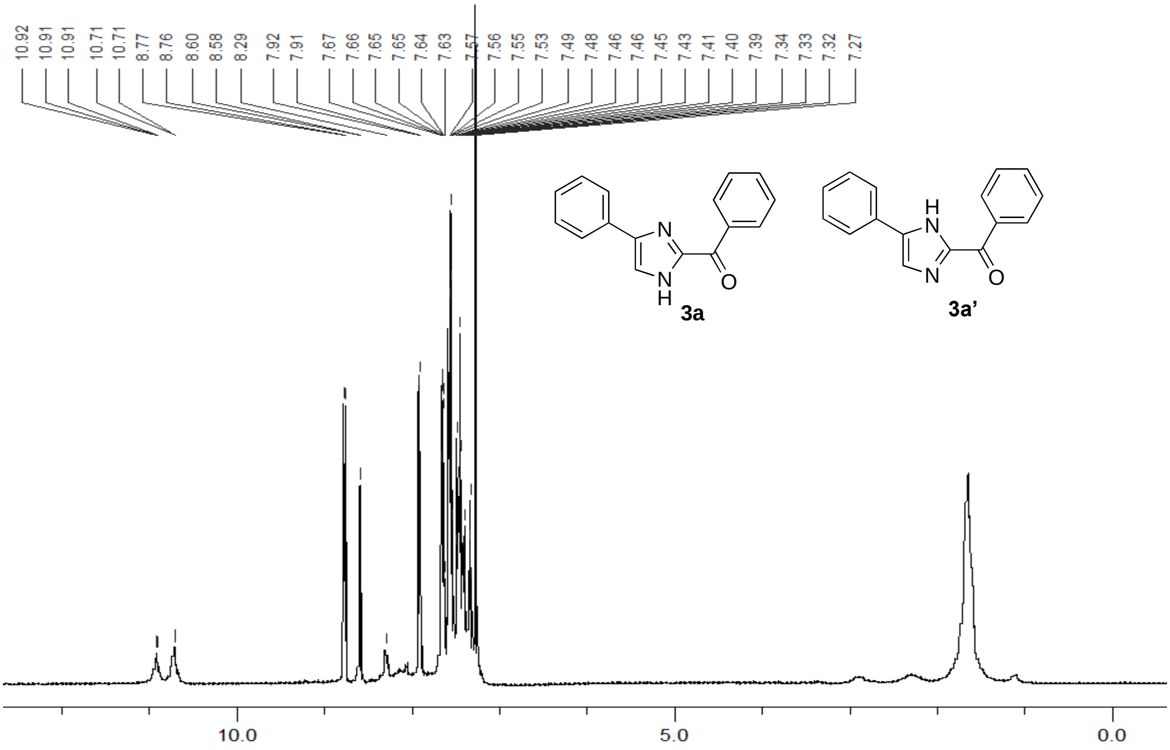

^{13}C NMR (150 MHz, $CDCl_3$)

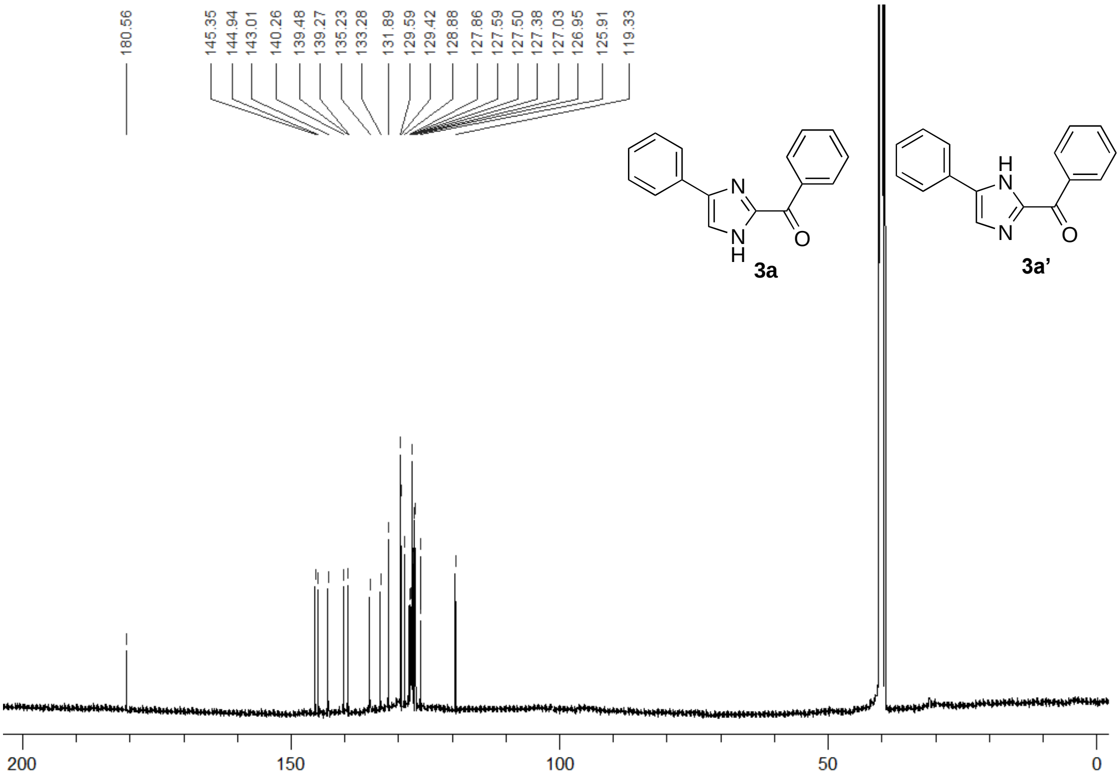

^{1}H and ^{13}C NMR spectra of **2-(4-methylbenzoyl)-4-(*p*-tolyl)-1*H*-imidazole** (**3b**) and **2-(4-methylbenzoyl)-5-(*p*-tolyl)-1*H*-imidazole** (**3b'**)

^{1}H NMR (600 MHz, $CDCl_3$)

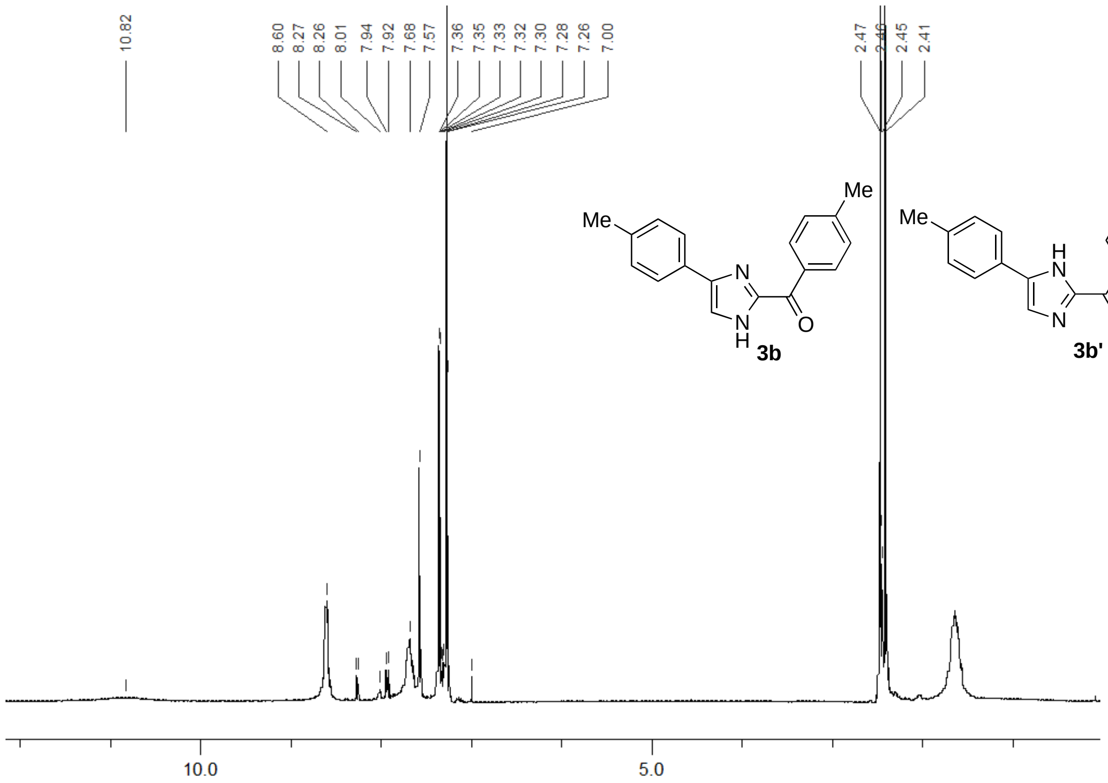

^{13}C NMR (150 MHz, $CDCl_3$)

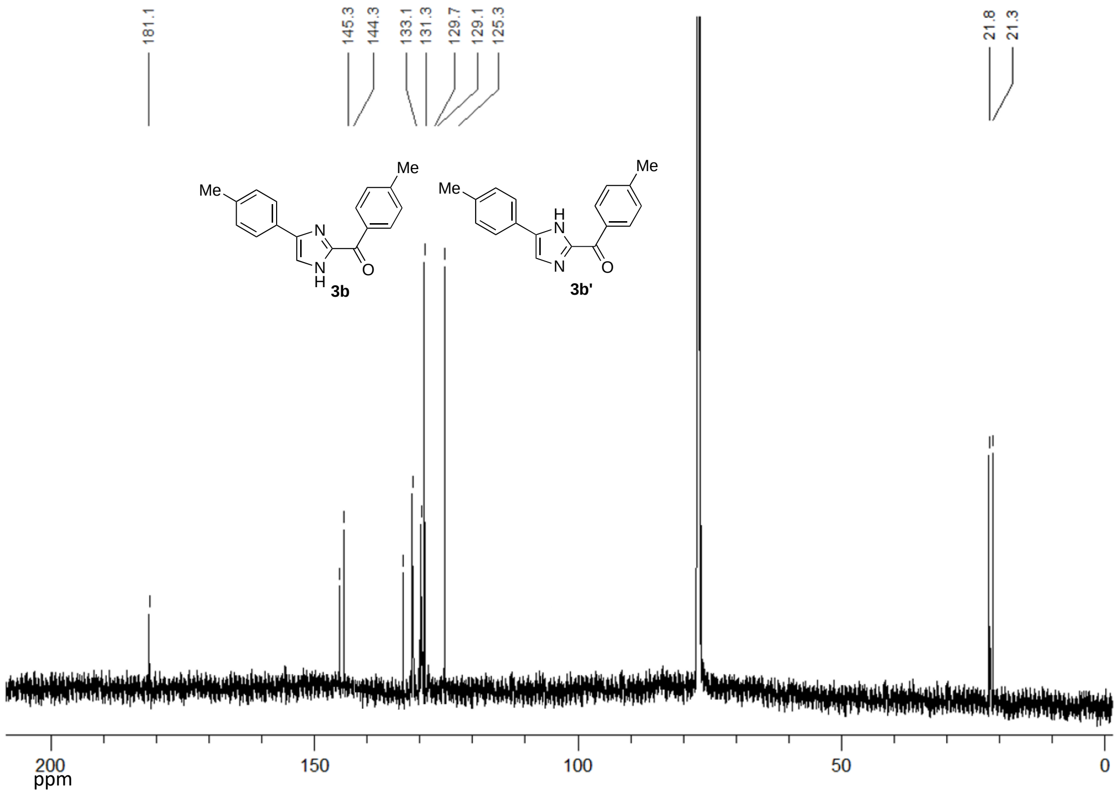

^{1}H and ^{13}C NMR spectra of **2-(4-chlorobenzoyl)-4-(4-chlorophenyl)-1*H*-imidazole** (**3c**) and **2-(4-chlorobenzoyl)-5-(4-chlorophenyl)-1*H*-imidazole** (**3c'**)

^{1}H NMR [600 MHz, $(CD_3)_2CO$]

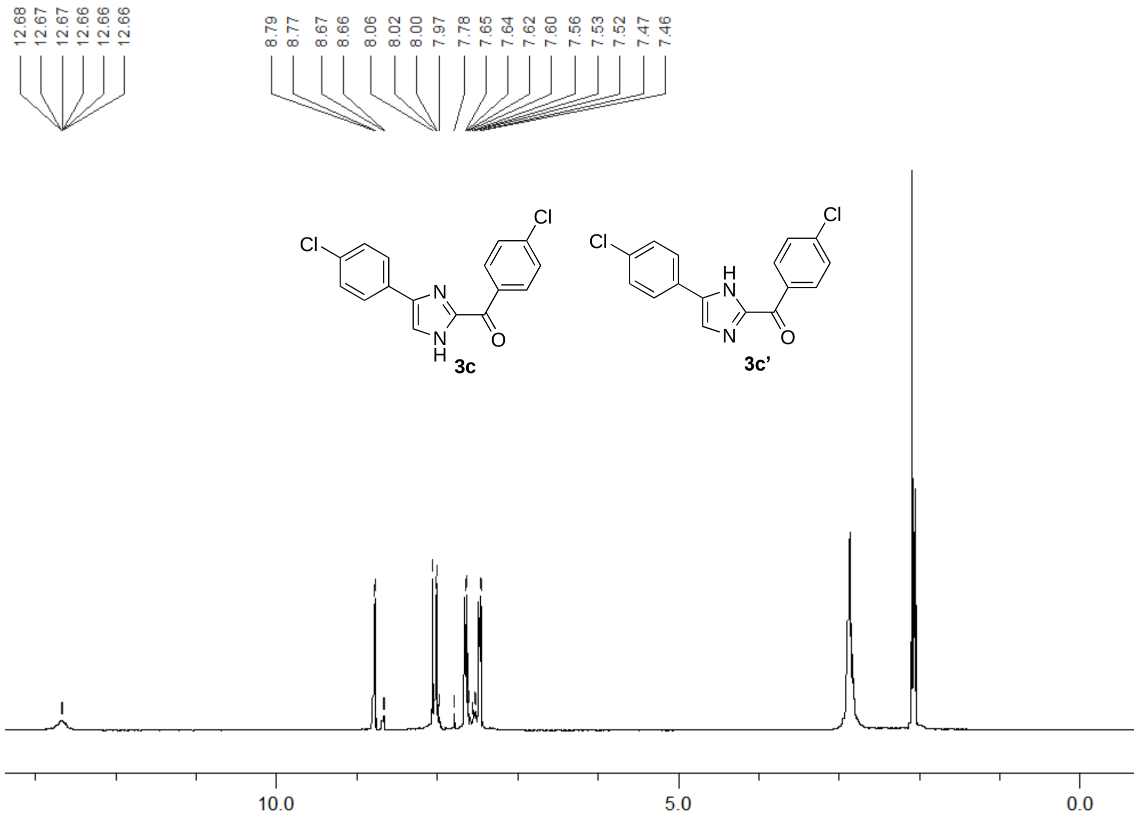

^{13}C NMR [150 MHz, $(CD_3)_2CO$]

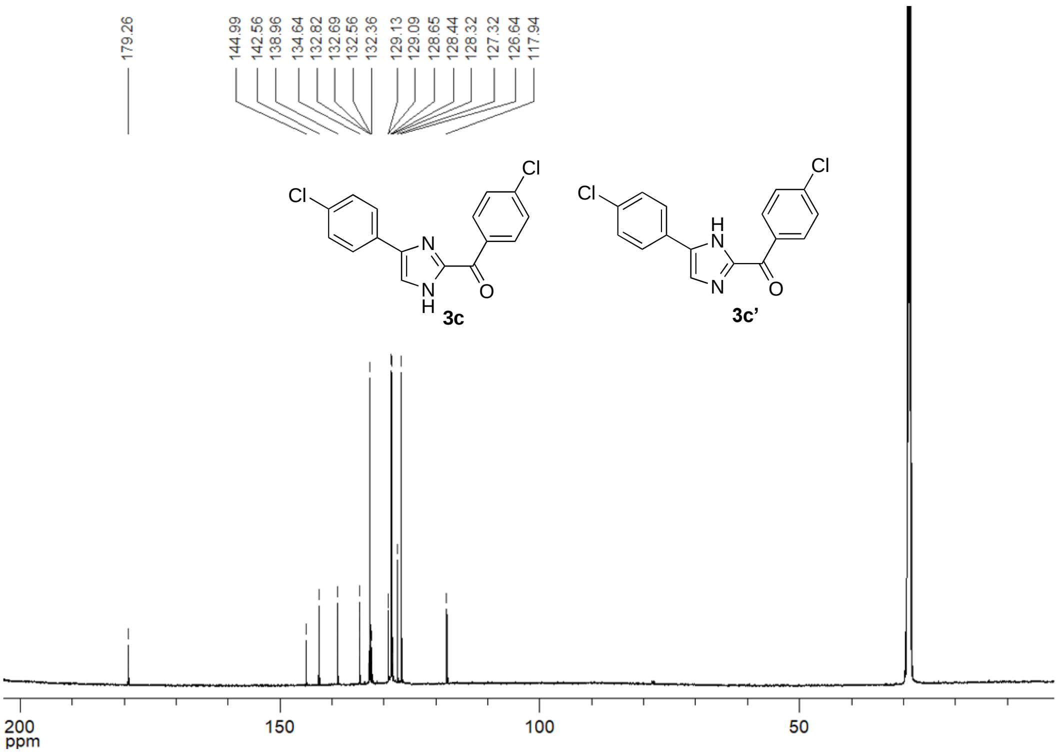

^{1}H and ^{13}C NMR spectra of **2-(4-bromobenzoyl)-4-(4-bromophenyl)-1*H*-imidazole** (**3d**)

^{1}H NMR [600 MHz, $(CD_3)_2SO$]

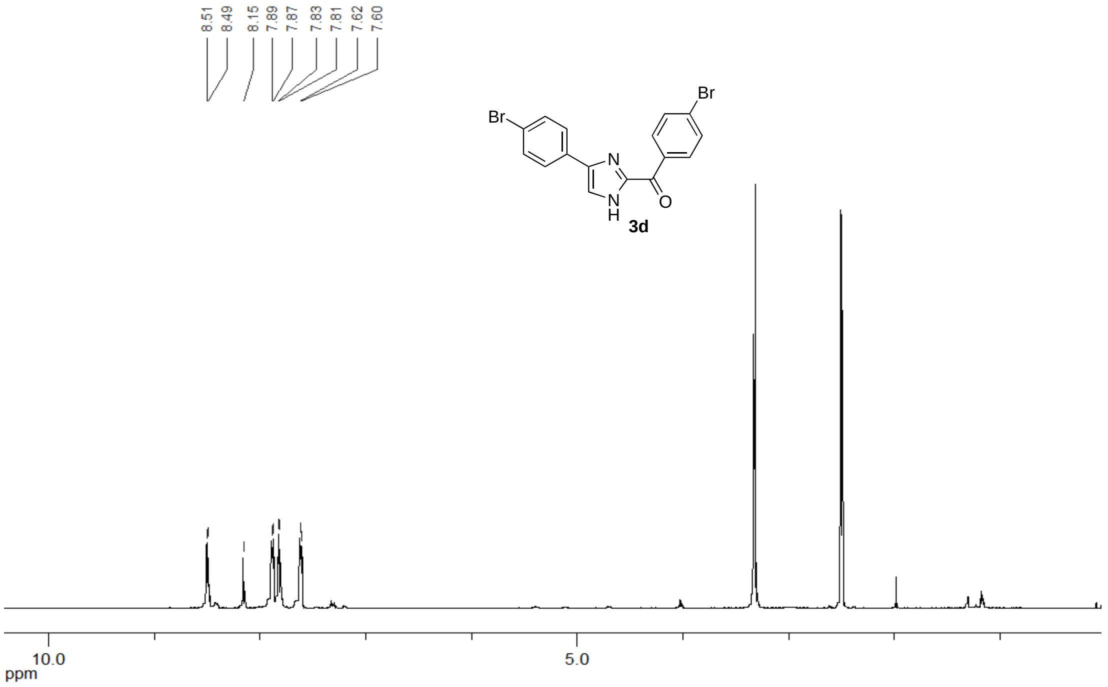

^{13}C NMR [150 MHz, $(CD_3)_2SO$]

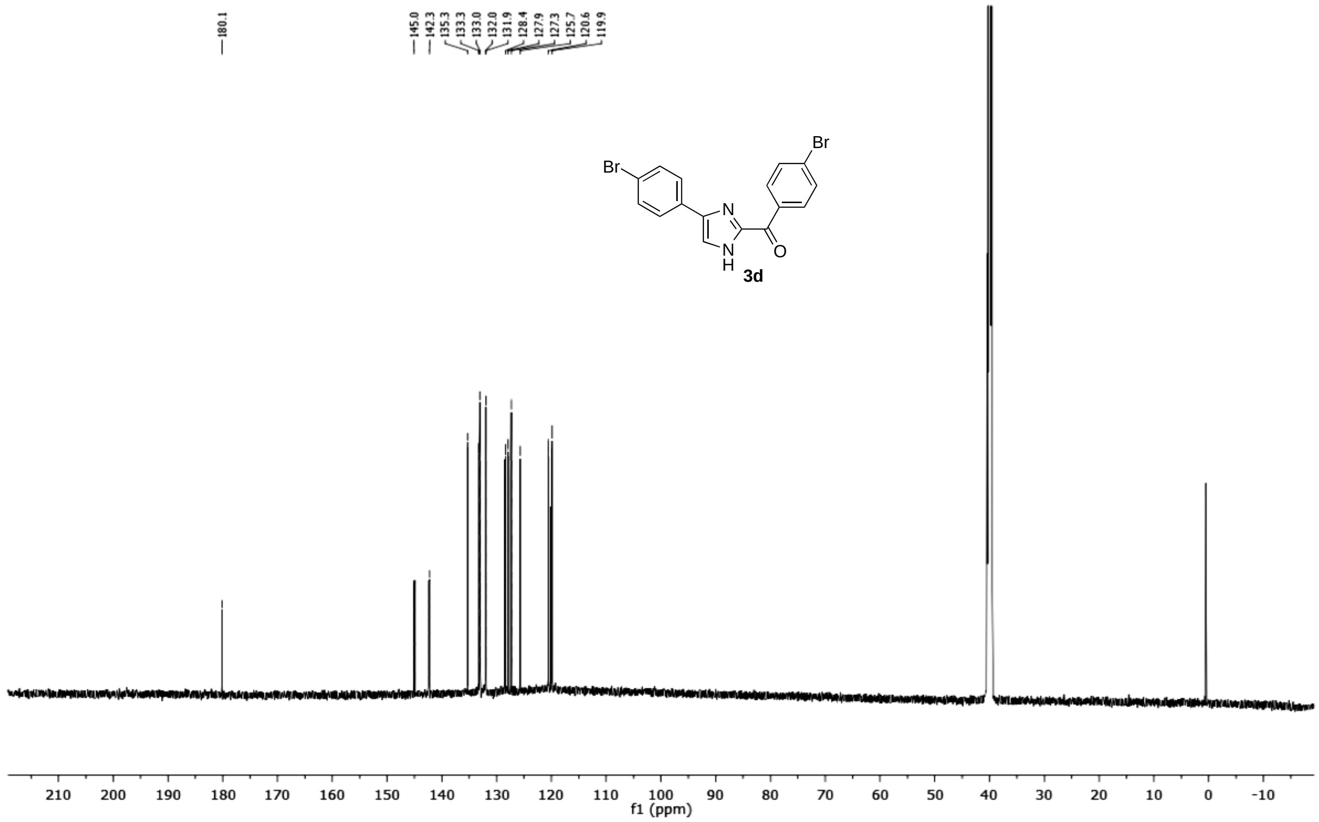

^{1}H and ^{13}C NMR spectra of **2-(4-cyanobenzoyl)-4-(4-cyanophenyl)-1*H*-imidazole** (**3e**) and **2-(4-cyanobenzoyl)-5-(4-cyanophenyl)-1*H*-imidazole** (**3e'**).

^{1}H NMR [600 MHz, $CDCl_3$]

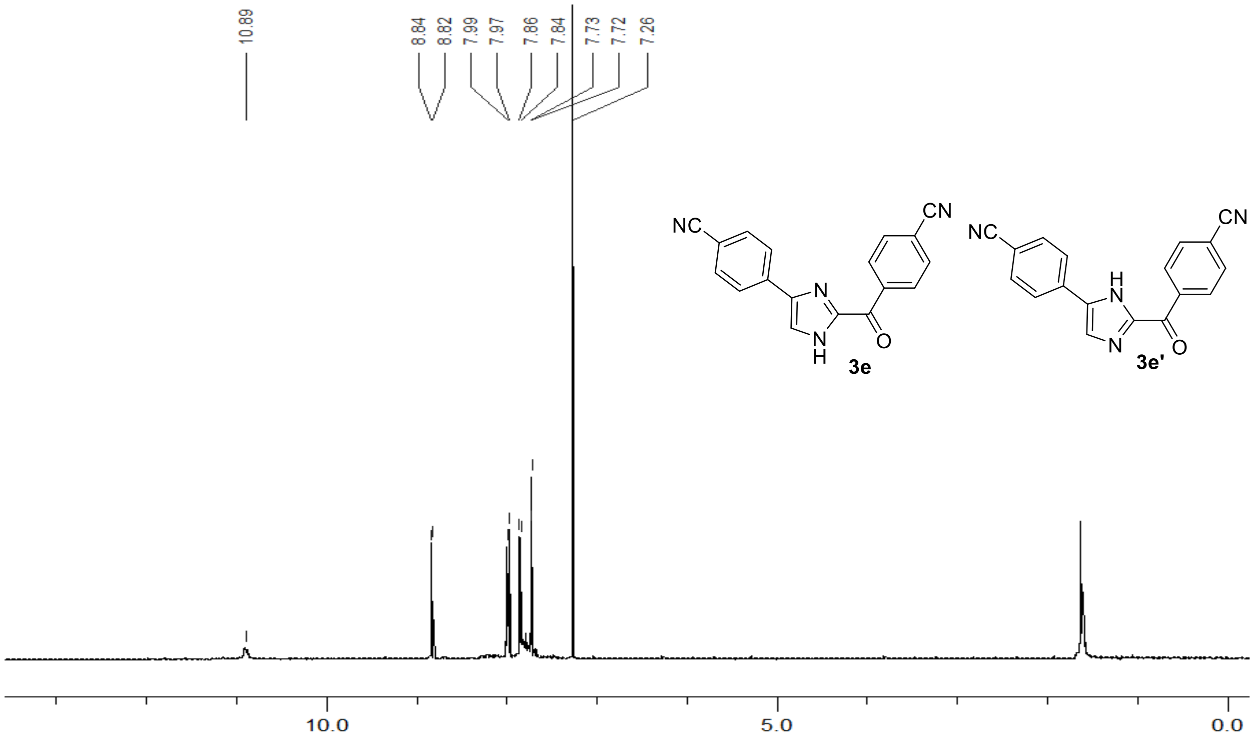

^{13}C NMR [100 MHz, $(CD_3)_2SO$, δ]

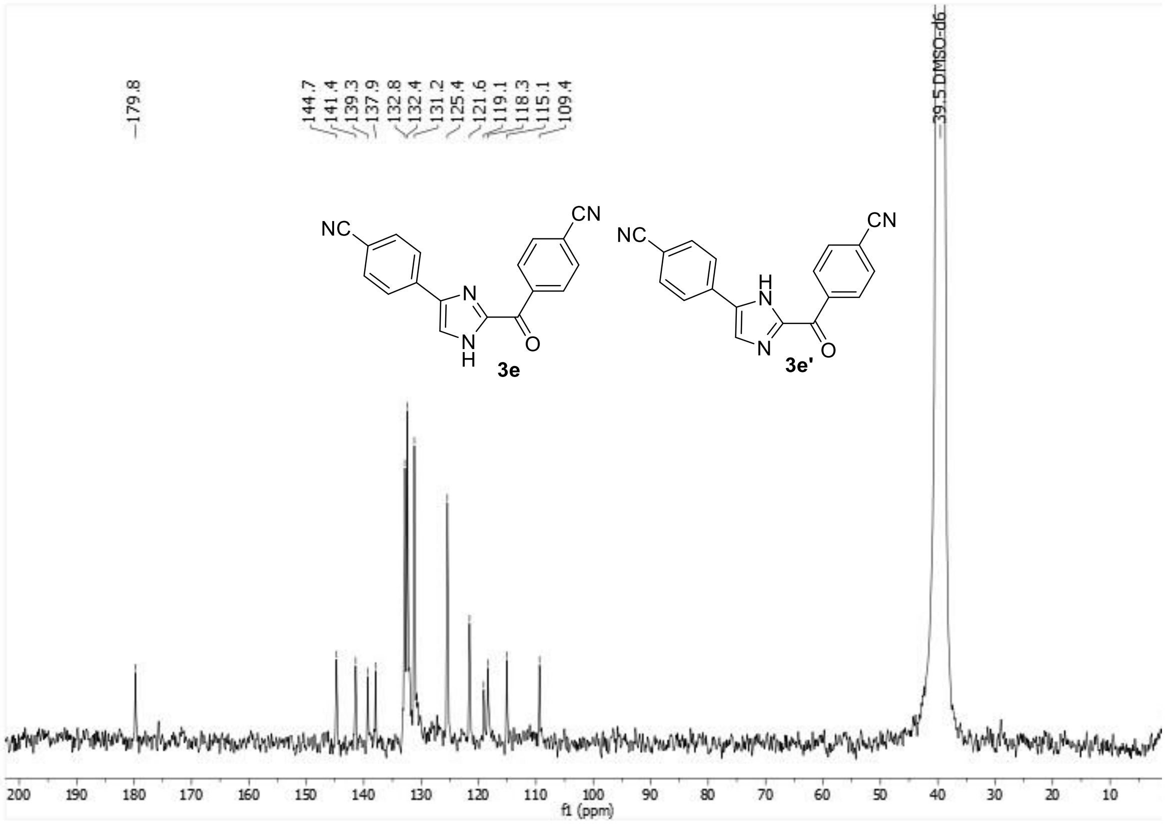

^{1}H and ^{13}C NMR spectra of **2-(4-methoxybenzoyl)-4-(4-methoxyphenyl)-1*H*-imidazole** (**3f**) and **2-(4-methoxybenzoyl)-5-(4-methoxyphenyl)-1*H*-imidazole** (**3f'**)

^{1}H NMR (600 MHz, $CDCl_3$)

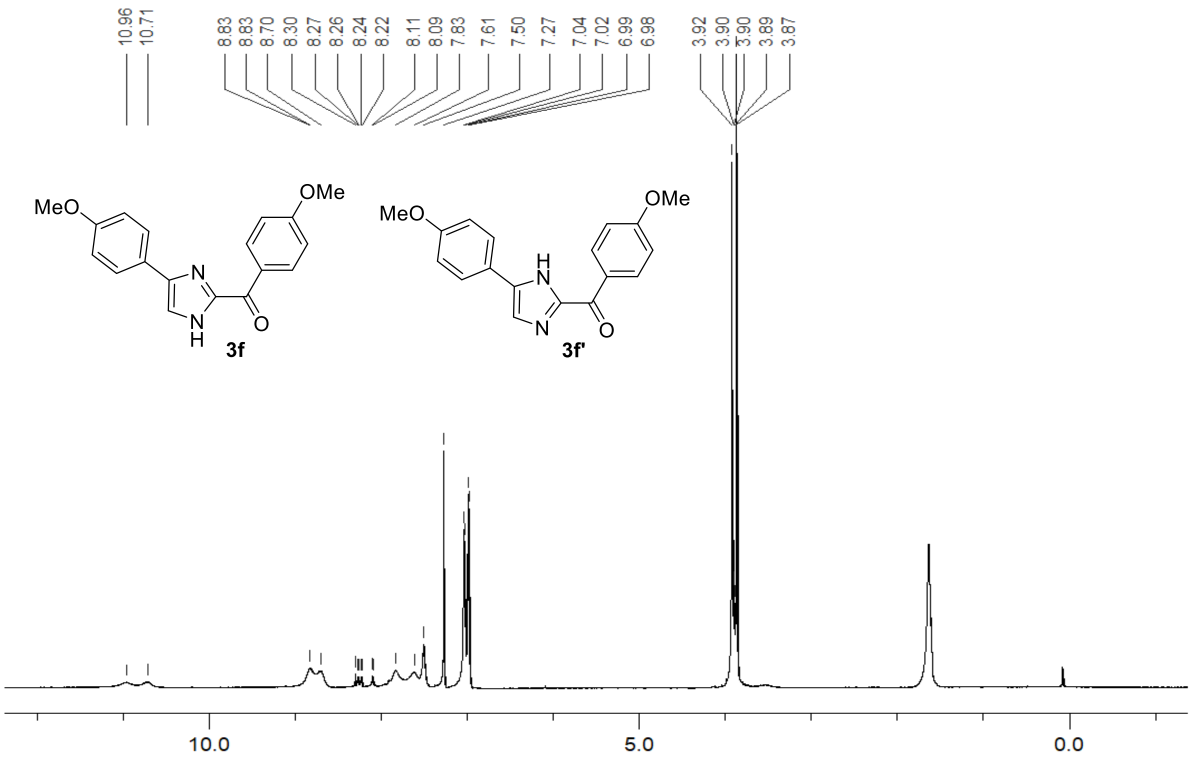

^{13}C NMR (150 MHz, $CDCl_3$)

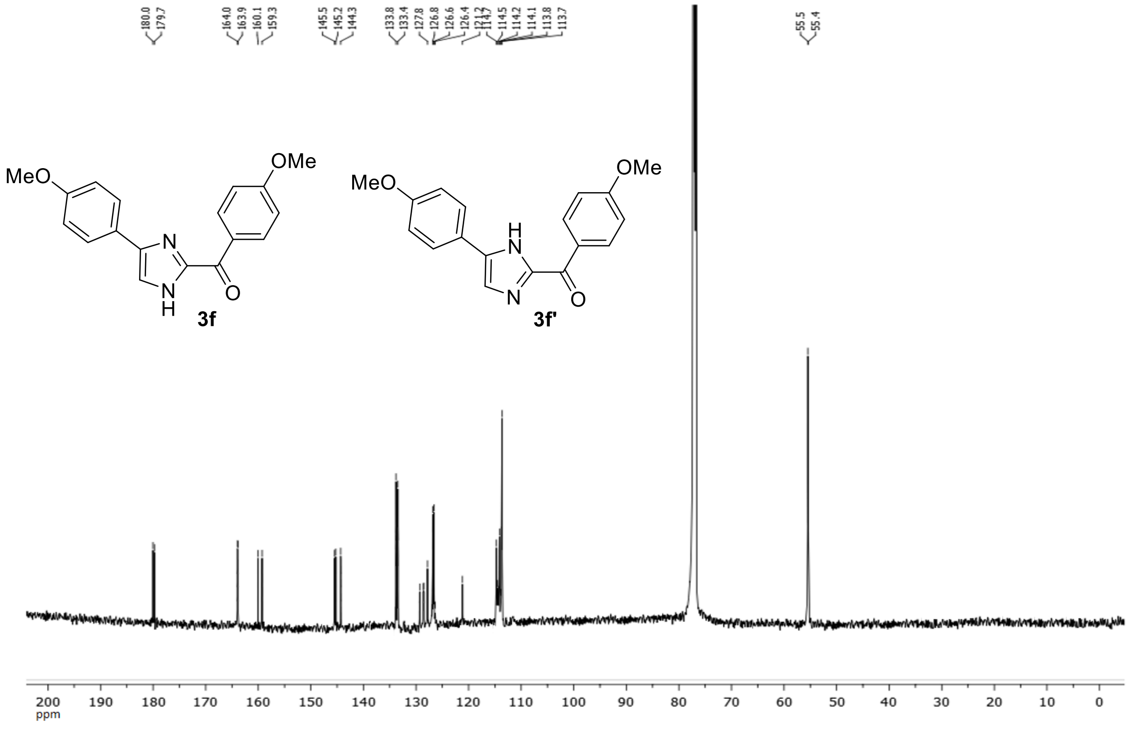

^{1}H and ^{13}C NMR spectra of **2-(2,5-dimethoxybenzoyl)-4-(2,5-dimethoxyphenyl)-1*H*-imidazole** (**3g**) and **2-(2,5-dimethoxybenzoyl)-5-(2,5-dimethoxyphenyl)-1*H*-imidazole** (**3g'**)

^{1}H NMR (600 MHz, $CDCl_3$)

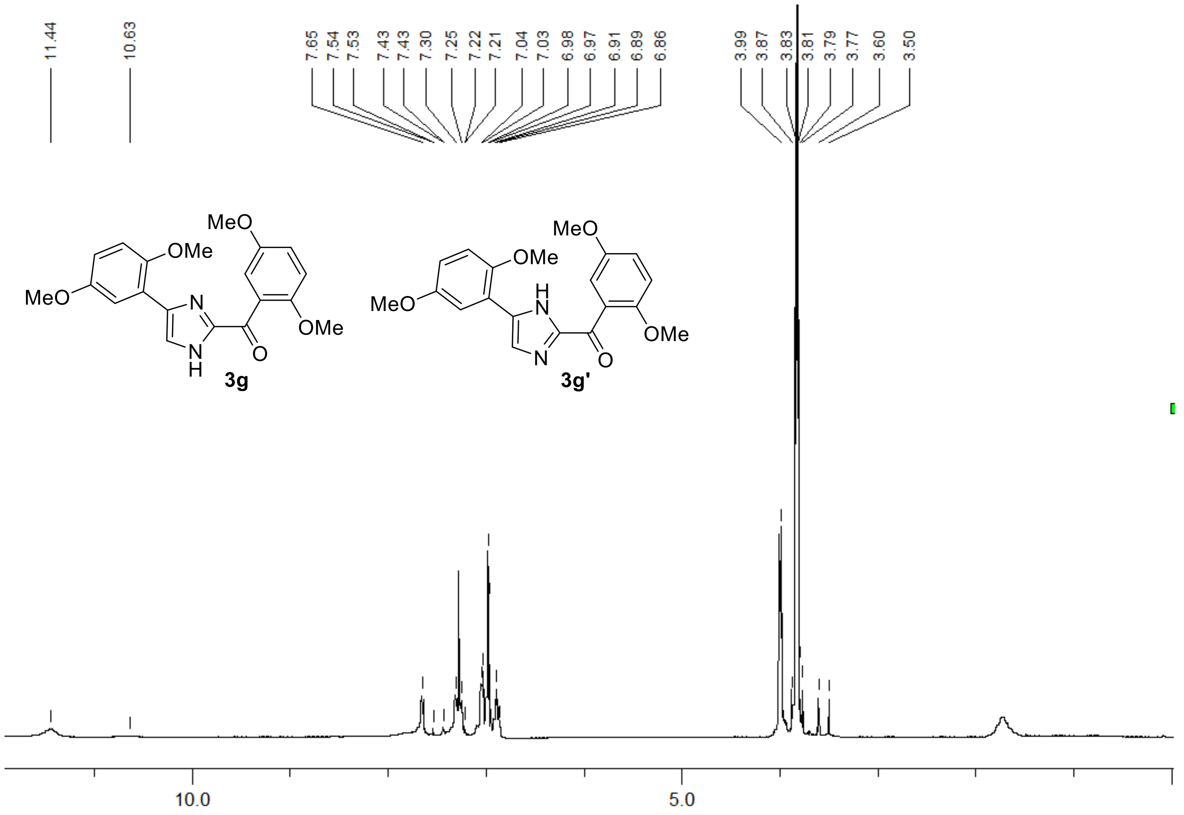

^{13}C NMR (150 MHz, $CDCl_3$)

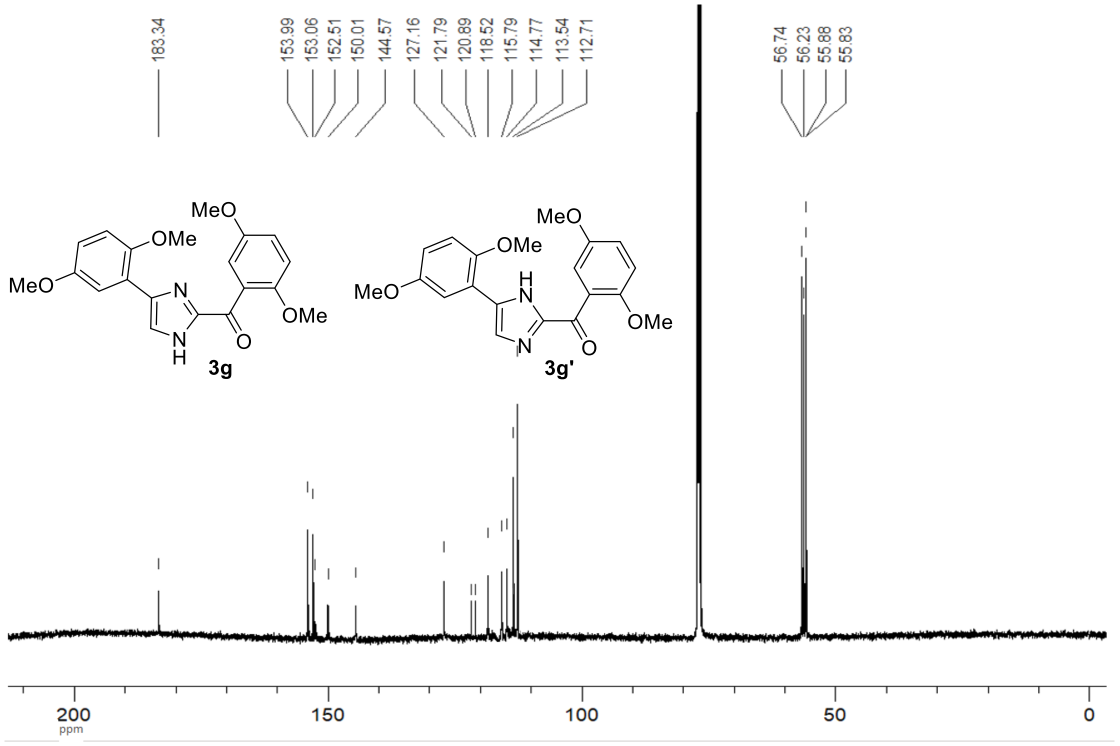

^{1}H and ^{13}C NMR spectra of **2-(2-hydroxybenzoyl)-4-(2-hydroxyphenyl)-1*H*-imidazole** (**3h**).

^{1}H NMR [300 MHz, $CDCl_3$]

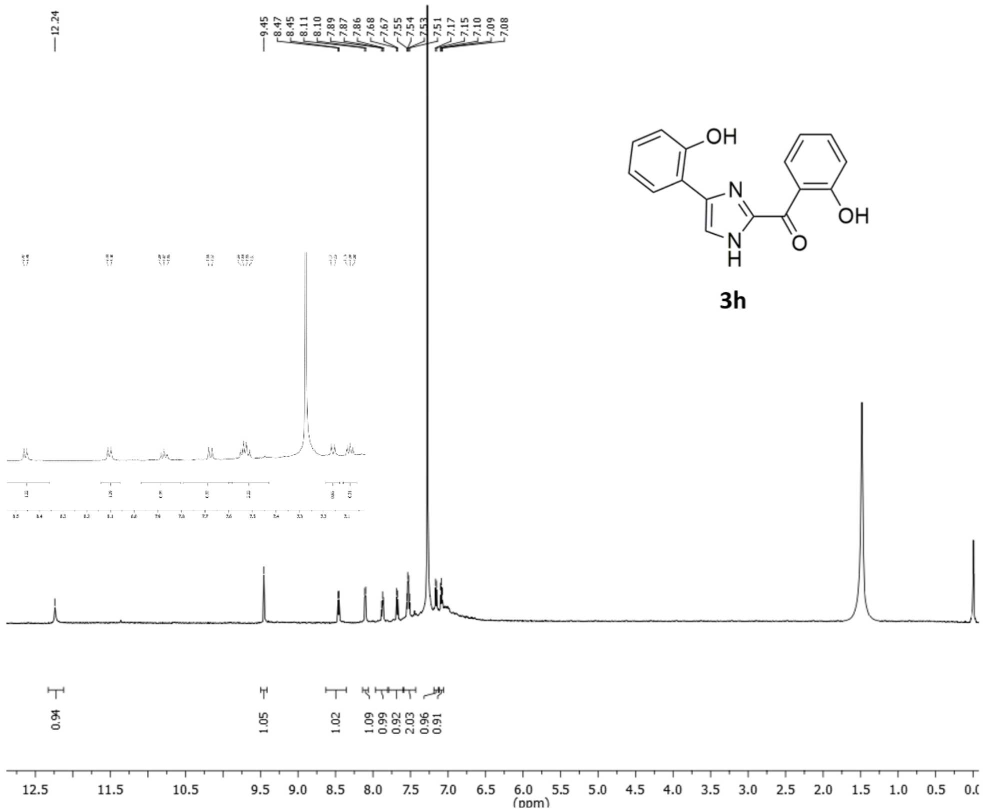

^{13}C NMR [75 MHz, $CDCl_3$]

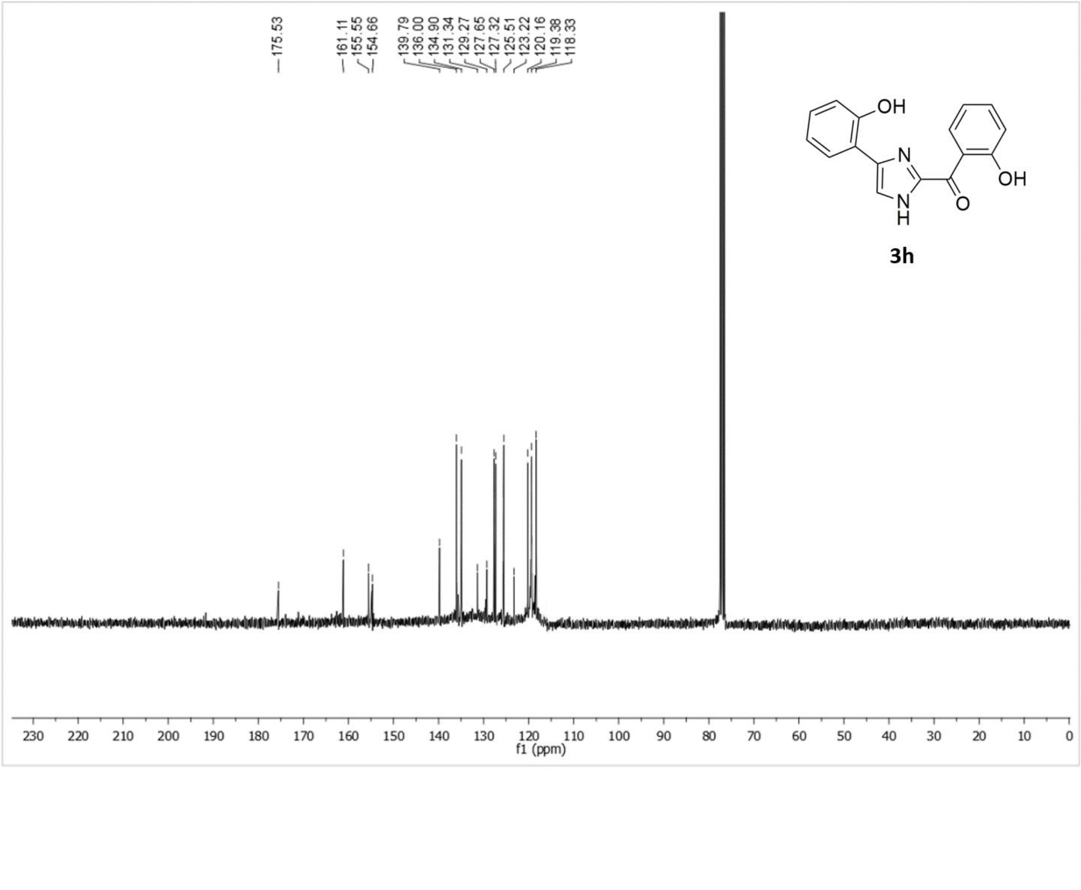

^{1}H and ^{13}C NMR spectra of **2-(4-fluorobenzoyl)-4-(4-fluorophenyl)-1*H*-imidazole** (**3i**) and **2-(4-fluorobenzoyl)-5-(4-fluorophenyl)-1*H*-imidazole** (**3i'**).

^{1}H NMR [600 MHz, $(CD_3)_2CO$]

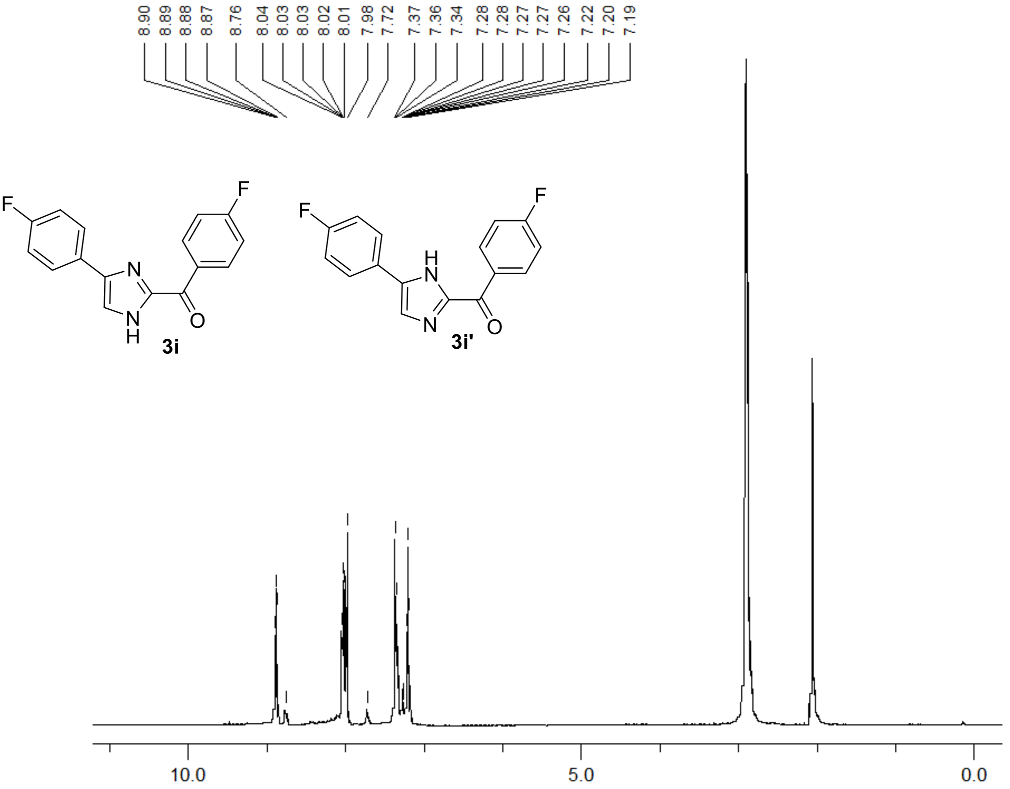

^{13}C NMR[150 MHz, $(CD_3)_2CO$]

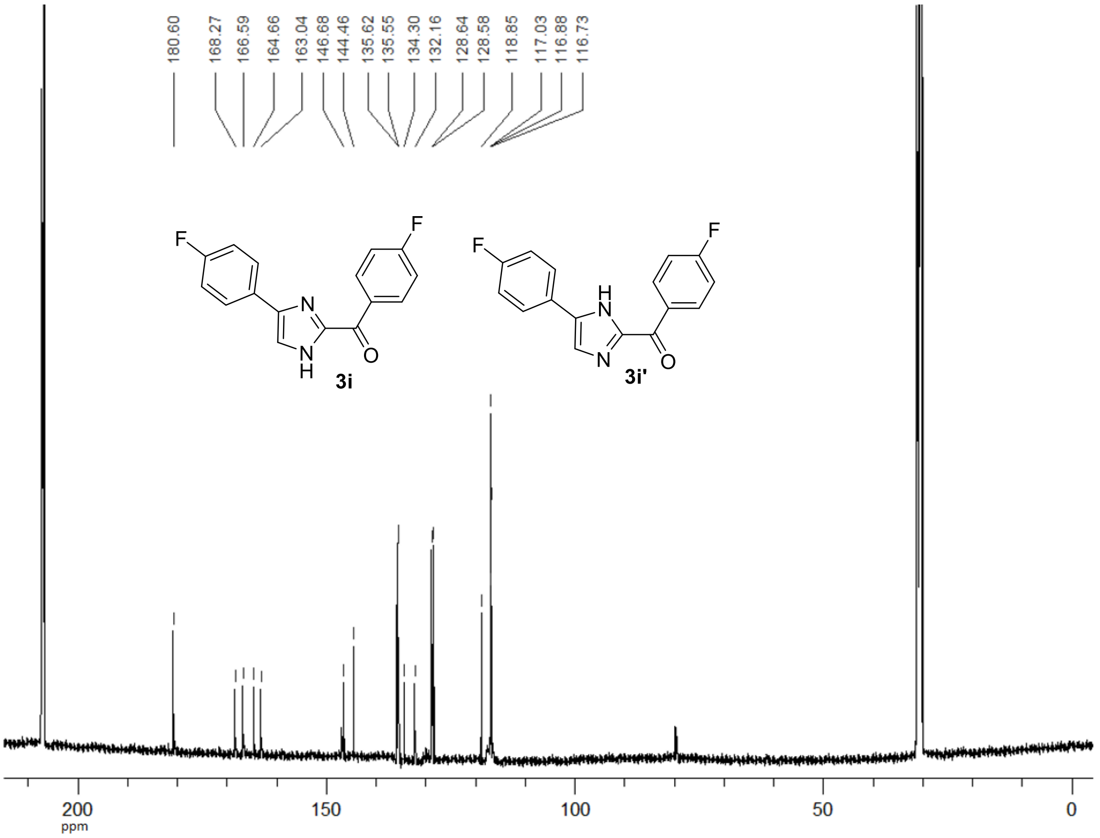

^{1}H and ^{13}C NMR spectra of **4-(2-naphtyl)-2-(2-naphtyloyl)-1*H*-imidazole** (**3j**)

^{1}H NMR [600 MHz, $(CD_3)_2SO$, δ]

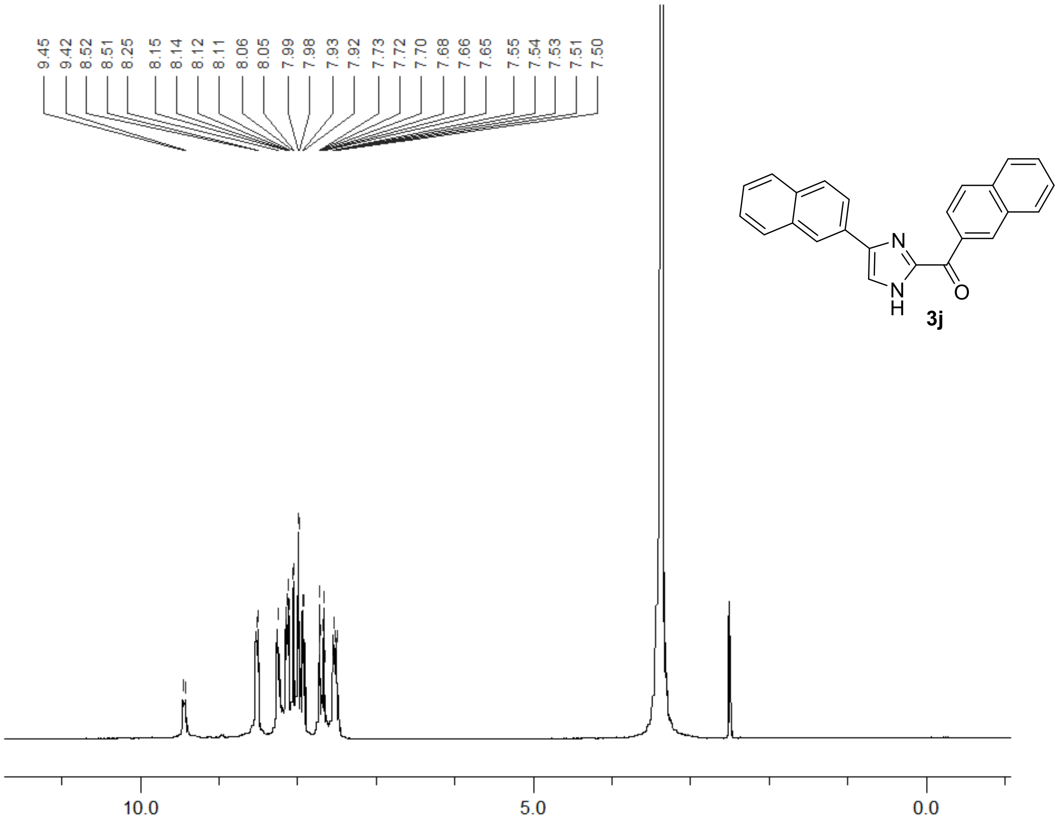

^{13}C NMR[150 MHz, $(CD_3)_2CO$, δ]

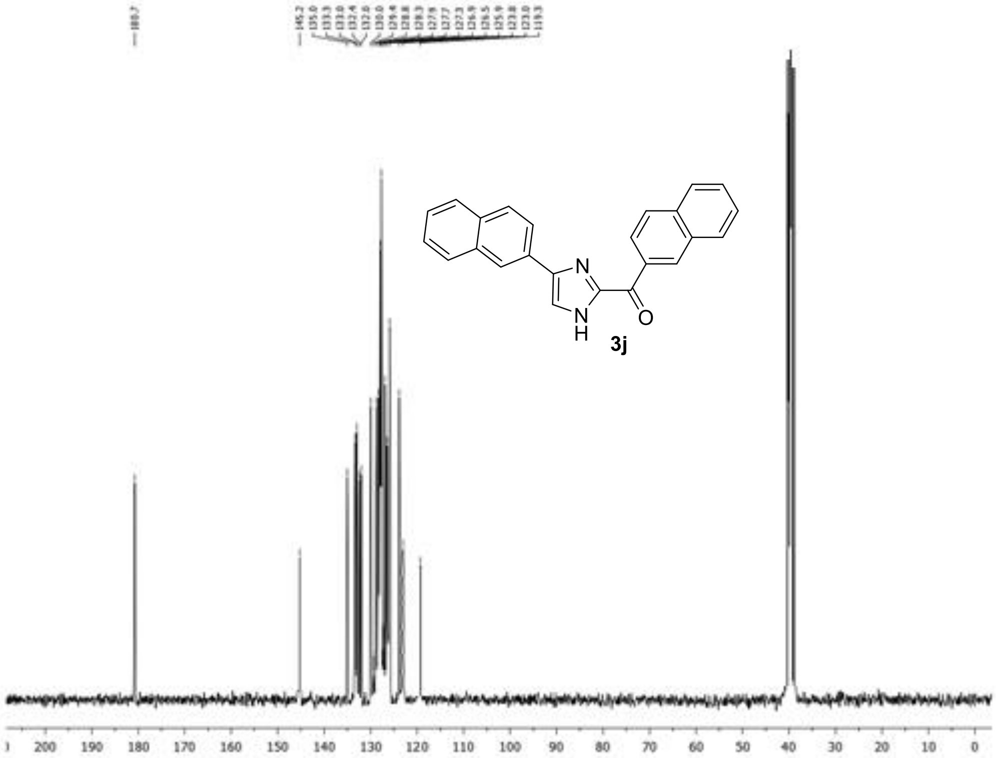

^{1}H and ^{13}C NMR spectra of **4-[1,1'-biphenyl-4-yl]-2-(4-phenybenzoyl)-1*H*-imidazole** (**3k**) and **5-[1,1'-biphenyl-4-yl]-2-(4-phenybenzoyl)-1H-imidazole** (**3k'**)

^{1}H NMR [600 MHz, $(CD_3)_2SO$]

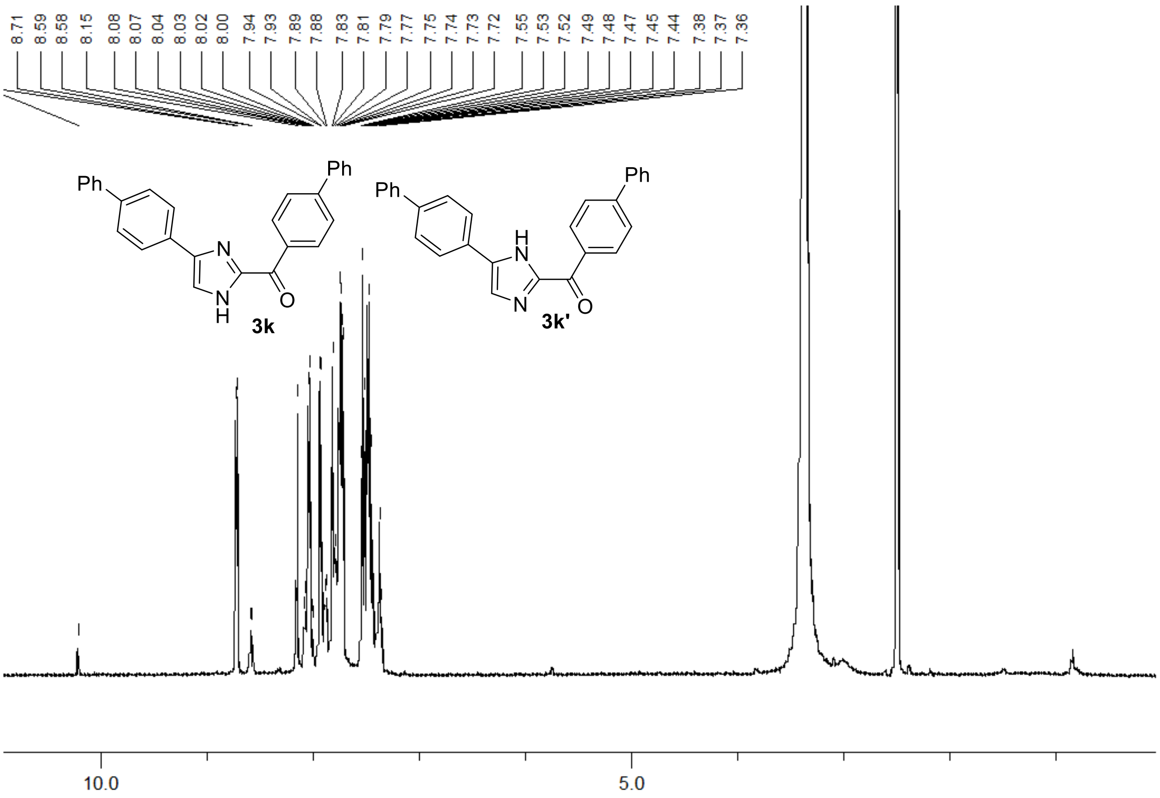

^{13}C NMR[150 MHz, $(CD_3)_2SO$]

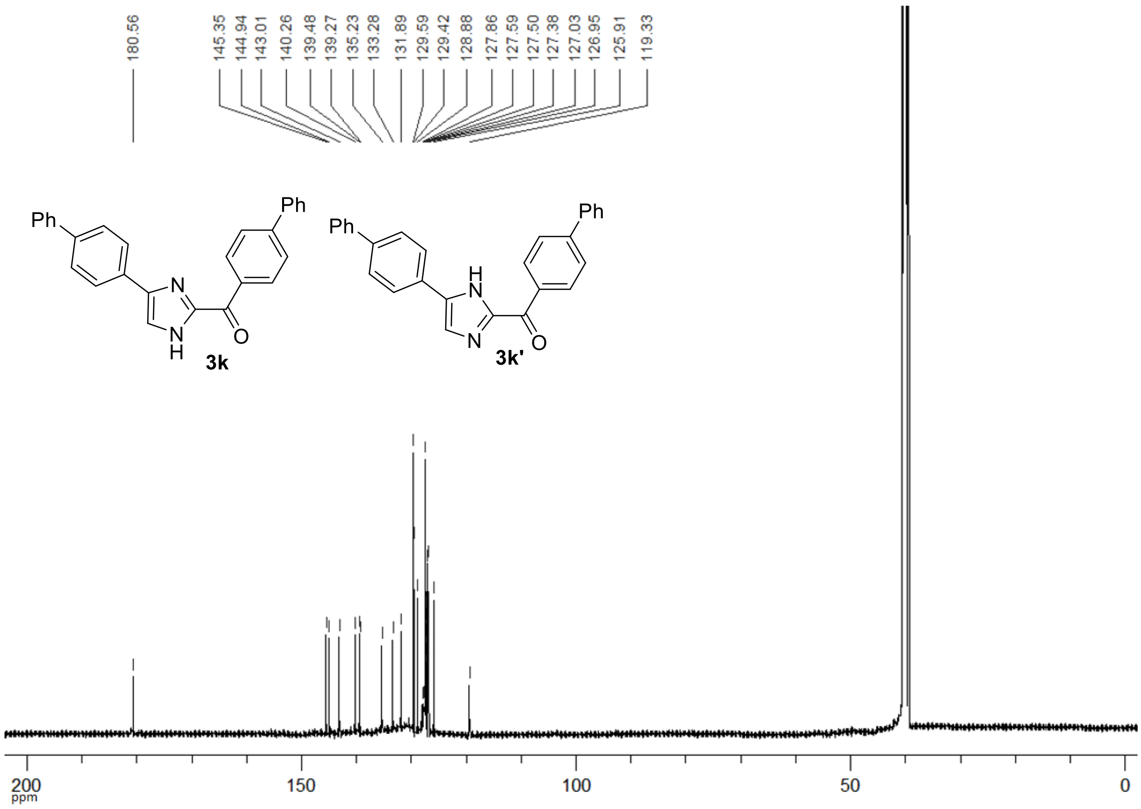

2.3 NMR spectra of 2,4-diaroyl-6-arylpyrimidines 7a–h

^{1}H and ^{13}C NMR spectra of **2,4-dibenzoyl-6-phenylpyrimidine** (**7a**)

^{1}H NMR (600 MHz, $CDCl_3$)

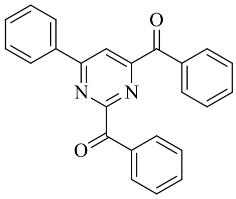

7a

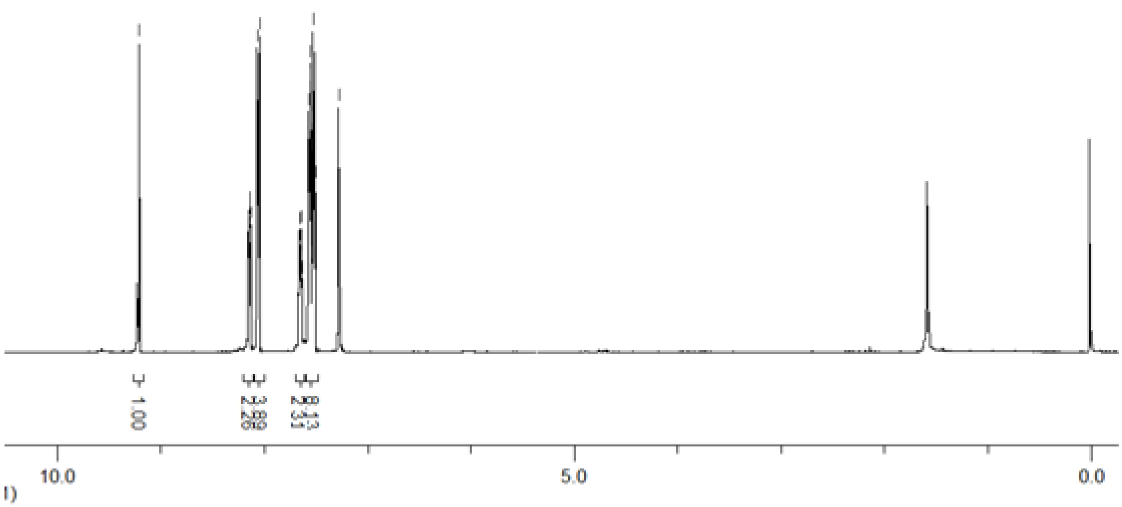

^{13}C NMR (150 MHz, $CDCl_3$, δ)

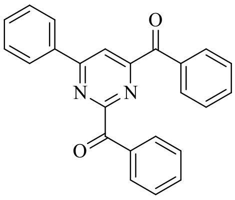

7a

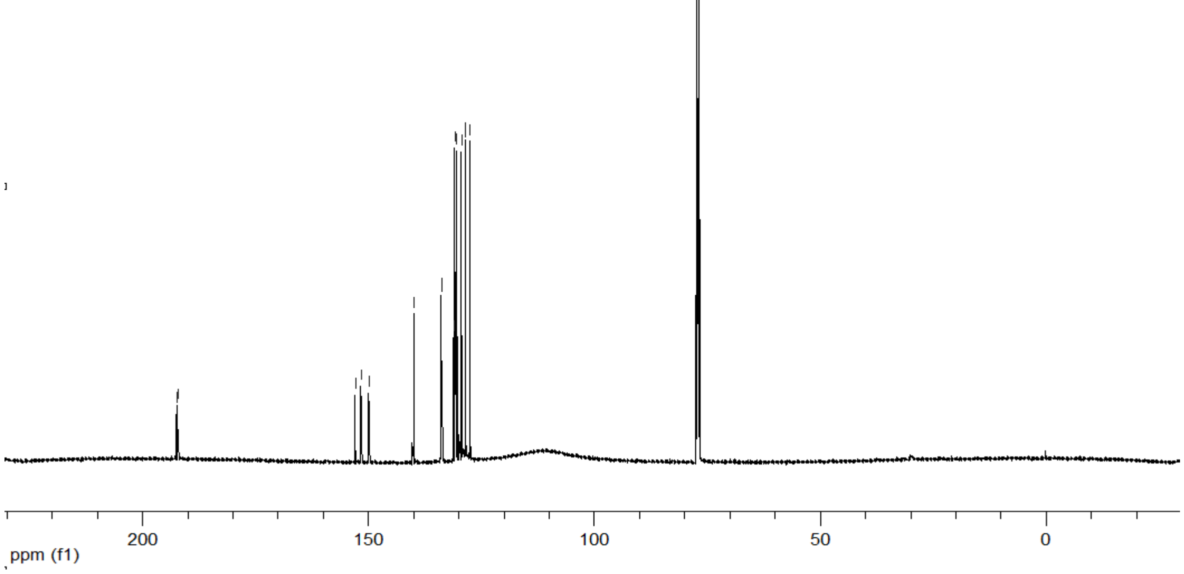

^{1}H and ^{13}C NMR spectra of **2,4-bis-(4-methylbenzoyl)-6-tolylpyrimidine** (**7b**)

^{1}H NMR (600 MHz, $CDCl_3$)

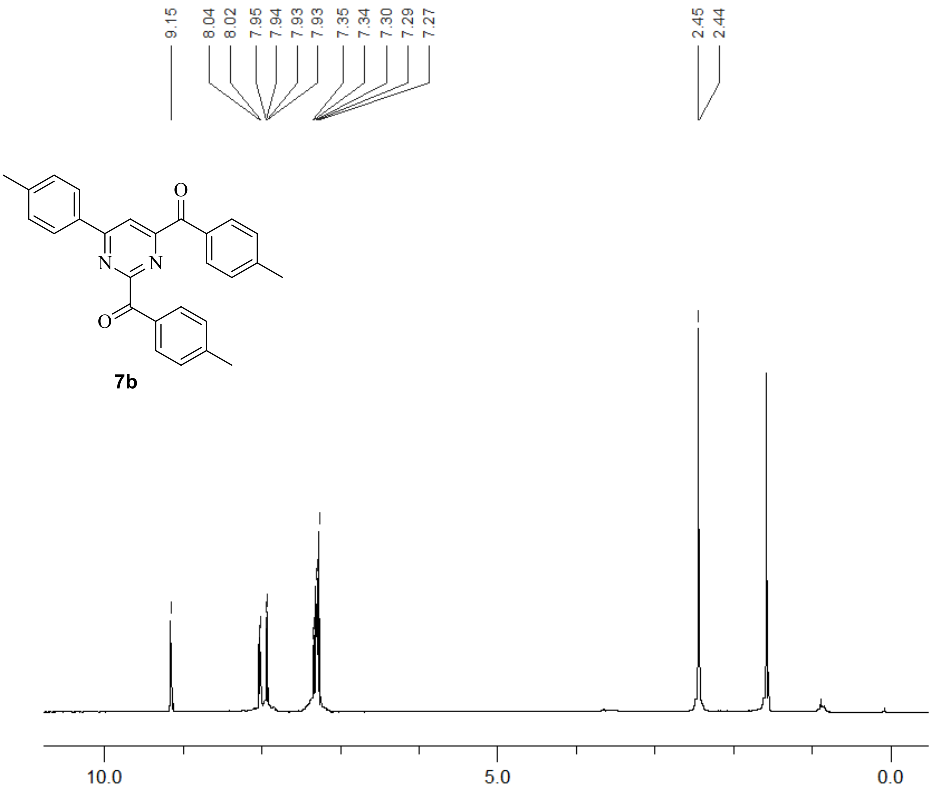

^{13}C NMR (150 MHz, $CDCl_3$)

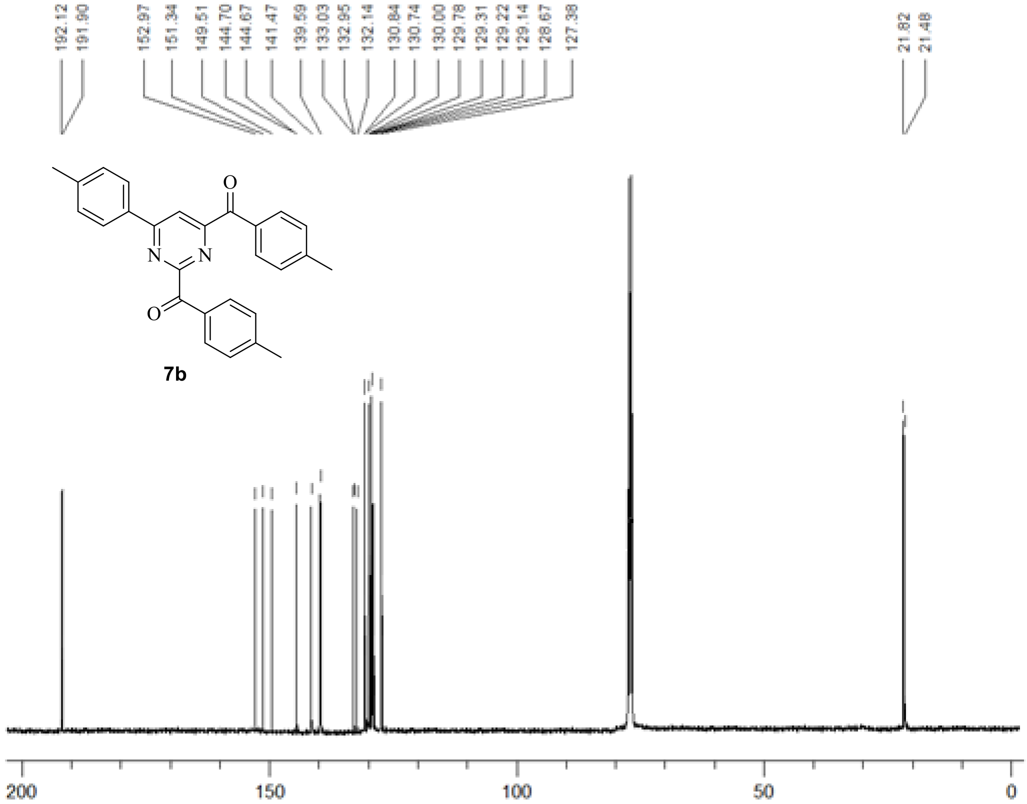

^{1}H and ^{13}C NMR spectra of **2,4-bis(4-chlorobenzoyl)-6-(4-chlorophenyl)pyrimidine** (**7c**)

^{1}H NMR (600 MHz, $CDCl_3$)

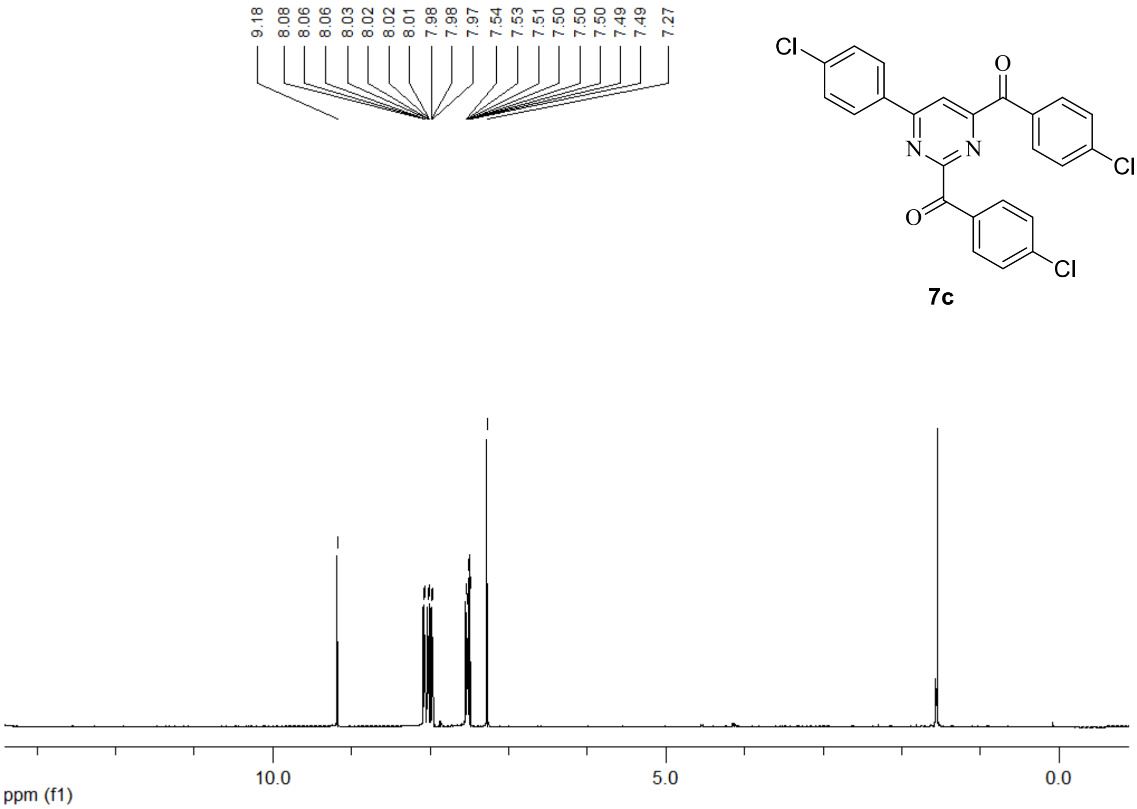

^{13}C NMR (150 MHz, $CDCl_3$, δ)

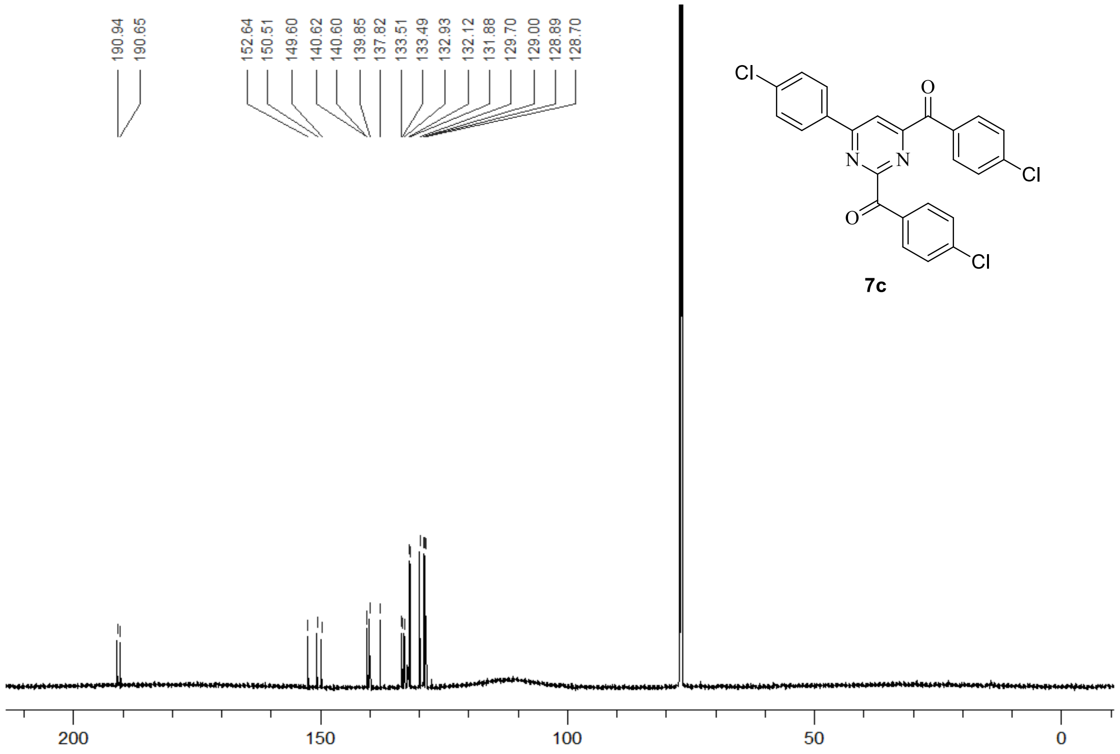

^{1}H and ^{13}C NMR spectra of **2,4-bis(4-bromobenzoyl) 6-(4-bromophenyl)pyrimidine** (**7d**)

^{1}H NMR (600 MHz, $CDCl_3$)

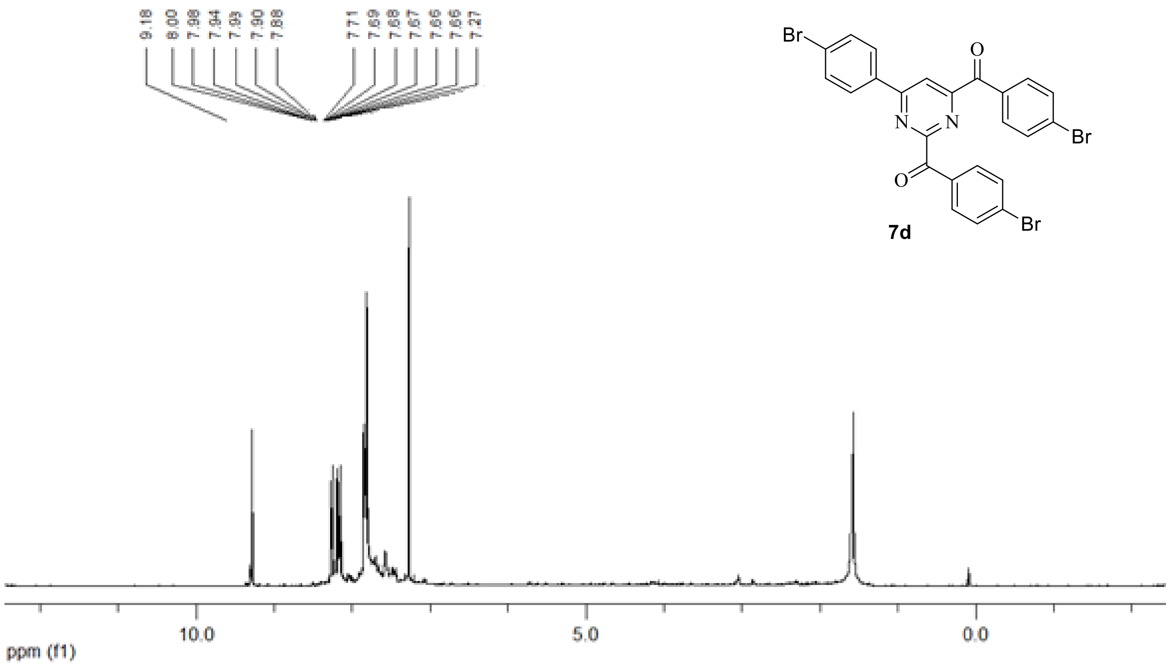

7d

^{13}C NMR (150 MHz, $CDCl_3$)

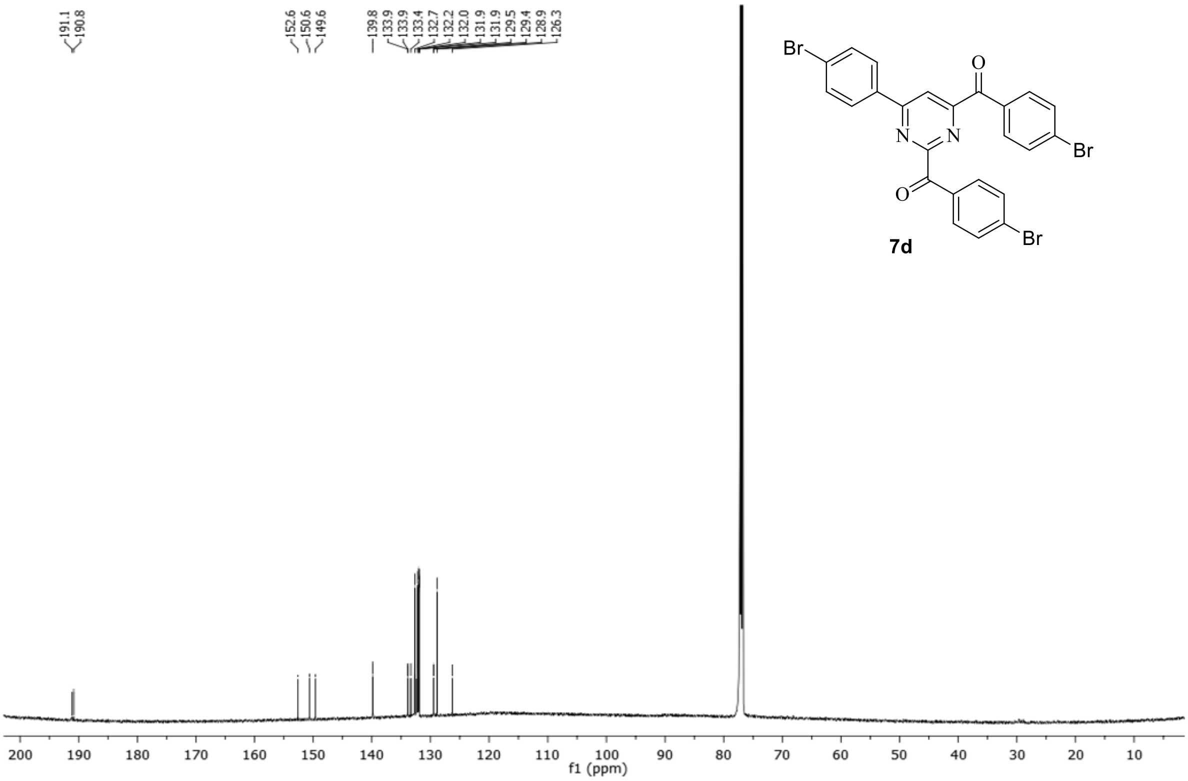

7d

^{1}H and ^{13}C NMR spectra of **2,4-bis-[2-(hydroxybenzoyl)]-6-(2-hydroxyphenyl) pyrimidine** (**7e**)

^{1}H NMR (600 MHz, $CDCl_3$)

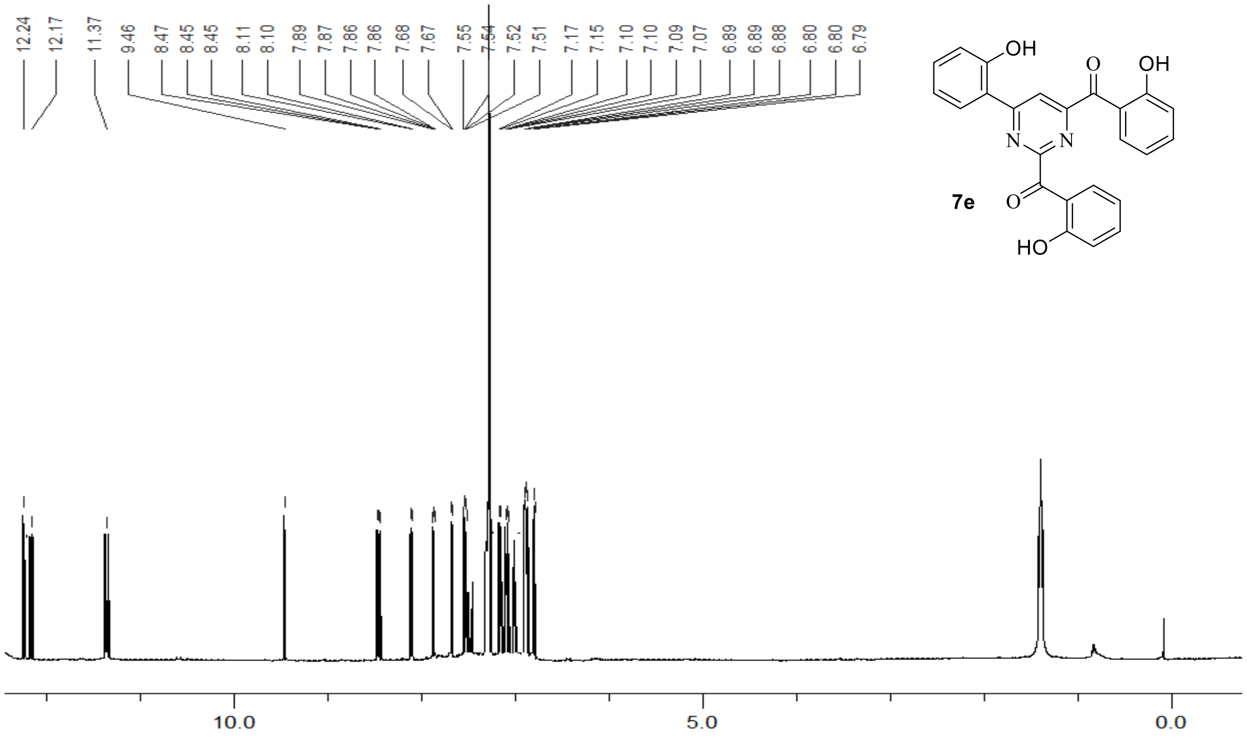

^{13}C NMR (150 MHz, $CDCl_3$)

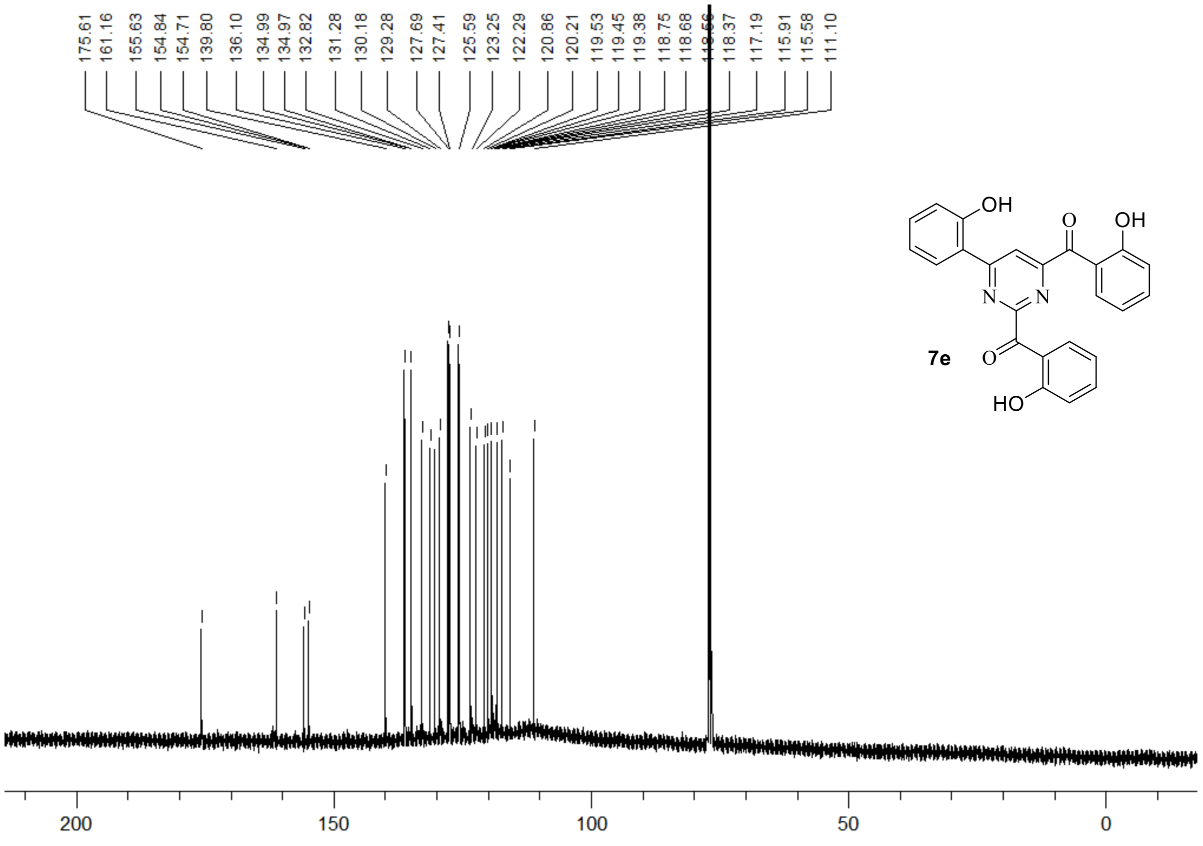

¹H and ¹³C NMR spectra of **2,4-bis(4-fluorobenzoyl)-6-(4-fluorophenyl)pyrimidine** (**7f**)

¹H NMR (600 MHz, CDCl₃)

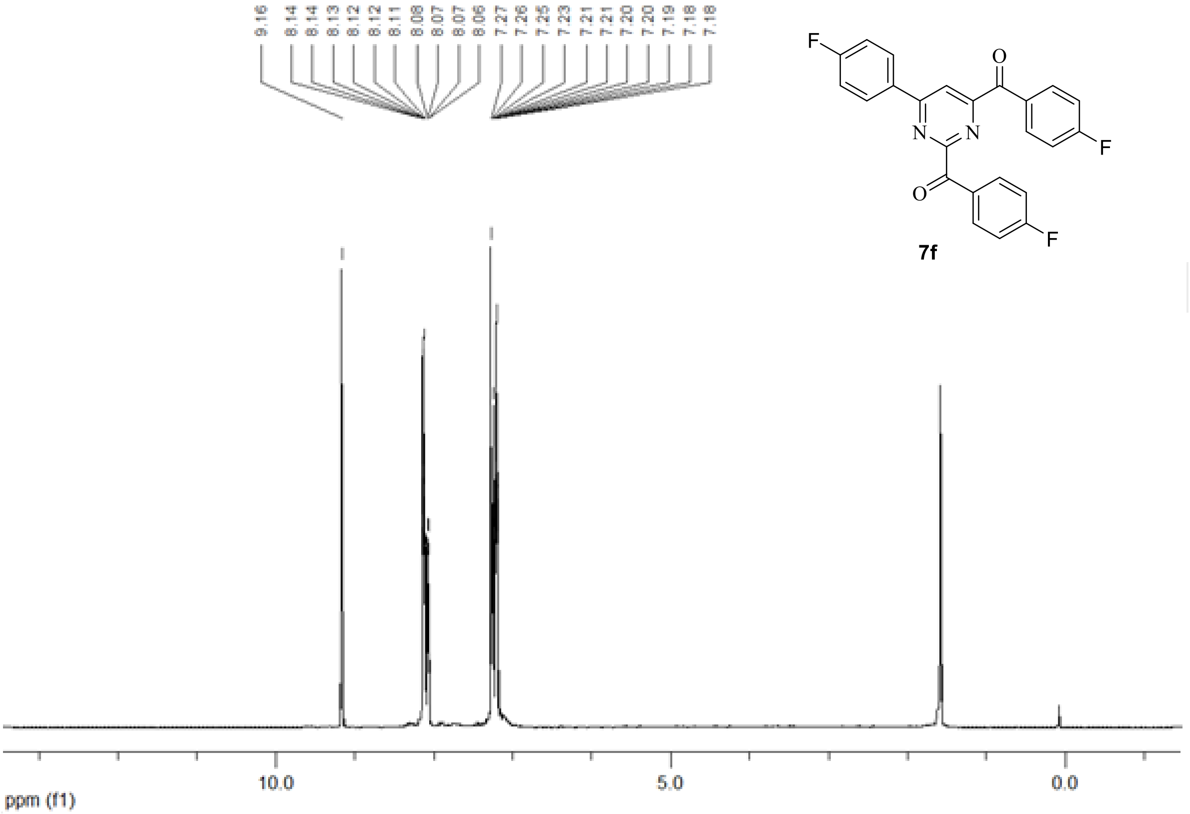

¹³C NMR (150 MHz, CDCl₃)

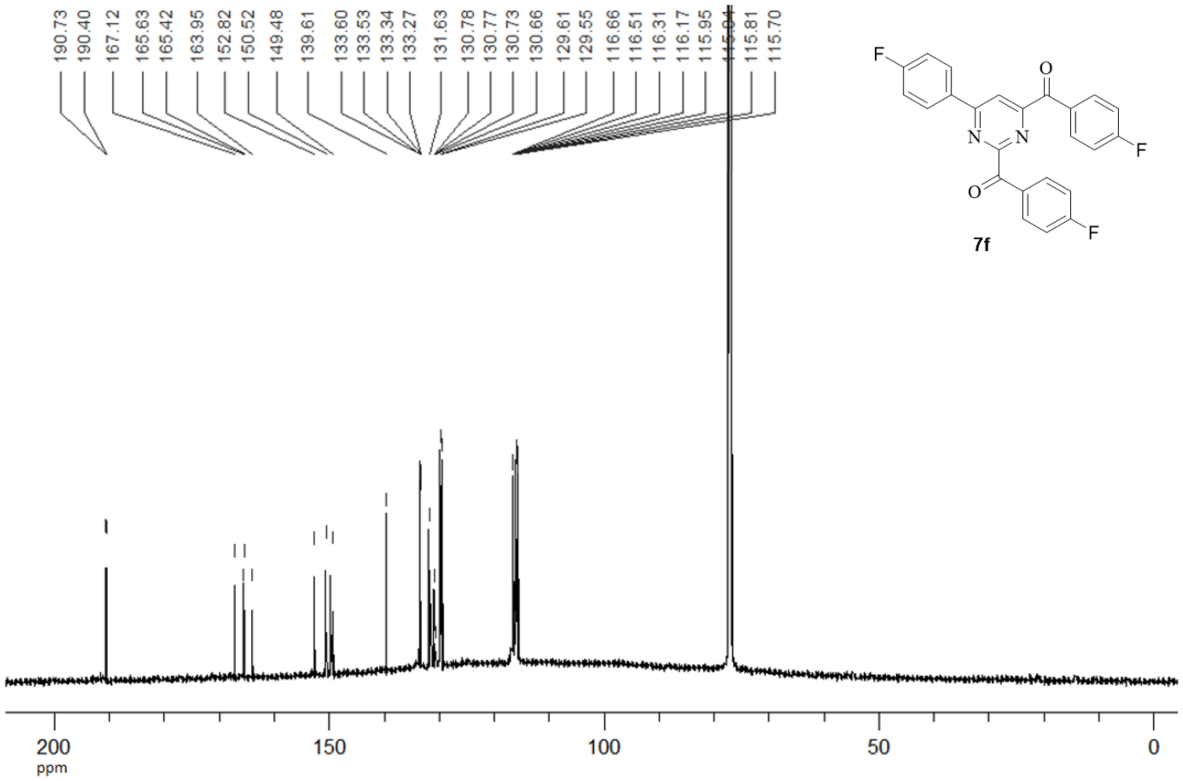

^{1}H and ^{13}C NMR spectra of **2,4-bis[2-(trifluoromethyl)benzoyl]-6-[2-(trifluoromethyl)phenyl]pyrimidine** (**7g**)

^{1}H NMR (600 MHz, $CDCl_3$)

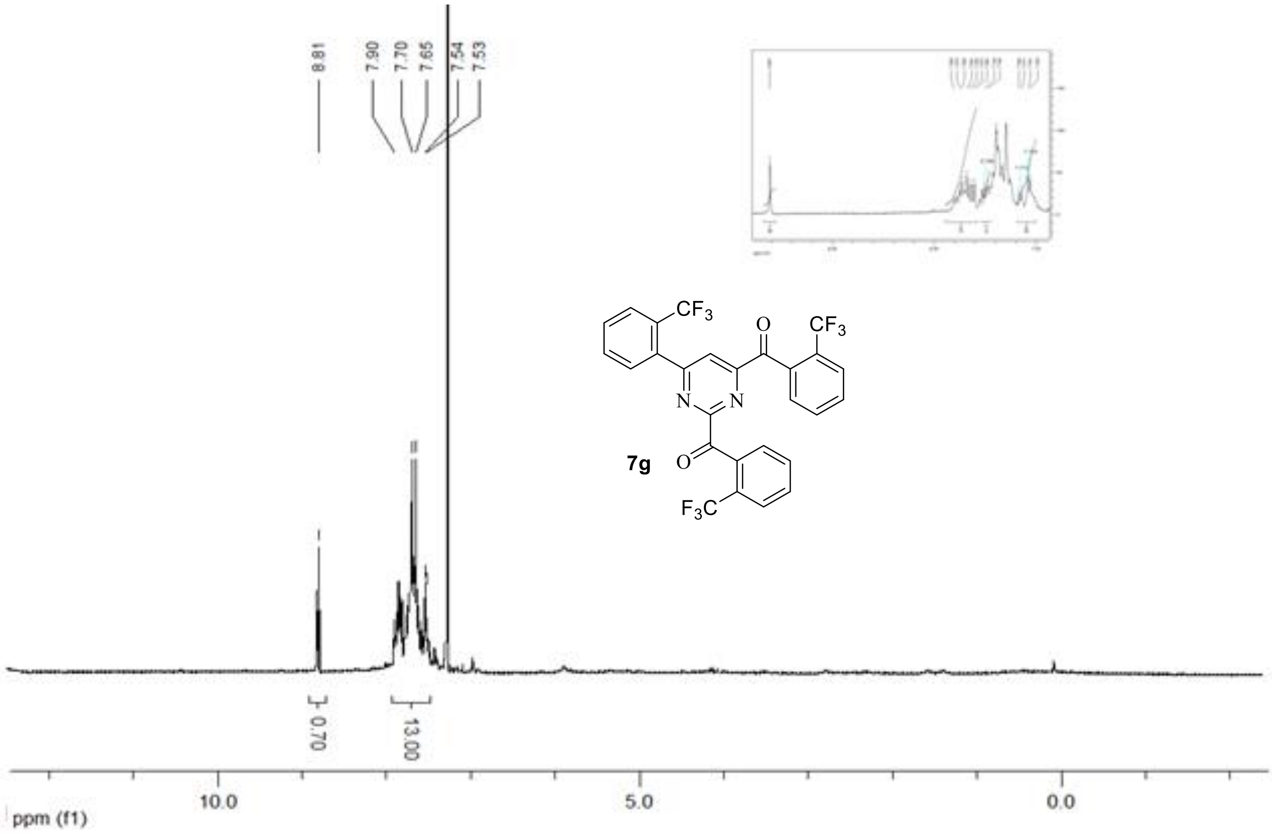

^{13}C NMR (150 MHz, $CDCl_3$)

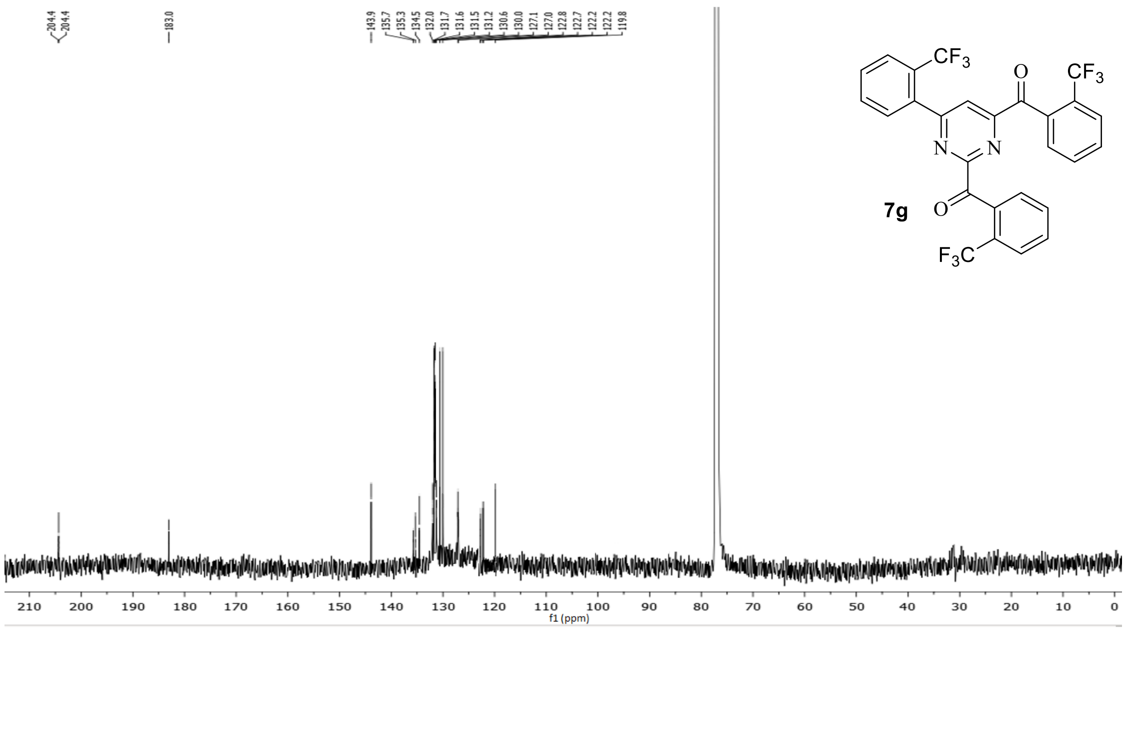

^1H and ^{13}C NMR spectra of **2,4-bis-[4-(trifluoromethyl)benzoyl]-6-(4-trifluoromethyl)phenylpyrimidine** (**7h**)

^1H NMR (600 MHz, $CDCl_3$)

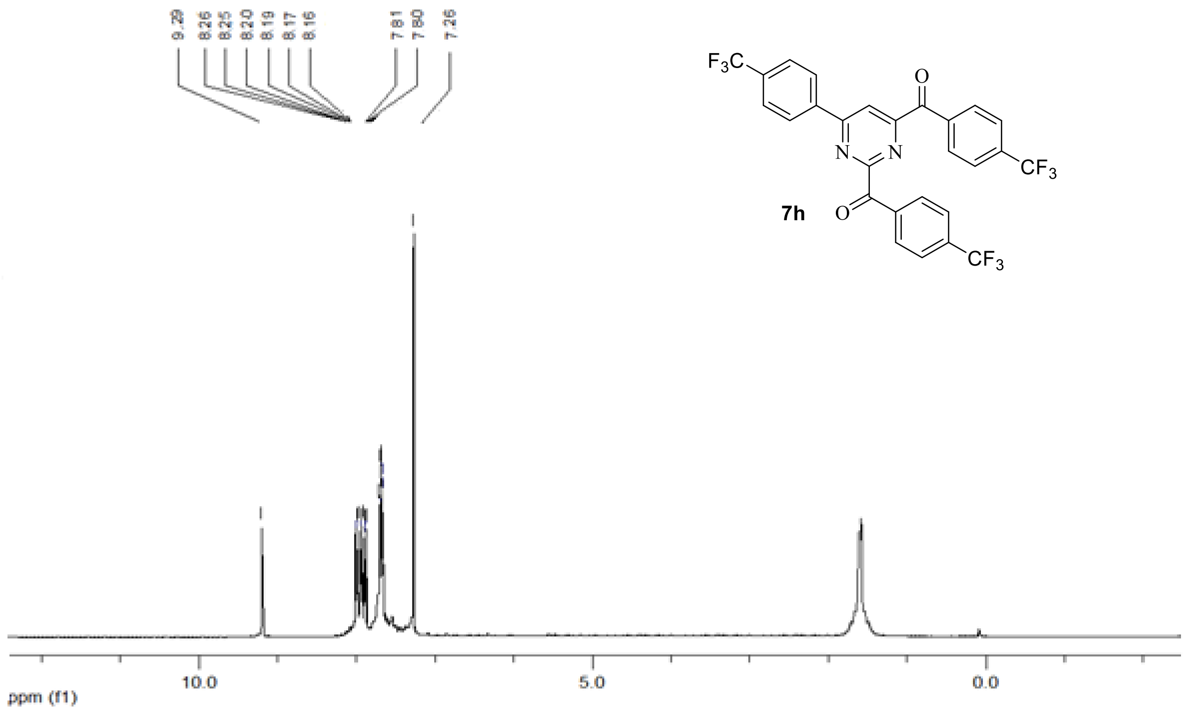

^{13}C NMR (150 MHz, $CDCl_3$)

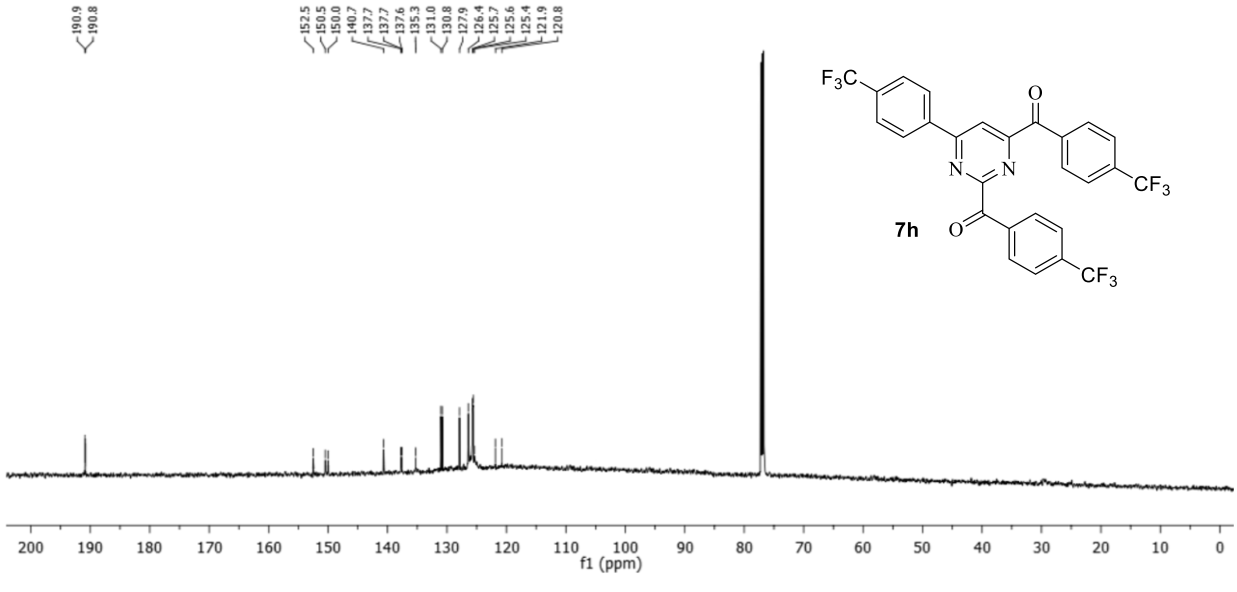

Supplement: File 1 — Compound characterization data and NMR spectra. [file Beilstein_J_Org_Chem-16-1915-s001.pdf]
